# Effects of Essential Oils from 24 Plant Species on *Sitophilus zeamais* Motsch (Coleoptera, Curculionidae)

**DOI:** 10.3390/insects12060532

**Published:** 2021-06-08

**Authors:** William R. Patiño-Bayona, Leidy J. Nagles Galeano, Jenifer J. Bustos Cortes, Wilman A. Delgado Ávila, Eddy Herrera Daza, Luis E. Cuca Suárez, Juliet A. Prieto-Rodríguez, Oscar J. Patiño-Ladino

**Affiliations:** 1 Department of Chemistry, Faculty of Sciences, Universidad Nacional de Colombia-Sede Bogotá, Bogotá 111321, Colombia; wrpatinob@unal.edu.co (W.R.P.-B.); lnagles@unal.edu.co (L.J.N.G.); jjbustosc@unal.edu.co (J.J.B.C.); wadelgadoa@unal.edu.co (W.A.D.Á.); lecucas@unal.edu.co (L.E.C.S.); 2 Department of Mathematics, Faculty of Engineering, Pontificia Universidad Javeriana, Bogotá 110231, Colombia; eherrera@javeriana.edu.co; 3 Department of Chemistry, Faculty of Sciences, Pontificia Universidad Javeriana, Bogotá 110231, Colombia; juliet.prieto@javeriana.edu.co

**Keywords:** essential oil, *Sitophilus zeamais*, fumigant toxicity, topic toxicity, repellent

## Abstract

**Simple Summary:**

The maize weevil (*Sitophilus zeamais* Motsch) is a major pest in stored grain, responsible for significant economic losses and having a negative impact on food security. Due to the harmful effects of traditional chemical controls, it has become necessary to find new insecticides that are both effective and safe. In this sense, plant-derived products such as essential oils (EOs) appear to be appropriate alternatives. Therefore, laboratory assays were carried out to determine the chemical compositions, as well as the bioactivities, of various EOs extracted from aromatic plants on the maize weevil. The results showed that the tested EOs were toxic by contact and/or fumigance, and many of them had a strong repellent effect. Samples of 14 EOs and 17 of their main constituents (monoterpenes) had high fumigant toxicity against *S. zeamais* adults and might constitute a viable control method of this pest.

**Abstract:**

Chemical control of the maize weevil (*Sitophilus zeamais*) has been ineffective and presents serious collateral damage. Among plant-derived insecticides, essential oils (EOs) are suitable candidates to control this stored products pest. In this work, the insecticidal activities of 45 natural EOs against *S. zeamais* adults were screened, and the most promising ones (24 EOs) were characterized by GC–MS. The repellent and toxic effects (contact and fumigant) of these 24 EOs were determined, and by a cluster analysis they were classified into two groups considering its fumigant activity and contact toxicity. For the EOs with the highest fumigant potential (14 oils) and their main active constituents (17 compounds), lethal concentrations were determined. The most active EOs were those obtained from *L. stoechas* and *L. alba*, with LC_50_ values of 303.4 and 254.1 µL/L air and characterized by a high content of monoterpenes. Regarding the major compounds, the oxygenated monoterpenes R-(+)-pulegone (LC_50_ = 0.580 mg/L air), S-(-)-pulegone (LC_50_ = 0.971 mg/L air) and R-(-)-carvone (LC_50_ = 1.423 mg/L air) were the most active, as few variations in their concentrations significantly increased insect mortality.

## 1. Introduction

Cereal production is essential to guaranteeing food security because cereals constitute between 50–60% of the human diet and are also an excellent source of macro and micronutrients [1]. An advantage of these products is their possibility for being stored for long periods of time without losing their nutritional value; therefore, they are available all throughout the year [2]. Despite this, it is during the post-harvest and storage stages where further deterioration occurs, mainly through pest insects, which cause losses of between 20–30% of the product in the tropical and subtropical areas of the world [3].

The maize weevil, *Sitophilus zeamais* Motsch (Coleoptera: Curculionidae), is one of the most important pests of stored grains, responsible for the primary damage of maize [4]. It is estimated that an average of two insects per grain generate 18.3% losses in 48 days. The greatest damage to the grain is caused by larvae and adults; eggs are laid during most of the adult life, although 50% can be laid in the first 5 weeks of an adult’s life [5,6]. The female drills the grain to oviposit in small, chewed cavities that are subsequently sealed by a secretion, protecting the individual to complete their life cycle within it. Owing to this, most strategies are focused on adult control [7,8]. Infestations of this species result in weight loss, decreased germination power, and reduced levels of nutrients, taste, and smell in the grains [9]. In addition, high temperature and humidity conditions allow the proliferation of other insect species and microorganisms [10,11]. Different strategies are used to control *S. zeamais*, chemical control being the most effective one. A factor that plays an important role in chemical control is the mode of application, which depends on the ecology of the insect as well as the characteristics of the place or product where the treatment is to be applied. In storing grain in bulk in warehouses or silos it is difficult to prevent and control insect infestations [10]. In this sense, insecticides with fumigant action are the most effective method for controlling pests in stored products, because due to their high volatility they can spread throughout the air space of the silo and reach areas that would not otherwise be accessible [12]. However, commercial pesticides are usually not very selective, and many are toxic to the environment and harmful to health [13,14]. Therefore, the development of effective and safe alternatives for the control of this stored grain pest is required.

Nowadays, interest in bioprospecting studies has increased as a potential strategy to find new applications for biodiversity. Furthermore, countries privileged with natural resources might improve their inherent capacities to strengthen economic activities such as safer and healthier agriculture [15]. In this sense, essential oils (EOs) from plants could be alternative sources for pest management because of their different biological activities, biodegradability, and minimal effects on non-target organisms and the environment [8,13,15]. EOs are complex, biodegradable, volatile, and lipophilic mixtures of terpenoids (monoterpenoids and sesquiterpenoids) and phenylpropanoids mainly [16]. Different studies report that EOs components cause a toxic effect in insects by contact, ingestion, or fumigation. They also produce other behavioral effects such as repellency, food deterrence, and inhibition of oviposition and growth [17,18]. It is important to consider that bioactivity of EOs is directly related to their chemical composition, which can vary dramatically, even within the same plant species. In fact, sources of compositional variability can include the plant part extracted, the phenological state of the plant, and the season, as well as growth environment conditions [19]. Therefore, research regarding the chemical components of EOs is gaining importance.

Thus, this study aimed to evaluate the insecticidal activity of EOs from 45 species of plants present in Colombia, belonging to 16 families, against adult weevils of *S. zeamais*. First, preliminary insecticidal activity was evaluated to select the most active EOs (24) and perform their chemical characterization. Then, to establish if the mortality of EOs was caused by fumigant or contact toxicity, a test in which contact between the insect and the oil was avoided was performed, as well as making a topical contact assay. Later, a cluster statistical analysis was carried out to group the EOs according to their insecticidal activity (fumigant and contact toxicity) and repellence. To the group of EOs that mainly exerted a fumigant effect, lethal concentrations (LC) were estimated. Finally, the compounds present in EOs with the best fumigant effect were selected, and their fumigant activity was also evaluated.

## 2. Materials and Methods

### 2.1. Selection of Essential Oils with Potential Insecticidal Ctivity on Sitophilus zeamais

#### 2.1.1. Plant Material

The samples of 45 aromatic plants, including wild and domesticated, were randomly collected during different field trips in the departments of Cundinamarca, Boyacá, and Santander (Colombia) or acquired in the marketplace of Samper Mendoza (Bogotá, Colombia). A specimen of each sample gathered was sent to a herbarium (Herbario Nacional Colombiano and/or Jardín Botánico José Celestino Mutis) for taxonomic determination (Table S1).

#### 2.1.2. Extraction of Essential Oils

The different parts of each chosen plant were subjected to steam extraction for 2 h. The EOs were recovered by condensation using a Clevenger-type apparatus, and after decantation, they were dried with anhydrous sodium sulfate and stored in amber-sealed glass bottles for refrigeration at 4 °C until use.

#### 2.1.3. Insects

*S. zeamais* adults were obtained from a colony maintained in the research group Química de Productos Naturales Vegetales Bioctivos (QuiProNaB) of the Department of Chemistry of the Universidad Nacional de Colombia—Bogotá. The insects were kept in corn mixtures of ICA (Instituto Colombiano Agropecuario) variety 508 and yellow corn ICA variety 105 and arranged in a culture chamber under controlled conditions for temperature (27 ± 1 °C), humidity (65 ± 5% RH), and darkness [5]. Adult insects, between 6–10 days after emergence, were used in the different activity tests.

#### 2.1.4. Preliminary Insecticidal Activity

To select the most active EOs, preliminary insecticidal activity was determined by the “*vial in vial*” method reported in the literature [20]. A volume of 11 µL of EOs was applied to a 2 cm diameter Whatman^®^ No. 1 filter paper placed on top of a 1.5 mL glass vial. Subsequently, the vial was introduced into a 22 mL vial with a screw-type closure containing 10 insects without sexing, leaving a final concentration of essential oils of 500 μL/L air. As positive controls, Nuvan 50^®^ (Sanigral Ltda. Bogotá, Colombia), containing dichlorvox as the active ingredient (100 μL/L air), and Fosfamin^®^ (Fumitoro Ltda. Bogotá, Colombia), with phosphine as the active ingredient (150 µL/L air), were used. The negative control was applied in the same way, but without the addition of any substance. All tests were performed in triplicate under controlled temperature and humidity conditions (27 ± 1 °C and 65 ± 5% HR). Insect mortality was determined after 24 h. The insects were considered dead when observed with a stereoscope with no movement of the legs and/or the antennae seen after stimulation for 15 s with an entomological pin. The mortality percentages (%M) were calculated using the Abbott’s [21] correction formula:

(1) $$\%Mortality=\left[\frac{\%Mt-\%Mc}{100-\%Mc}\right]*100$$

where *Mt* = mortality with treatment and *Mc* = mortality with control.

Essential oils with mortality percent ≥40% were selected to continue the study. To determine their chemical composition and which of the active EOs exerted a purely fumigant action, a test which avoided contact of the insect with the oil and a topical contacts assay were performed.

### 2.2. Chemical Characterization of Essential Oils

#### 2.2.1. Sample Preparation

A volume of 25 µL of each EO was taken and brought to a final volume of 1 mL with n-hexane or dichloromethane. The standard hydrocarbon solution was prepared by dissolving 25 µL of a homologous hydrocarbon solution (C_8_–C_26_) to a final volume of 1 mL with *n*-hexane.

#### 2.2.2. Analysis by GC-MS

The chromatographic analysis was performed using an Agilent Technologies 6850 II series gas chromatograph with selective mass detector Agilent Technologies MSD5975B, which was operated at 70 eV, using a quadrupole analyzer in full scan mode at 4.57 scan s^−1^. Mass spectra were acquired between 40 and 400 *m*/*z*. Two different analyses of the essential oils were performed, using two orthogonal polarity columns: DB-5MS and HP-INNOWax.

In the first analysis, a DB-5MS column ((5%)-phenyl-methylpolysiloxane, 60 m × 0.25 mm × 0.25 µm) was used, with injection in Split mode (20:1) for 1.5 min. The temperature ramp started at 40 °C for 2 min, and then it was increased to 123 °C (4 °C/min) and remained constant for 2 min. Afterward, it increased to 160 °C (4 °C/min), remained constant for 5 min, was subsequently increased to 220 °C (5 °C/min), and then kept constant for 8 min. Finally, it was increased to 280 °C (5 °C/min) and kept constant for 4 min, for a total run time of 75 min. In the second analysis, a HP-INNOWax column (polyethylene glycol (PEG), 60 m × 0.25 mm × 0.25 µm) was used, with injection in Split mode (20:1) for 1.5 min. The temperature ramp started at 45 °C for 4 min, and then it was increased to 120 °C (3 °C/min) and remained constant for 2 min. Finally, it was increased to 250 °C (4 °C/min) and kept constant for 8 min, for a total time of 71.5 min. The injection volume used in each analysis was 1 µL.

#### 2.2.3. Determination of Chemical Composition

The chemical constituents were determined by comparing the mass spectra and retention indices obtained for each compound with those reported in the Pherobase database [22] and those published in the literature [23]. The linear retention indices (RI) were calculated, using a homologous series of hydrocarbons from C_8_ to C_26_, and eluted under the same operational conditions described for EOs.

### 2.3. Determination of the Insecticidal and Repellent Effect

#### 2.3.1. Reagents

The pure compounds and the alkanes solution (C_8_–C_26_) standard (1000 ppm) were purchased at Sigma–Aldrich© (Saint Louis, MO, USA) or Merck © (Darmstadt, Germany). The compounds examined in the fumigant bioassay against S. zeamais were DL-limonene (purity 95%), α-pinene (purity 97%), β-pinene (purity 98%), 1,8-cineole (purity 99%), 1R-(-)-fenchone (purity 98%), S-(+)-carvone (purity 96%), R-(-)-carvone (purity 98%), terpinolene (purity 85%), Δ-3-carene (purity 90%), (-)-terpinen-4-ol (purity 95%), p-cymene (purity 90%), R-(+)-pulegone (purity 90%), S-(-)-pulegone (purity 98%), γ-terpinene (purity 97%), sabinene (purity 75%), α-phellandrene (purity 95%), linalool (purity 97%), α-terpinene (purity 90%) and β-caryophyllene (purity 80%).

#### 2.3.2. Fumigant Activity Test

Essential oils with a mortality percent ≥40% (24 EOs) and 19 of their individual constituents (ICs) were subjected to fumigant activity evaluation by the modified “*vial in vial*” method. In this test, contact of the insect with the EO or IC was avoided. To achieve this, the vial with the paper impregnated with EO was placed and covered with a sheer curtain or a 15% PTFE solution [24]. The same conditions of the previous trial were reproduced to evaluate the EOs: 11 µL (500 µL/L air) of EO, 24-h mortality reading, and three replicates. For the ICs, quantities between 3.0–5.0 μL were applied in 1.5 cm-diameter filter paper rings (Whatman^®^) located at the bottom of the screw cap of a 22 mL amber vial, leaving a final concentration of 150 ppm (0.6–1.1 mM (mmol/L of air)). EOs and ICs with a mortality percent ≥60% were selected for continuing the study. The percentage of mortality was calculated using Equation (1). All treatments were replicated five times under the same temperature and humidity conditions. To determine if there were significant differences between the mortality caused by EOs in the preliminary insecticidal activity assay and in the fumigant assay, a non-parametric analysis was performed with the Mann–Whitney test.

To obtain results that allowed for estimating the lethal concentrations, different quantities of EOs or ICs were used. For EO quantities, between 1.1–18 µL of EO (50–818 µL/L air) were employed. In the case of compounds, the filter papers were impregnated with different amounts (0.10–4.00 μL) of pure compound and placed in the bottom of the screw cap of a glass vial of 22, 140, 270, or 518 mL, according to each concentration [5]. The test was carried out with five replicates and two repetitions under the same temperature and humidity conditions. The lethal concentrations LC_30_, LC_50_, and LC_90_ of the EOs and ICs were estimated by Probit analysis, using the R software with a general public license. Different quantiles of EOs and their compounds were also estimated, using the same statistical program.

#### 2.3.3. Topical Contact Toxicity Test

The contact toxicity was determined by the topical contact method, which consisted of applying different amounts of EOs (0.10, 0.15, and 0.20 µL) with a micro-syringe on the insect’s prothorax. Untreated insects were used as negative controls, and Nuvan 50^®^ was used as a positive control at a volume of 0.10 µL. The treated insects were transferred to 22 mL glass vials, leaving 10 insects per vial. The vials were kept in the culture chamber under controlled temperature and humidity conditions (27 ± 1 °C and 65 ± 5% RH). All treatments were performed in triplicate, and insect mortality was determined after 24 h [25]. The percentage of mortality was calculated using Equation (1).

#### 2.3.4. Repellent Activity Test

**Figure 1:**
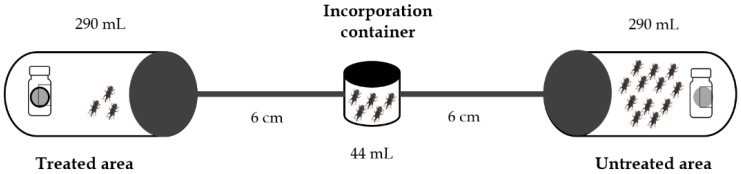
Olfactometer design employed for repellent assay (Own authorship based on data from [26]).

The repellent action was measured using an olfactometer with static air, consisting of two 290 mL bottles connected by a tube (l = 6 cm, d = 0.7 cm), with a container (44 mL) located in the central part of the duct (Figure 1). In one of the bottles corresponding to the treatment, a 1.5 mL vial was placed that had a 2 cm diameter Whatman^®^ No. 1 paper disc impregned with different volumes of EO, corresponding to concentrations between 6.2–22.7 μL/L of air. In the other bottle was placed a 1.5 mL vial with paper without EO, and this acted as a control. Adult *S. zeamais* insects (20 per assembly) were incorporated through the central container of the connecting tube. The test was executed in darkness, and the olfactometer was rotated in order to make sure that the behavior of the insects only depended on the repellent action. The activity reading was done at 2, 6, and 24 h after the application, and the number of insects present in both containers (treated and untreated) were recorded. All treatments were performed in triplicate [26]. The repellency percentage (RP) was calculated as

(2) $$RP=\frac{N-T}{N+T}*100\%.\;$$

where *N* = number of insects present in the untreated area and *T* = number of insects in the treated area.

The repellency at 2 h, 6 h, and 24 h for each of the concentrations evaluated was classified according to the following repellency scale (% repellency = class): (<0.1 = 0), (0.1–20 = I), (20.1–40.0 = II), (40.1–60.0 = III), (60.1–80.0 = IV), and (80.1–100 = V) [27].

#### 2.3.5. Statistical Analysis

With the data obtained from the fumigant, contact, and repellent assays as well as the chemical profiles of the EOs, a cluster analysis was completed. The methodology that was used corresponded to a hierarchical method with a measure of similarity and a method of grouping through partitions known as *k-means*.

Based on classification using the cluster method, for the final sample, composed of 14 EOs with the highest fumigant potential, the lethal concentrations (LC_30_, LC_50_, and LC_90_) were estimated by adjusting a Probit model. Subsequently, an analysis of variance (ANOVA) was performed to determine if there were significant differences in lethal concentrations. Then, through a post hoc analysis of Tukey, it was determined that groups of oils had marked differences in lethal concentrations. Finally, the compounds that were most correlated with mortality were determined through the Wald statistic.

## 3. Results and Discussion

### 3.1. Selection of EOs with Potential Insecticidal Activity Against S. zeamais

**Figure 2:**
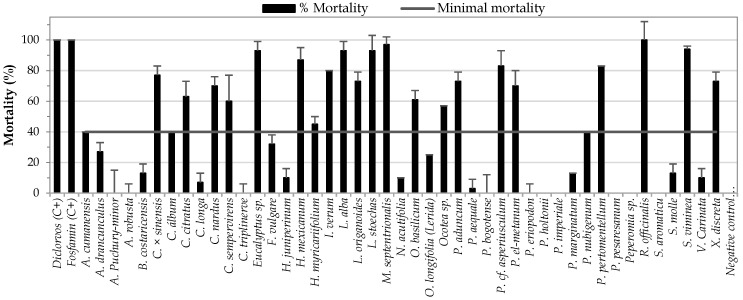
Screening of insecticidal activity for 45 EOs, evaluated at a concentration of 500 µL/L air, against *S. zeamais*.

The preliminary insecticidal assay performed for EOs from 45 plant species belonging to 16 families, using the “*vial in vial*” method at a maximum concentration of 500 µL/L air, allowed identification of 24 EOs with potential insecticidal activity that caused mortality ≥40% (Figure 2). The EOs that exhibited the highest mortality rates (>90%) were those extracted from *R. officinalis*, *L. stoechas*, *S. viminea*, *M. septentrionalis*, *Eucalyptus* sp., and *L. alba*. For the *R. officinalis* EO, it has been reported that it exerts fumigant, repellent, and toxic effects upon contact with different species of the genus *Sitophilus*, effects that have been attributed to the presence of 1,8-cineole and camphor [28,29]. Essential oils of the genus *Eucalyptus* with a high content of 1,8-cineole have also exhibited fumigant and repellent activity against species of the genus *Sitophilus* [30,31]. In addition, potential toxic contact activity on *S. zeamais* has been reported for the essential oil of *L. alba*, an effect attributed to its high limonene content [32]. For the essential oils of *M. septentrionalis* (%M = 97), *S. viminea* (%M = 94), *L. stoechas* (%M = 93), *X. discreta* (%M = 73), *P. el-metanum* (%M = 70), *A. cumanensis* (%M = 40) and *P. nubigenum* (%M = 40), the insecticidal activity on *S. zeamais* is reported for the first time.

### 3.2. Chemical Characterization of EOs with Potential Insecticidal Activity

**Figure 3:**
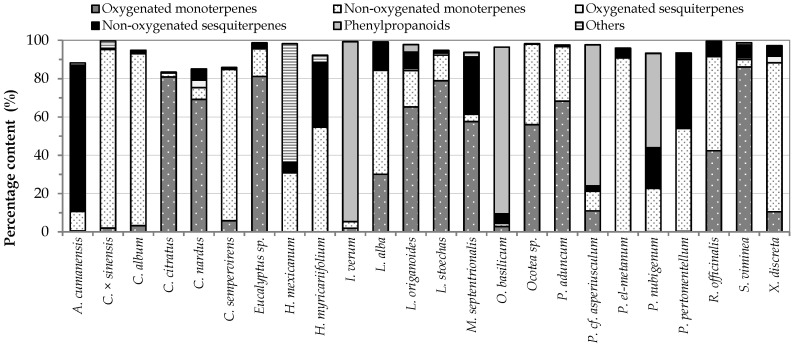
Content percentages of the main chemical classes of components in the 24 active EOs.

The GC–MS analysis with orthogonal polarity columns of the 24 EOs allowed the identification of 166 compounds, corresponding to 83–99% of the total composition. In Figure 3, percentage contents of the main chemical classes of the essential oil components are reported. In Table S2, the compounds are presented according to the elution order obtained with the DB-5MS column; the retention index for both columns (DB-5MS and HP-INNOWax), the refractive index, and the density for each of the EOs are also reported.

As presented in Figure 3, the chemical composition of the EOs was varied, highlighting monoterpenoids, sesquiterpenoids, phenylpropanoids and other compounds. It was determined that from the 24 EOs selected, 75.0% of those were mainly monoterpenic (56.3% and 99.7% of the total chemical composition). Among the major monoterpenes, the presence of α-pinene, β-pinene, 1,8-cineole, limonene, carvone, camphor, and pulegone stood out, compounds for which previously were reported fumigant, repellent, and/or antifeeding activity against *S. zeamais* [10,33].

Phenylpropanoid type oils represented 16.7% of the total oils, which contained between 52.7 and 94.1% of this type of constituents. The oils belonging to this group were characterized by containing as major compounds myristicin, apiol, dilapiol and anethole, some of which present previous reports of insecticidal effects against insects of the genus *Sitophilus* [33]. Finally, EOs composed mainly by sesquiterpenoids and alkanes represented 4.1% of the total oils. For *A. cumanensis* oil, sesquiterpenes represented 86.6% of the total composition, with germacrene-D (17.25%), bicyclogermacrene (16.79%), and γ-curcumene (34.11%) as its majority compounds. In the case of *H. mexicanum* oil, alkanes represented 57.5% of its content, nonane being the most abundant one (53.08%). For all the compounds mentioned above, any type of activity on insects of the genus *Sitophilus* has been reported.

This work constitutes the first report of the chemical composition of EOs from leaves of *M. septentrionalis* and *P. nubigenum*. For the EO of the species *M. septentrionalis*, 93.78% of the chemical composition was determined, finding that this oil was characterized by containing monoterpenic ketones (menthone (8.17%) and pulegone (41.85%)) and sesquiterpenoids (β-caryophyllene (16.51%) and bicyclogermacrene (8.67%)). This class of compounds has been previously reported in other species of the genus *Minthostachys* and in the family Lamiaceae [34,35]. The EO of *P. nubigenum,* 93.30% of the chemical composition was determined and its main components were myristicin (36.68%) and apiol (11.83%), compounds that are characteristic of some species of the genus *Piper* [36,37].

In this study, for the Piperaceae family, EOs were found consisting mainly of monoterpenes and/or sesquiterpenes, which was in accordance with what had been reported in the literature for *P. pertomentellum* (cis-β-ocimene (28.49%), trans-β-ocimene (21.02%), and germacrene D (26.88%)) and *P. aduncum* (α-pinene (6.14%), limonene (6.59%), linalool (22.43%), and piperitone (45.46%)), among others [37]. For the EO of P. *el-methanum* (synonymous *P. oblicum*), it was determined that the main constituents were α-pinene (8.48%), α-phellandrene (43.47%), limonene (19.36%), and β-phellandrene (7.78%). When comparing the chemical composition with that previously reported for the EO of *P. oblicum*, great differences were found, which may be related to the origin of the species. [38]. Additionally, it was found species characterized by their high contents of phenylpropanoid type molecules, among which stand out *P. nubigenum* and *P.* cf. *asperiusculum*.

For EOs from species of the Lamiaceae family, the main constituents were of the monoterpene type (greater than 60%), except for the EO of *O. basilicum*, which was rich in phenylpropanoids (estragole (81.78%) and methyl cinnamate (5.10%)). Some examples of EOs with a high content of monoterpenes belonging to this family were *R. officinalis* (α-pinene (23.20%), camphene (8.71%), 1,8-cineole (23.22%), and camphor (13.19%)), *L. stoechas* (1,8-cineole (17.12%), fenchone (27.77%), and camphor (27.99%)), and *S. viminea* (p-ment-3-en-8-ol (45.39%) and pulegone (38.61%)) [34,35].

### 3.3. Determination of the Insecticidal and Repellent Effect of the Selected EOs

#### 3.3.1. Fumigant Toxicity

**Figure 4:**
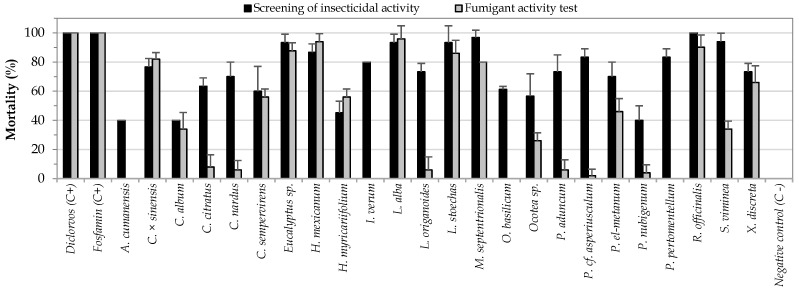
Comparisons of mortality presented in the screenings of insecticidal activity and the fumigant activity test.

To select a method for evaluating any insecticidal effect, it is important to consider whether the test conditions are adequate for the characteristics of the insects, especially in the case of the fumigant effect. Given the ability of *S. zeamais* to climb the walls of the vial and come into contact with the paper impregnated with the treatment, it was possible that the mortality of the insects was due to both contact toxicity and/or the fumigant effect. Hence, the need of differentiating between an exclusively fumigant test and a general test of insecticidal activity was evident. To determine which of the 24 EOs exerted a purely fumigant action, a test that avoided contact of the insect with the oil, was carried out. A comparison of the results of the mortality in the preliminary insecticidal assays and of the fumigant test is presented in Figure 4. The Mann-Whitney test allowed determining that there are significant differences between the mortalities caused by some essential oils in the general insecticide test and in the fumigant test (Table 1).

When comparing the results of the two methodologies used, it was evident that by avoiding contact between insects and EO, 10 of the 24 EOs showed a decrease in their toxic effects, reaching mortality rates equal to or less than 10%. For these 10 EOs, fumigant toxicity was negligible compared to contact toxicity at the tested concentrations. Among the species that lost their toxic effects avoiding the contact were mainly those belonging to the genus *Piper.* Considering the chemical composition of the 10 oils that proved not to be fumigants, it was observed that they contained phenylpropanoids (*I. verum*, *O. basilicum*, *P. nubigenium*, and *P.* cf. *asperiusculum*) or sesquiterpenes (*A. cumanensis*) as major compounds. This could lead to thinking that these types of metabolites tend to affect insects mainly by contact, probably due to their low volatility [39,40]. 

In the case of the EOs of *S. viminea*, *Ocotea* sp. and *P. el-metanum*, a 50% decrease in toxicity with respect to the preliminary insecticide assay was observed, suggesting that these EOs may have had fumigant and contact effects on the insects. Analyzing the chemical composition of these three EOs, it could be observed that they were composed, more than 90%, of monoterpenes. The first one was characterized by a high content of p-ment-3-en-8-ol (45.4%) and pulegone (38.6%), the second one mainly contained α-terpineol (44.2%), and the last one had α-phellandrene (43.5%) as the major component. The fumigant and contact activities for pulegone and fumigant activity for α-terpineol and α-phellandrene have been reported in the literature concerning *S. zeamais*. [14]. *M. septentrionalis* was also characterized by high contents of pulegone (41.9%). For this oil, there was no reduction in mortality in the fumigant assay, suggesting that the decrease in the toxic effects of *S. viminea* may have been related to the presence of p-ment-3-en-8-ol, a compound that possibly exerted a contact effect.

Like the oil of *M. septentrionalis*, the EOs of *X. discreta*, *C. sempervirens*, *H. mexicanum*, *H. myricariifolium*, *R. officinalis*, *L. stoechas*, *Eucalyptus* sp., *C. × sinensis* and *L. alba* exhibited similar activity in the two methods evaluated, indicating that their toxic effects against *S. zeamais* were fumigant. In these oils, a high content of monoterpenes was observed, except for *H. mexicanum* oil, which was mainly composed of alkanes (nonane, 53.1%). The oils mentioned above had in common the presence of limonene, α-pinene, and 1,8-cineole, compounds for which fumigant activity against *S. zeamais* had been previously reported [8,41]. In the present study, the fumigant toxicity against *S. zeamais* was reported for the first time for the EOs from the species *S. viminea*, *M. septentrionalis*, *L. alba*, *P. el-metanum*, *C. sempervirens*, *L. stoechas*, *X. discrete*, and *C. album*.

#### 3.3.2. Contact Toxicity

**Figure 5:**
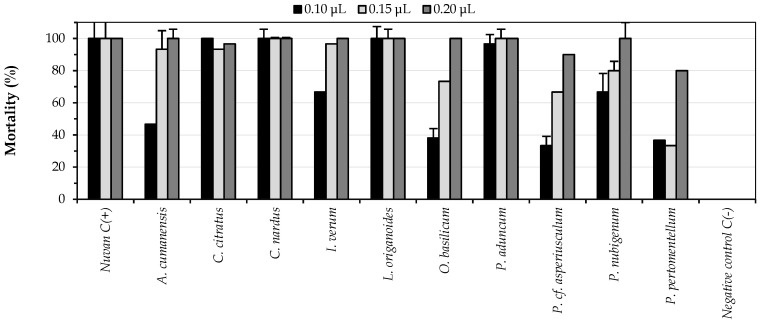
Contact toxicity results of the 10 EOs that produced fumigant mortalities less than 10%.

The fumigant test carried out against *S. zeamais* suggested that 10 of the 24 EOs could present, mainly, a contact effect because their lethal effects in the fumigant assays were less than 10%. The results of these oils in the topical contact toxicity test are illustrated in Figure 5; here, it can be seen that these 10 EOs caused mortality in ranges from 30 to 100%, confirming the observed behavior in the fumigant tests. Similarly, it was found that the oils of *P. aduncum*, *L. origanoides*, *C. nardus* and *C. citratus* were the most active by contact, causing mortality greater than 90% in the three quantities evaluated. For the remaining six EOs, it was shown that the percentage of mortality increased proportionally with the amount of oil applied.

**Figure 6:**
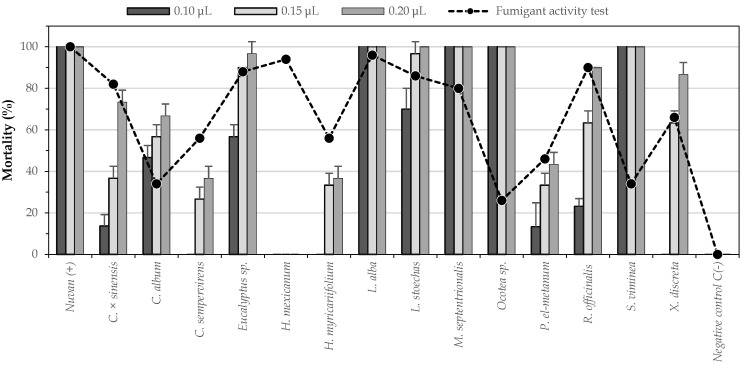
Contact toxicity results of the 14 EOs that produced fumigant mortalities greater than 25%.

The results of the contact and fumigant toxicities of the 14 EOs that presented mortality rates greater than 25% in the fumigant test are illustrated in Figure 6. It was observed that 13 EOs exhibited fumigant and contact activities, while *H. mexicanum* EO was the only one that did not cause contact mortality. The EOs from *C. × sinensis*, *C. sempervirens* and *H. myricariifolium*, although they were toxic by contact in all the quantities evaluated, caused higher mortalities when they were evaluated as fumigants at a concentration of 500 µL/L of air. In contrast, the oils from *S. viminea*, *M. septentrionalis*, *Ocotea* sp., and *C. album*, although they exhibit fumigant activity at the concentrations evaluated, caused higher mortality rates when the oils were applied directly to the insect at all the quantities tested.

In Figure 6, the contact toxicity results for the 10 AEs that did not cause high fumigant effects are presented. It is observed that the AEs corresponding to *S. viminea*, *M. septentrionalis*, *Ocotea* sp. and *L. alba* were the most active in the topical contact test, causing mortality rates higher than 90% in all the quantities evaluated. These results indicate that EOs consisting mainly of phenylpropanoids and sesquiterpenes exerted an action mainly by contact against *S. zeamais*. It has been described in structure-activity relationship studies that less volatile compounds tend to present a higher toxicity by contact [39]. In contrast, for monoterpenes, there was no clear trend because they could exert a toxic effect by fumigation, contact or both. 

The results of the contact toxicities of the EOs of *X. discreta*, *L. stoechas*, *S. viminea*, *M. septentrionalis*, *P. el-metanum*, *P. nubigenum*, *P.* cf. *asperiusculum*, *P. pertomentellum*, and *C. album* presented in this work constitute the first reports of these activities for the mentioned oils on *S. zeamais*. For the EOs of *P. aduncum*, *C. citratus*, *C. nardus*, *O. basilicum*, *R. officinalis*, *C. sempervirens* and *L. alba*, it has been reported that they exert contact toxicity against the insect of interest, with LD_50_ less than 0.5 µL/cm^2^ in contact tests with treated surfaces [42,43,44].

#### 3.3.3. Repellent Activity

Table S3 shows the olfactometer assay results, which were classified according to repellency percentages on a scale of 0 to V, 0 being an indicator of neutrality and V an indicator of high repellent power [26]. It was noted that all evaluated EOs showed repellent activity against *S. zeamais*, and it was also found that this effect showed great variation, being affected by the type of oil, the exposure time, and the concentration of oil evaluated.

The *I. verum* EO, which was characterized by a high anethole content, showed repellent percentages (RP) between 43.3 and 70.0% at 2 h of treatment and RP between 20.0 and 46.7% after 24 h. Thus, the oil was classified as a class I to III repellent, in accordance with the literature reports for oils rich in anethole, which indicated moderate levels of repellency against *S. zeamais* [45].

The EOs from *H. myricariifolium*, *L. stoechas*, *P. aduncum*, *C. nardus*, and *C. × sinensis* did not show great variation in their repellent effects at the evaluated concentrations during the 24 h of the test. These oils had the highest repellent effects against *S. zeamais*, being classified in categories IV and V. For citronellal, α-pinene, linalool, limonene and β-caryophyllene has been reported in literature their repellent action against *S. zeamais*, and these were the majority constituents in some of these EOs [30,46,47]. The EOs of *X. discreta*, *Eucalyptus* sp., *P. el-methanum*, *P. pertomentellum* and *C. citratus* exhibited the greatest repellent effect at 24 h, classifying in category V for the highest concentrations evaluated. Some of these oils were characterized by a high content of 1,8-cineole, limonene, and α-pinene, compounds for which their repellent activity against *S. zeamais* has been previously described [30,46].

Some of the EOs evaluated in this work present previous reports of repellent action against *S. zeamais*, such as is the case of those from *C. sempervirens*, *C. citratus*., *C. nardus*, *L. alba*, *O. basilicum*, *R. officinalis*, *H. mexicanum*, *H. myricariifolium*, *P. aduncum*, *P. pertomentellum* and *P.* cf. *asperiusculum* [8,42,48,49]. The repellent assay results obtained for the EOs of these species in some cases differed from those previously reported in the literature due to differences in the methods used and changes in the composition of the EOs, as well as the use of different plant organs, among others. This study constitutes the first report of repellent activity against *S. zeamais* of the EOs from *L. stoechas*, *C. album*, *X. discreta*, *M. septentrionalis*, *S. viminea*, *P. el-metanum*, *P. nubigenum*, *A. cumanensis*, and *I. verum*, being many of them classified in categories IV to V.

### 3.4. Characterization of Essential Oils with Fumigant Potential

#### 3.4.1. Statistical Analysis

An exploratory cluster analysis was carried out with the Euclidean distance model as a selection criterion to define the representative sample of EOs that have insecticidal potential, mainly fumigant. In this analysis, the initial sample corresponded to the 24 AOs that presented insecticidal effects in the general test. The results obtained for these EOs on a time scale of 24 h at the maximum concentration or dose in three treatments were included as variables: T1 (fumigant action), T2 (contact toxicity) and T3 (repellency). The cluster analysis allowed determining two main groups, according to their insecticidal activity. The first group consist of 14 EOs (*X. discreta, C. sempervirens, H. mexicanum, H. myricariifolium, R. officinalis, L stoechas, M. septentrionalis, Eucalyptus* sp., *Ocotea* sp., *C. × sinensis, L. alba, S. viminea, C. album*, and *P. el-metanum*) that showed greater fumigant action (mortalities greater than 50% with 500 µL/L air). The second group is mainly constituted by the EOs that presented the highest contact and repellent potential, except for *P. aduncum* oil that presented high contact activity and low repellent effect (33%). The EOs belonging to the second group were discarded for causing mortalities, in the fumigant method, that did not exceed 10%. It was found that in the group of 14 EOs, 75% had a percentage of mortality and repellency above 85% (Table 2).

**Figure 7:**
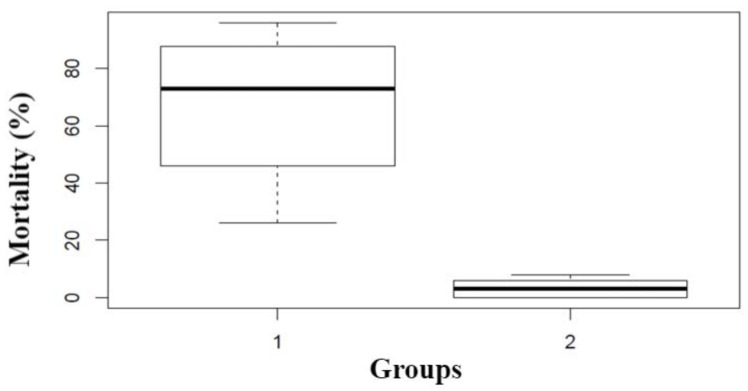
Variability in the fumigant action of the groups resulting from the cluster analysis.

To verify the section of the final sample, a Box-Plot diagram was constructed (Figure 7), in which the %mortality caused in the fumigant method is represented for the EOs that make up group I (selected EOs) and the group II (EOs discarded), observing marked differences between the two groups. In group I it is observed that approximately 25% of the EAs that comprise it can cause mortalities greater than 40% with a concentration of 500 µL/L air, and that approximately 50% of the EAs can cause mortalities greater than 70% at the same concentration. On the contrary, in group II it is found that the oils of this group cause mortalities of less than 10%.

#### 3.4.2. Determination of Lethal Concentrations of EOs with Fumigant Potential

At the 14 EOs with fumigant potential selected by the cluster analysis, the lethal concentrations (LC) on *S. zeamais* were estimated, using fumigant activity test evaluating different concentrations of each oil. Table 3 shows the fumigant toxicity results of the EOs in terms of their LC_50_, including the slope, intercept, and significance values ($\chi^{2}$ not significant, ρ > 0.05) of each of the equations that represent the regressions applied using the Probit model. It is observed that the lowest values of LC_50_ correspond to the oils of *Eucalyptus* sp., *H. mexicanum*, *R. officinalis* and *L. alba*, with concentrations lower than 255.0 μL/L air. The other essential oils evaluated presented LC_50_ between 300.0 and 650.0 μL/L air. The oil of *Eucalyptus* sp. was the most toxic because it presented the lowest median lethal concentration (LC_50_: 184.3 μL/L of air), being significantly lower than the other essential oils based on the criterion of nonoverlapping of the LC_50_ confidence intervals at 95% probability. The highest LC_50_ was observed for *Ocotea* sp. oil (LC_50_: 642.63 μL/L of air), which was 38.68 times higher than the LC_50_ estimated for *Eucalyptus* sp., being the least toxic EO by fumigance.

**Figure 8:**
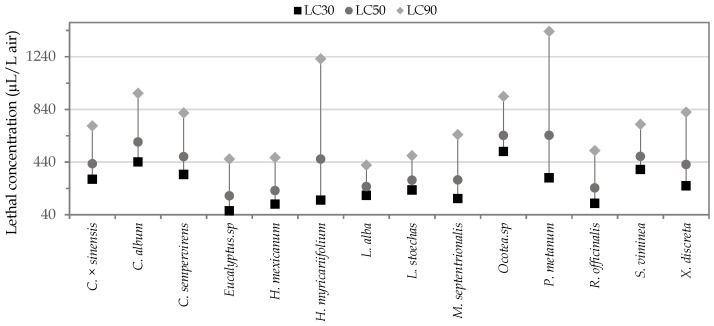
Scatter plot of the different lethal concentrations of fumigant toxicity for the 14 active EOs.

The lethal concentrations, LC_30_ and LC_90_, were estimated to make a scatter plot (Figure 8) that allowed easier visualization of the sensitivity that *S. zeamais* had to different lethal concentrations of each EO. In this sense, it was observed that for the oils of *L. alba* and *L. stoechas*, the smallest changes in the concentration of the oils were required to achieve significant changes in insect mortality. In contrast, for the oils of *H. myricariifolium* and *P. el-metanum*, high variations in their concentrations were required to achieve an appreciable change in the mortality of *S. zeamais*, with LC_90_ above 1000 μL/L of air. Additionally, it was observed that although the EO of *Eucalyptus* sp. had the lowest values of LC_30_ and LC_50_, it required a concentration of more than double the LC_50_ to reach a mortality of 90%. In this study, the fumigant LC_50_ values on *S. zeamais* are reported for the first time for the oils of *L. stoechas, R. officinalis, M. septentrionalis, X. discreta, C. sempervirens, S. viminea, C. album*, and *P. el-mentanum*.

#### 3.4.3. Fumigant Potential of Some Individual Components (ICs)

**Figure 9:**
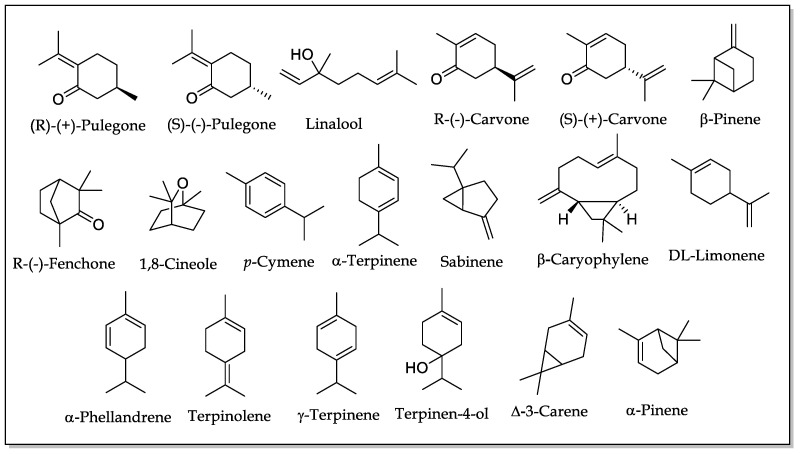
Chemical structures of the 19 ICs evaluated in the fumigant test.

To estimate which of the chemical constituents of EOs may be responsible for the fumigant effects against *S. zeamais*, the Wald statistical method was applied (with a significance level of 1%). This analysis did not allow discrimination between the possible compounds responsible for the fumigant effect, as it suggested that the 166 chemical constituents determined in this study are highly significant in the fumigant toxicity. It is important to mention that Wald’s method does not consider interactions of any kind due to multicollinearity problems in the data [50]. Therefore, in this study, 19 major ICs present in the EOs with the highest fumigant potential (LC50 < 255 μL/L air) (Table 4) were selected, using as selection criteria their presence in more than one EO and the possibility of being purchased commercially (Figure 9).

Among the 19 ICs evaluated, 17 presented fumigant potential, causing mortalities higher than 70% at a concentration of 150 mg/L. The compounds 1,8-cineole, sabinene, α-pinene and limonene presented high fumigant activity (mortality > 80%) and were present as major components in more than three active EOs. Other constituents that were found as the majority in at least two EOs with high fumigant activity were β-pinene, R-(+)-pulegone, and S-(-)-pulegone, which presented a mortality of 100% at maximum concentration evaluated. Compounds that were presented on at least one potentially fumigant EO such as α-phellandrene, γ-terpinene, R-(-) fenchone, ∆-3-carene, terpinolene, and carvone (R and S) also showed stronger fumigant action. The compounds that were present in more than one EO, but in a low proportion, such as *p*-cymene, α-terpinene, and (-)-terpinen-4-ol, revealed a mortality of 100%. This indicated that the fumigant capacity of the EOs was not only due to their majority components.

Table 5 reports the LC_50_ for the 17 active ICs, as well as the slope values and significance of each of the equations that represent the regressions applied to the selected compounds using a Probit analysis. All active compounds had LC_50_ lower than 120 mg/L air; among them, the monoterpenic ketones R-(+)-pulegone, S-(-)-pulegone, and R-(-)-carvone stood out because they exhibited lower LC_50_ than dichlorvos (positive control). Additionally, they were the compounds with greater slopes, indicating that small changes in concentration were required to increase mortality against *S. zeamais*. Other compounds with moderate fumigant effect like (-)-terpinen-4-ol, S-(+)-carvone, R-(-)-fenchone, terpinolene, and 1,8-cineole also did not require large changes in concentration to increase their fumigant capacity. Fumigant activity against species of the genus *Sitophilus* has been previously reported in the literature for these compounds, with LC_50_ between 19.0 and 30.6 μL/L air [51,52,53]. In this study, the values of fumigant LC_50_ against *S. zeamais* for S-(-)-pulegone and R-(-)-fenchone are reported for the first time.

The results presented in this study demonstrate that the group of EOs with prominent fumigant activity and, in particular, its main monoterpenic components have the potential to be used in the development of useful products for the control of *S. zeamais*. However, in future research is necessary to characterize and validate their potential applications, as well as to estimate the economic and social viability of potential insecticides based on their possible sustainable productive use.

## 4. Conclusions

This study contributes to the chemical and insecticidal characterization of EOs from aromatic plants and shows their potential as effective alternatives for the control of *S. zeamais*. The results indicated that the oils with the greatest fumigant potential were those obtained from *L. stoechas* and *L. alba*. The oxygenated monoterpenes R-(+)-pulegone, S-(-)-pulegone, and R-(-)-carvone were the ICs with the highest fumigant potential. These substances (EOs and ICs) had low lethal concentrations and did not require large variations in their concentrations to significantly increase insect mortality. The EOs with higher contact toxicity were characterized by a high content of sesquiterpenoids and phenylpropanoids. Although most EOs had a high percentage of repellency, there was no clear link between chemical composition and repellent effect.

## Figures and Tables

**Table 1:** Results of the Mann–Whitney test for the comparison of mortality presented in the screening of insecticidal activity and the fumigant activity test.

Species	Mann–Whitney Test *p* Value	Species	Mann–Whitney Test *p* Value	Species	Mann–Whitney Test *p* Value
*A. cumanensis*	*p* < 0.0001 *	*H. myricarifolium*	*p* > 0.0500	*P. aduncum*	*p* < 0.0160 *
*C. × sinensis*	*p* > 0.0500	*I. verum*	*p* < 0.0001 *	*P.* cf. *asperiusculum*	*p* < 0.0160 *
*C. album*	*p* > 0.4640	*L. alba*	*p* > 0.0500	*P. el-metanum*	*p* < 0.0350 *
*C. citratus*	*p* < 0.0330 *	*L. origanoides*	*p* < 0.0330 *	*P. nubigenum*	*p* < 0.0350 *
*C. nardus*	*p* < 0.0260 *	*L. stoechas*	*p* > 0.0500	*P. pertomentellum*	*p* < 0.0350 *
*C. sempervirens*	*p* > 0.0500	*M. septentrionalis*	*p* < 0.0160 *	*R. officinalis*	*p* < 0.0350 *
*Eucalyptus sp*	*p* > 0.0500	*O. basilicum*	*p* < 0.0100 *	*S. viminea*	*p* < 0.0350 *
*H. mexicanum*	*p* > 0.0500	*Ocotea sp.*	*p* < 0.0160 *	*X. discrete*	*p* > 0.0500

* There was a significant difference between the mortality results for the compared assays.

**Table 2:** Statistical parameters of cluster analysis.

Parameter	% Contact Mortality	% Fumigant Mortality	% Repellent Action
	[0.2 μL]	[11 μL]	[22 μL]
Median	88.35	73.00	79.90
Coefficient of variation (n − 1)	0.44	0.42	0.16
Asymmetry (Fisher)	−1.12	−0.74	−1.18

**Table 3:** Fumigant toxicity for the 14 EOs, expressed as median lethal concentrations (LC_50_).

Species	LC_50_^a^ (95% Confidence Limit) μL/L Air	Slope ^b^	Intercept ^c^	*p*-Value ^d^
*C. × sinensis*	427.8 (382.3–468.8)	0.004	−1.907	$8.04\times 10^{-19}$
*C. album*	593.0 (494.5–653.7)	0.003	−2.055	$3.10\times 10^{-5}$
*C. sempervirens*	481.6 (416.8–531.9)	0.004	−1.863	$7.69\times 10^{-11}$
*Eucalyptus* sp.	184.3 (139.0–222.1)	0.005	−0.893	$9.48\times 10^{-14}$
* *H. mexicanum*	223.5 (173.6–262.0)	0.005	−1.140	$1.60\times 10^{-13}$
* *H. myricariifolium*	463.1 (338.3–559.9)	0.002	−0.779	$2.10\times 10^{-8}$
*L. alba*	254.1 (229.1–279.6)	0.008	−2.005	$4.65\times 10^{-21}$
*L. stoechas*	303.4 (276.0–332.7)	0.007	−2.103	$4.70\times 10^{-21}$
*M. septentrionalis*	304.4 (249.9–350.5)	0.004	−1.130	$3.08\times 10^{-13}$
*Ocotea* sp.	642.6 (597.5–692.7)	0.004	−2.770	$4.14\times 10^{-13}$
*P. el-metanum*	643.9 (528.5–825.0)	0.002	−1.046	$1.54\times 10^{-3}$
*R. officinalis*	243.7 (204.4–282.3)	0.005	−1.097	$9.48\times 10^{-14}$
*S. viminea*	483.6 (432.9–545.6)	0.005	−2.540	$1.51\times 10^{-14}$
*X. discreta*	422.3 (358.9–480.8)	0.003	−1.365	$4.96\times 10^{-8}$

^a^ concentration that caused 50% of the mortality (5 replicate data); ^b^ slope of the linear regression of concentration–mortality; ^c^ intercept of the linear regression of concentration–mortality; ^d^ significance (α < 0.05); * previously reported [8].

**Table 4:** Percentages of the main components of the 14 EOs with potential fumigant activity on *S. zeamais*.

Component	RI *	*C. album*	*C. sempervirens*	*C. × sinensis*	*Eucalyptus* sp.	*H. mexicanum*	*H. myricariifolium*	*L. alba*	*L. stoechas*	*M. septentrionalis*	*Ocotea* sp.	*P. el-metanum*	*R. officinalis*	*S. viminea*	*X. discreta*
Nonane	904					53.08									
α-Pinene	939	7.12	16.89			25.28	42.52				24.01		23.20		24.71
Camphene	956												8.71		
Sabinene	976		9.39												5.08
β-Pinene	983	12.42													36.04
α-Phellandrene	1010											43.47			
∆-3-Carene	1012		11.93												
α-Terpinene	1020		4.85												
*p*-Cymene	1028											4.96			3.40
Limonene	1033		6.58	91.22	6.50			53.10			5.93	19.36			3.01
cis β-ocimene	1035	18.05													
β-Phellandrene	1036	37.62	7.07									7.78			
1,8-Cineole	1038				66.65				17.12				23.20		10.06
γ-Terpinene	1060		5.72												
α-Terpinolene	1087		6.58												
Fenchone	1094								27.77						
Camphor	1155								27.99				13.19		
*p*-Ment-3-en-8-ol	1158													45.39	
Menthone	1161									8.17					
Terpinen-4-ol	1186	0.22	0.79		0.31				0.48		1.09		0.39		0.06
α-Terpineol	1201										44.29				
Pulegone	1252									41.85				38.61	
Carvone	1256							27.94							
Terpinyl acetate	1354				12.20										
β-Caryophyllene	1430						13.59			16.51					
α-Caryophyllene	1465						4.69								
Germacrene D	1491							11.20							
Abietadiene	1969		8.35												

* Non-polar column DB-5MS; the numbers within the table correspond to the relative percentages of each compound in the EO.

**Table 5:** Fumigant toxicities for the 17 active ICs, expressed as median lethal concentrations (LC_50_).

Compound	LC_50_^a^ (95% Confidence Limit)		Slope ^b^	*p*-Value ^c^
	mg/L Air	µmol/L Air		
R-(+)-Pulegone	0.58	3.81	3.73	9.90 × 10^−6^
	(0.46–0.71)	(3.03–4.69)	(±0.84)	
S-(-)-Pulegone	0.97	6.37	1.79	4.50 × 10^−5^
	(0.69–1.21)	(4.54–7.98)	(±0.44)	
R-(-)-Carvone	1.42	9.48	1.73	1.30 × 10^−5^
	(1.14–1.72)	(7.57–11.48)	(±0.40)	
S-(+)-Carvone	2.87	19.10	0.52	2.00 × 10^−4^
	(1.99–3.75)	(13.26–24.99)	(±0.14)	
(-)-Terpinen-4-ol	4.03	26.14	0.61	4.40 × 10^−5^
	(3.27–4.91)	(21.22–31.82)	(±0.15)	
R-(-)-Fenchone	10.59	69.59	0.20	1.50 × 10^−5^
	(8.56–13.16)	(56.24–86.45)	(±0.046)	
1,8-Cineole	12.96	84.04	0.15	2.60 × 10^−6^
	(10.38–16.10)	(67.31–104.36)	(±0.031)	
*p*-Cymene	28.68	213.67	0.07	1.20 × 10^−5^
	(23.08–35.59)	(171.95–256.12)	(±0.016)	
Terpinolene	52.13	337.92	0.035	1.33 × 10^−3^
	(34.83–72.33)	(225.81–468.91)	(±0.012)	
α-Terpinene	60.24	442.21	0.04	1.80 × 10^−6^
	(47.80–72.75)	(350.86–533.98)	(±0.007)	
Sabinene	68.65	503.92	0.04	2.70 × 10^−6^
	(56.89–79.75)	(417.59–585.44)	(±0.008)	
∆-3-Carene	80.35	589.79	0.02	8.70 × 10^−7^
	(64.09–97.18)	(470.47–713.38)	(±0.005)	
Limonene	88.69	651.06	0.03	3.10 × 10^−7^
	(74.72–103.70)	(548.49–761.24)	(±0.005)	
α-Phellandrene	88.87	652.36	0.02	4.50 × 10^−5^
	(69.70–108.27)	(511.64–794.79)	(±0.006)	
β-Pinene	97.60	716.41	0.02	1.10 × 10^−5^
	(77.15–116.76)	(566.36–857.12)	(±0.005)	
γ- Terpinene	107.95	792.42	0.02	8.30 × 10^−4^
	(82.31–146.97)	(604.23–1078.84)	(±0.005)	
α-Pinene	110.38	810.25	0.02	3.50 × 10^−6^
	(90.76–130.60)	(666.22–958.70)	(±0.004)	
Dichlorvos (C +)	2.17	9.84	0.51	4.40 × 10^−4^
	(1.53–3.81)	(6.01–17.22)	(±0.15)	

**^a^** Concentration that caused 50% of the mortality (5 replicates); **^b^** slope of the linear regression of concentration–mortality; **^c^** significance (α < 0.05).

## Data Availability

The data presented in this study are available within the article or supplementary material.

## Supplementary Materials

The following are available online at https://www.mdpi.com/article/10.3390/insects12060532/s1: Table S1, sample information of the 45 species plants worked; Table S2: chemical compositions of the EOs of 24 spices with potential insecticidal activity; Table S3: Repellent effects of EOs from the 24 active species of plants on adults of *S. zeamais*.

## References

[1] Chaudhary D.P., Kumar S., Langyan S. (2014). Maize: Nutrition Dynamics and Novel Uses.

[2] FAO: Food and Agriculture Organization of the United Nations (2019). World Food Situation: FAO Information Note on the Supply and Demand of Cereals. Electronic Newsletter. http://www.fao.org/worldfoodsituation/csdb/es/.

[3] Paz C., Burgos V., Iturra A., Rebolledo R., Ortiz L., Baggio R., Becerra J., Cespedes-Acuña C.L. (2018). Assessment of insecticidal responses of extracts and compounds of *Drimys winteri*, *Lobelia tupa*, *Viola portalesia* and *Vestia foetida* against the granary weevil *Sitophilus granarius*. Ind Crops Prod..

[4] Nwosu L.C. (2018). Impact of Age on the Biological Activities of *Sitophilus zeamais* (Coleoptera: Curculionidae) Adults on Stored Maize: Implications for Food Security and Pest Management. J. Econ. Entomol..

[5] García D. (2009). Evaluación de Insecticidas de Cuatro Grupos Toxicológicos para el Control de *Sitophilus zeamais* Motschulsky. Ph.D. Thesis.

[6] Tefera T., Mugo S., Likhayo P., Beyene Y. (2011). Resistance of three-way cross experimental maize hybrids to post-harvest insect pests, the larger grain borer (*Prostephanus truncatus*) and maize weevil (*Sitophilus zeamais*). Int. J. Trop. Insect Sci..

[7] Pereira da Silva F., Capítulo V. (1993). Conservación y Protección de los Granos Almacenados. En *Manual de manejo poscosecha de granos a nivel rural*. Oficina Regional de la FAO para América Latina y el Caribe. http://www.fao.org/3/x5027s/x5027S0h.htm#V.%20Conservacion%20y%20proteccion%20de%20los%20granos%20almacenados.

[8] Patiño-Bayona W., Plazas E., Bustos J., Prieto J., Patiño-Ladino O. (2021). Essential Oils of Three *Hypericum* Species from Colombia: Chemical Composition, Insecticidal and Repellent Activity Against *Sitophilus zeamais* Motsch. (Coleoptera: Curculionidae). Rec. Nat. Prod..

[9] Hell K., Cardwell K.F., Setamou M., Schulthess F. (2000). Influence of insect infestation on aflatoxin contamination of stored maize in four agroecological regions in Benin. Afr. J. Entomol..

[10] Fleurat-Lessard F. (2016). Stored-Grain Pest Management. Encyclopedia of Food Grains.

[11] Nesci A., Barra P., Etcheverry M. (2011). Integrated management of insect vectors of *Aspergillus flavus* in stored maize, using synthetic antioxidants and natural phytochemicals. J. Stored Prod. Res..

[12] Thoms E.M., Busacca J.D. (2016). Fumigants. Encyclopedia of Food and Health.

[13] Boyer S., Zhang H., Lempérière G. (2012). A review of control methods and resistance mechanisms in stored-product insects. Bull. Entomol. Res..

[14] Herrera J.M., Zunino M.P., Dambolena J.S., Pizzolitto R.P., Gañan N.A., Lucini E.I., Zygadlo J.A. (2015). Terpene ketones as natural insecticides against *Sitophilus zeamais*. Ind. Crops. Prod..

[15] Peschiutta M.L., Brito V.D., Achimón F., Zunino M.P., Usseglio V.L., Zygadlo J.A. (2019). New insecticide delivery method for the control of *Sitophilus zeamais* in stored maize. J. Stored Prod. Res..

[16] Bhavya M.L., Chandu A.G.S., Devi S.S. (2018). *Ocimum tenuiflorum* oil, a potential insecticide against rice weevil with anti-acetylcholinesterase activity. Ind. Crops. Prod..

[17] Mossa A.T.H. (2016). Green pesticides: Essential oils as biopesticides in insect-pest management. J. Environ. Sci. Technol..

[18] Bett P.K., Deng A.L., Ogendo J.O., Kariuki S.T., Kamatenesi-Mugisha M., Mihale J.M., Torto B. (2016). Chemical composition of *Cupressus lusitanica* and *Eucalyptus saligna* leaf essential oils and bioactivity against major insect pests of stored food grains. Ind. Crop. Prod..

[19] Angioni A., Barra A., Coroneo V., Dessi S., Cabras P. (2006). Chemical composition, seasonal variability, and antifungal activity of *Lavandula stoechas* L. ssp. *stoechas* essential oils from stem/leaves and flowers. J. Agric. Food. Chem..

[20] Pascual M.J., Ballesta M.C., Soler A. (2004). Toxicidad y repelencia de aceites esenciales en plagas de almacén del arroz. Boletín de Sanidad Vegetal Plagas..

[21] Abbott W.S. (1925). A Method of Computing the Effectiveness of an Insecticide. J. Econ. Entomol..

[22] The Pherobase: Database of Pheromones and Semiochemicals. http://www.pherobase.com/.

[23] Adams R.P. (2012). Identification of Essential Oil Components by Gas Chromatography/Mass Spectrometry.

[24] Chu S.S., Du S.S., Liu L.Z. (2013). Fumigant compounds from the essential oils of Chinese *Blumea bolsamifera* leaves against maize weevil *Sitophilus zeamais*. J. Chem..

[25] Liu Z.L., Goh S.H., Ho S.H. (2007). Screening of Chinese medicinal herbs for bioactivity against *Sitophilus zeamais* Motschulsky and *Tribolium castaneum* (Herbst). J. Stored Prod. Res..

[26] Ringuelet J., Ocampo R., Henning C., Padín S., Urrutia M., Dalbello G. (2014). Actividad insecticida del aceite esencial de *Lippia alba* (Mill.) N. E. Brown sobre *Tribolium castaneum* Herbst. en granos de trigo (*Triticum aestivum* L.). Rev. Bras. Agroecol..

[27] Abdel-Rahman H., Abdel-Moty H., Nabawy E., Eman I. (2011). Evaluation of Twenty Botanical Extracts and Products as Sources of Repellents, Toxicants and Protectants for Stored Grains against the Almond Moth, *Cadra cautella*. Funct. Plant. Sci. Biotechnol..

[28] Kiran S., Prakash B. (2015). Toxicity and biochemical efficacy of chemically characterized *Rosmarinus officinalis* essential oil against *Sitophilus oryzae* and *Oryzaephilus surinamensis*. Ind. Crops. Prod..

[29] Bedini S., Guarino S., Echeverria M.C., Flamini G., Ascrizzi R., Loni A., Conti B. (2020). *Allium sativum*, *Rosmarinus officinalis*, and *Salvia officinalis* essential oils: A spiced shield against blowflies. Insects.

[30] Karimi C.K., Ndung’ M.W., Githua M. (2013). Repellent effects of essential oils from selected *Eucalyptus* species and their major constituents against *Sitophilus zeamais* (Coleoptera: Curculionidae). Int. J. Trop. Insect. Sc..

[31] Mossi A.J., Astolfi V., Kubiak G., Lerin L., Zanella C., Toniazzo G., Oliveira D., Devilla I., Cansian R., Restello R. (2011). Insecticidal and repellency activity of essential oil of Eucalyptus sp. against *Sitophilus zeamais* Motschulsky (Coleoptera, Curculionidae). J. Sci. Food Agric..

[32] Kamanula J.F., Belmain S.R., Hall D.R., Farman D.I., Goyder D.J., Mvumi B.M., Masumbu F.F., Stevenson P.C. (2017). Chemical variation and insecticidal activity of *Lippia javanica* (Burm. f.) Spreng essential oil against *Sitophilus zeamais* Motschulsky. Ind. Crops Prod..

[33] Polatoğlu K., Karakoç Ö.C., Yücel Yücel Y., Gücel S., Demirci B., Başer K.H.C., Demirci F. (2016). Insecticidal activity of edible *Crithmum maritimum* L. essential oil against Coleopteran and Lepidopteran insects. Ind. Crops Prod..

[34] Carrasco A., Ortiz V., Martinez R., Tomas V., Tudela J. (2015). *Lavandula stoechas* essential oil from Spain: Aromatic profile determined by gas chromatography–mass spectrometry, antioxidant and lipoxygenase inhibitory bioactivities. Ind. Crops. Prod..

[35] Hossain F., Lacroix M., Salmieri S., Vu K., Follett P.A. (2014). Basil oil fumigation increases radiation sensitivity in adult *Sitophilus oryzae* (Coleoptera: Curculionidae). J. Stored Prod. Res..

[36] François T., Pierre J., Lambert S., Ndifor F., Arlette W. (2009). Comparative essential oils composition and insecticidal effect of different tissues of *Piper capense* L., *Piper guineense* Schum. et Thonn., *Piper nigrum* L. and *Piper umbellatum* L. grown in Cameroonatsa. Afr. J. Biotechnol..

[37] Potzernheim M.C., Bizzo H.R., Silva J.P., Vieira R.F. (2012). Chemical characterization of essential oil constituents of four populations of *Piper aduncum* L. from Distrito Federal, Brazil. Biochem. Syst. Ecol..

[38] Guerrini A., Sacchetti G., Rossi D., Paganetto G., Muzzoli M., Andreotti E., Tognolini M., Maldonado M., Bruni R. (2009). Bioactivities of *Piper aduncum* L. and *Piper obliquum* Ruiz & Pavon (Piperaceae) essential oils from Eastern Ecuador. Environ. Toxicol. Pharmacol..

[39] Dambolena J.S., Zunino M.P., Herrera J.M., Pizzolitto R.P., Areco V.A., Zygadlo J.A. (2016). Terpenes: Natural products for controlling insects of importance to human health—A structure-activity relationship study. Psyche.

[40] Jang Y.S., Yang Y.C., Choi D.S., Ahn Y.J. (2005). Vapor phase toxicity of marjoram oil compounds and their related monoterpenoids to Blattella germanica (Orthoptera: Blattellidae). J. Agric. Food Chem..

[41] Yildirim E., Emsen B., Kordali S. (2013). Insecticidal effects of monoterpenes on *Sitophilus zeamais* Motschulsky (Coleoptera: Curculionidae). J. Appl. Bot Food Qual..

[42] Peixoto M.G., Bacci L., Blank A.F., Araújo A.P.A., Alves P.B., Silva J.H.S., Santos A.A., Oliveria A.P., da Costa A.S., de Fátima Arrigoni-Blank M. (2015). Toxicity and repellency of essential oils of *Lippia alba* chemotypes and their major monoterpenes against stored grain insects. Ind. Crop. Prod..

[43] Kerdchoechuen O., Laohakunjit N., Singkornard S., Matta F.B. (2010). Essential oils from six herbal plants for biocontrol of the maize weevil. HortScience.

[44] Kim J., Park I.-K. (2008). Fumigant toxicity of Korean medicinal plant essential oils and components from *Asiasarum sieboldi* root against *Sitophilus oryzae* L.. Flavour Fragr. J..

[45] Bedini S., Bougherra H.H., Flamini G., Cosci F., Belhamel K., Ascrizzi R., Conti B. (2016). Repellency of anethole-and estragole-type fennel essential oils against stored grain pests: The different twins. Bull. Insectol..

[46] Fouad H.A., da Camara C.A.G. (2017). Chemical composition and bioactivity of peel oils from *Citrus aurantiifolia* and *Citrus reticulata* and enantiomers of their major constituent against *Sitophilus zeamais* (Coleoptera: Curculionidae). J. Stored Prod. Res..

[47] Polatoğlu K., Karakoç Ö.C. (2016). Chapter 5—Biologically Active Essential Oils against Stored Product Pests. Essential Oils in Food Preservation, Flavor and Safety.

[48] Bertoli A., Conti B., Mazzoni V., Meini L., Pistelli L. (2011). Volatile chemical composition and bioactivity of six essential oils against the stored food insect *Sitophilus zeamais* Motsch. (Coleoptera Dryophthoridae). Nat. Prod. Res..

[49] Nerio L.S., Olivero-Verbel J., Stashenko E. (2009). Repellent activity of essential oils from seven aromatic plants grown in Colombia against *Sitophilus zeamais* Motschulsky (Coleoptera). J. Stored Prod. Res..

[50] Pawitan Y. (2000). A reminder of the fallibility of the Wald statistic: Likelihood explanation. Am. Stat..

[51] López M.D., Contreras J., Pascual-Villalobos M.J. (2010). Selection for tolerance to volatile monoterpenoids in *Sitophilus oryzae* (L.), *Rhyzopertha dominica* (F.) and *Cryptolestes pusillus* (Schönherr). J. Stored Prod. Res..

[52] Lee B.H., Annis P.C., Tumaalii F., Choi W.S. (2004). Fumigant toxicity of essential oils from the Myrtaceae family and 1,8-cineole against 3 major stored-grain insects. J. Stored Prod. Res..

[53] Lee S., Peterson C.J., Coats J.R. (2003). Fumigation toxicity of monoterpenoids to several stored product insects. J. Stored Prod. Res..

