# Supplementary material for: Effects of Essential Oils from 24 Plant Species on Sitophilus zeamais Motsch (Coleoptera, Curculionidae)
Source: Insects. 2021 Jun 8;12(6):532. doi: 10.3390/insects12060532 (PMC8230103; doi:10.3390/insects12060532)
Supplement: Supplementary file 1 [file insects-12-00532-s001.zip › insects-1160839-s/EOs Chromatograms.pdf]

Chromatographic profiles obtained for the 14 active essential oils in columns DB-5MS (left) and HP-INNOWax (right)

Chromatographic profiles obtained for the essential oil of *X. discrete* fruits

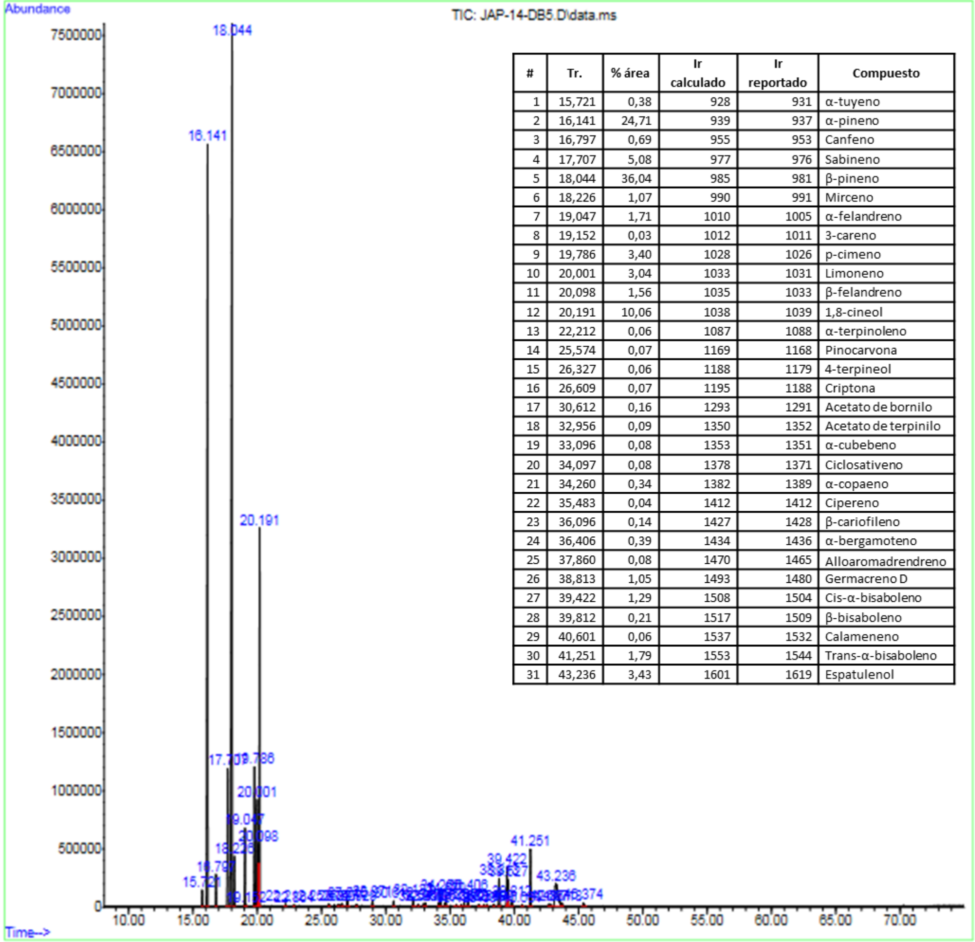

#	Tr.	% área	Ir calculado	Ir reportado	Compuesto
1	15,721	0,38	928	931	α-tuyeno
2	16,141	24,71	939	937	α-pineno
3	16,797	0,69	955	953	Canfeno
4	17,707	5,08	977	976	Sabineno
5	18,044	36,04	985	981	β-pineno
6	18,226	1,07	990	991	Mirceno
7	19,047	1,71	1010	1005	α-felandreno
8	19,152	0,03	1012	1011	3-careno
9	19,786	3,40	1028	1026	p-cimeno
10	20,001	3,04	1033	1031	Limoneno
11	20,098	1,56	1035	1033	β-felandreno
12	20,191	10,06	1038	1039	1,8-cineol
13	22,212	0,06	1087	1088	α-terpinoleno
14	25,574	0,07	1169	1168	Pinocarvona
15	26,327	0,06	1188	1179	4-terpineol
16	26,609	0,07	1195	1188	Criptona
17	30,612	0,16	1293	1291	Acetato de bornilo
18	32,956	0,09	1350	1352	Acetato de terpinilo
19	33,096	0,08	1353	1351	α-cubebeno
20	34,097	0,08	1378	1371	Ciclosativeno
21	34,260	0,34	1382	1389	α-copaeno
22	35,483	0,04	1412	1412	Cipereno
23	36,096	0,14	1427	1428	β-cariofileno
24	36,406	0,39	1434	1436	α-bergamoteno
25	37,860	0,08	1470	1465	Alloaromadrendreno
26	38,813	1,05	1493	1480	Germacreno D
27	39,422	1,29	1508	1504	Cis-α-bisaboleno
28	39,812	0,21	1517	1509	β-bisaboleno
29	40,601	0,06	1537	1532	Calameneno
30	41,251	1,79	1553	1544	Trans-α-bisaboleno
31	43,236	3,43	1601	1619	Espatulenol

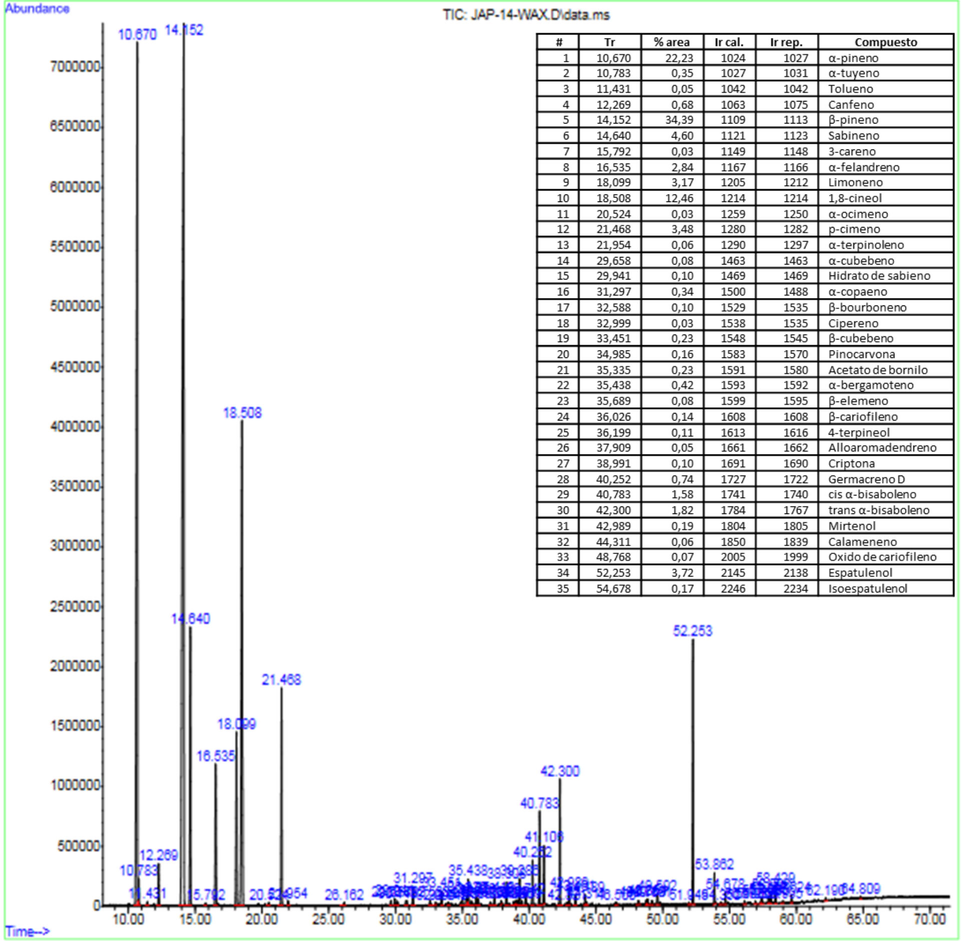

#	Tr	% area	Ir cal.	Ir rep.	Compuesto
1	10,670	22,23	1024	1027	α-pineno
2	10,783	0,35	1027	1031	α-tuyeno
3	11,431	0,05	1042	1042	Tolueno
4	12,269	0,68	1063	1075	Canfeno
5	14,152	34,39	1109	1113	β-pineno
6	14,640	4,60	1121	1123	Sabineno
7	15,792	0,03	1149	1148	3-careno
8	16,535	2,84	1167	1166	α-felandreno
9	18,099	3,17	1205	1212	Limoneno
10	18,508	12,46	1214	1214	1,8-cineol
11	20,524	0,03	1259	1250	α-ocimeno
12	21,468	3,48	1280	1282	p-cimeno
13	21,954	0,06	1290	1297	α-terpinoleno
14	29,658	0,08	1463	1463	α-cubebeno
15	29,941	0,10	1469	1469	Hidrato de sabieno
16	31,297	0,34	1500	1488	α-copaeno
17	32,588	0,10	1529	1535	β-bourboneno
18	32,999	0,03	1538	1535	Cipereno
19	33,451	0,23	1548	1545	β-cubebeno
20	34,985	0,16	1583	1570	Pinocarvona
21	35,335	0,23	1591	1580	Acetato de bornilo
22	35,438	0,42	1593	1592	α-bergamoteno
23	35,689	0,08	1599	1595	β-elemeno
24	36,026	0,14	1608	1608	β-cariofileno
25	36,199	0,11	1613	1616	4-terpineol
26	37,909	0,05	1661	1662	Alloaromadendreno
27	38,991	0,10	1691	1690	Criptona
28	40,252	0,74	1727	1722	Germacreno D
29	40,783	1,58	1741	1740	cis α-bisaboleno
30	42,300	1,82	1784	1767	trans α-bisaboleno
31	42,989	0,19	1804	1805	Mirtenol
32	44,311	0,06	1850	1839	Calameneno
33	48,768	0,07	2005	1999	Oxido de cariofileno
34	52,253	3,72	2145	2138	Espatulenol
35	54,678	0,17	2246	2234	Isoespatulenol

Chromatographic profiles obtained for the essential oil of *A. cumanensis* leaves and stems.

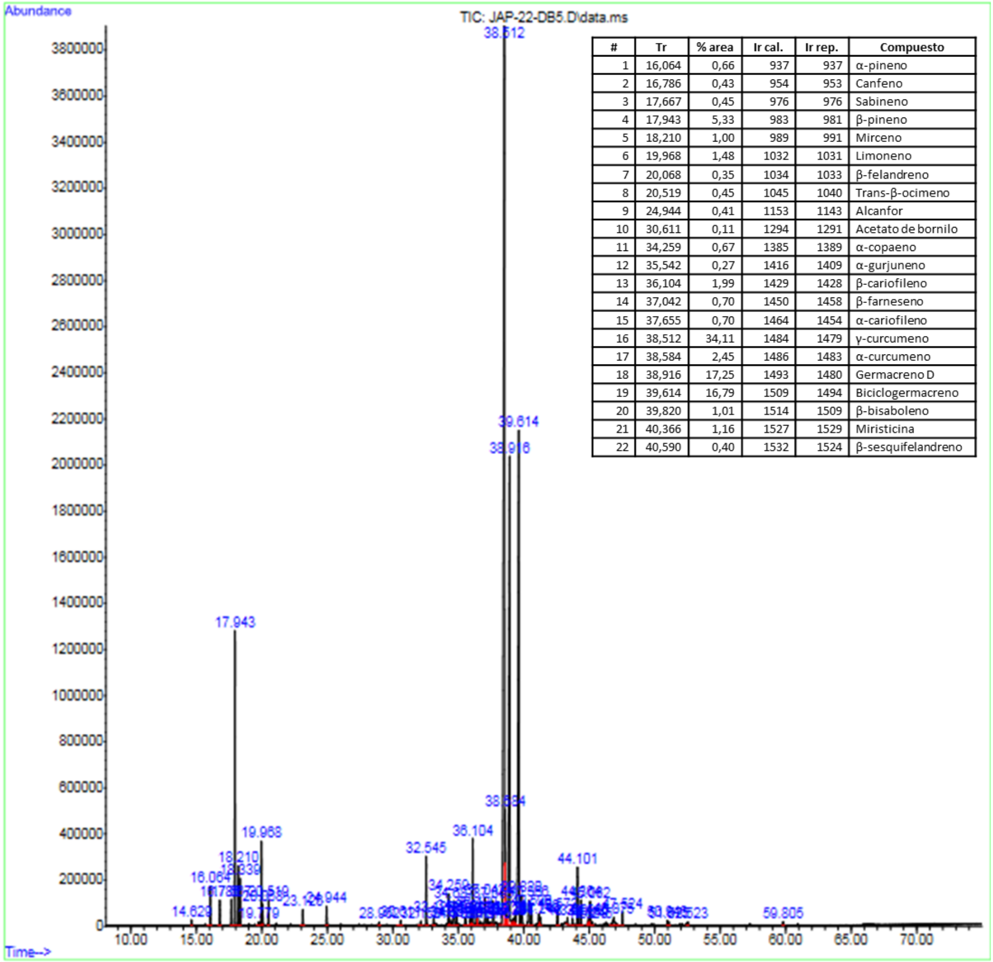

#	Tr	% area	Ir cal.	Ir rep.	Compuesto
1	16,064	0,66	937	937	α-pineno
2	16,786	0,43	954	953	Canfeno
3	17,667	0,45	976	976	Sabineno
4	17,943	5,33	983	981	β-pineno
5	18,210	1,00	989	991	Mirceno
6	19,968	1,48	1032	1031	Limoneno
7	20,068	0,35	1034	1033	β-felandreno
8	20,519	0,45	1045	1040	Trans-β-ocimeno
9	24,944	0,41	1153	1143	Alcanfor
10	30,611	0,11	1294	1291	Acetato de bornilo
11	34,259	0,67	1385	1389	α-copaeno
12	35,542	0,27	1416	1409	α-gurjuneno
13	36,104	1,99	1429	1428	β-cariofileno
14	37,042	0,70	1450	1458	β-farneseno
15	37,655	0,70	1464	1454	α-cariofileno
16	38,512	34,11	1484	1479	γ-curcumeno
17	38,584	2,45	1486	1483	α-curcumeno
18	38,916	17,25	1493	1480	Germacreno D
19	39,614	16,79	1509	1494	Biciclogermacreno
20	39,820	1,01	1514	1509	β-bisaboleno
21	40,366	1,16	1527	1529	Miristicina
22	40,590	0,40	1532	1524	β-sesquifelandreno

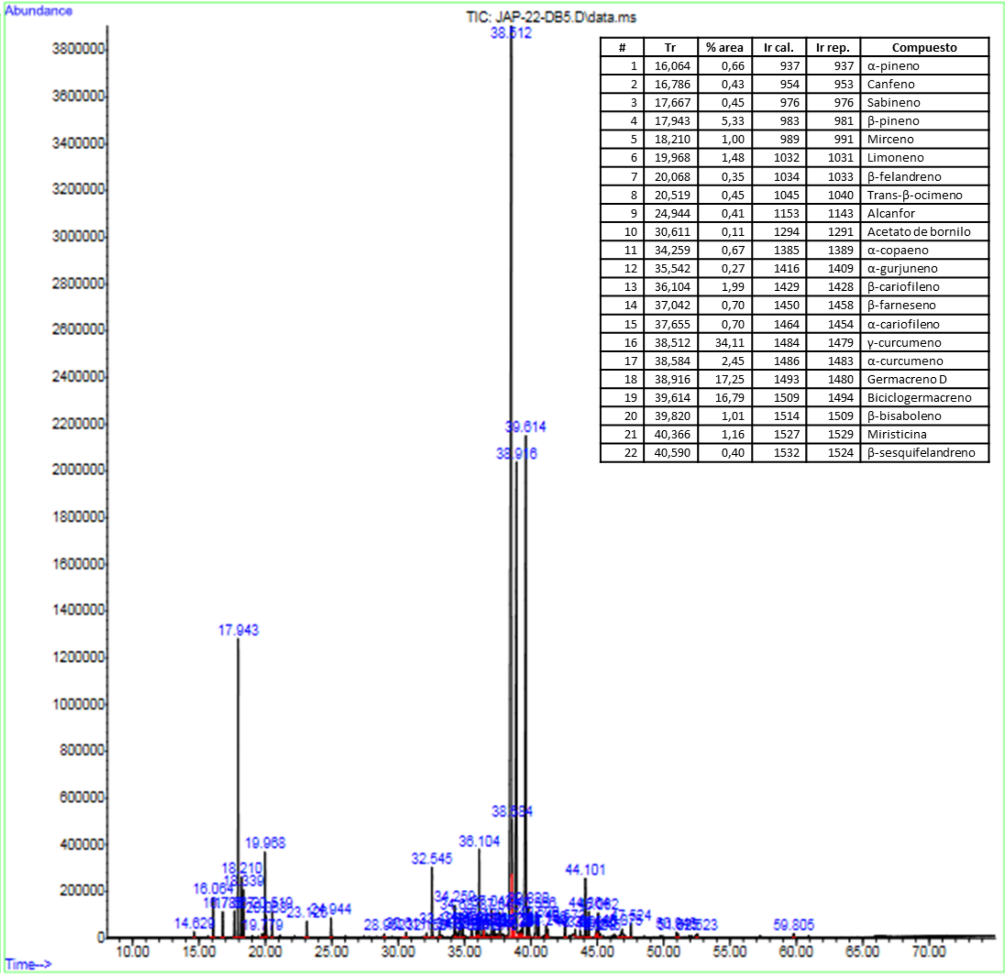

#	Tr	% area	Ir cal.	Ir rep.	Compuesto
1	16,064	0,66	937	937	α-pineno
2	16,786	0,43	954	953	Canfeno
3	17,667	0,45	976	976	Sabineno
4	17,943	5,33	983	981	β-pineno
5	18,210	1,00	989	991	Mirceno
6	19,968	1,48	1032	1031	Limoneno
7	20,068	0,35	1034	1033	β-felandreno
8	20,519	0,45	1045	1040	Trans-β-ocimeno
9	24,944	0,41	1153	1143	Alcanfor
10	30,611	0,11	1294	1291	Acetato de bornilo
11	34,259	0,67	1385	1389	α-copaeno
12	35,542	0,27	1416	1409	α-gurjuneno
13	36,104	1,99	1429	1428	β-cariofileno
14	37,042	0,70	1450	1458	β-farneseno
15	37,655	0,70	1464	1454	α-cariofileno
16	38,512	34,11	1484	1479	γ-curcumeno
17	38,584	2,45	1486	1483	α-curcumeno
18	38,916	17,25	1493	1480	Germacreno D
19	39,614	16,79	1509	1494	Biciclogermacreno
20	39,820	1,01	1514	1509	β-bisaboleno
21	40,366	1,16	1527	1529	Miristicina
22	40,590	0,40	1532	1524	β-sesquifelandreno

Chromatographic profiles obtained for the essential oil of *C. sempervirens* leaves.

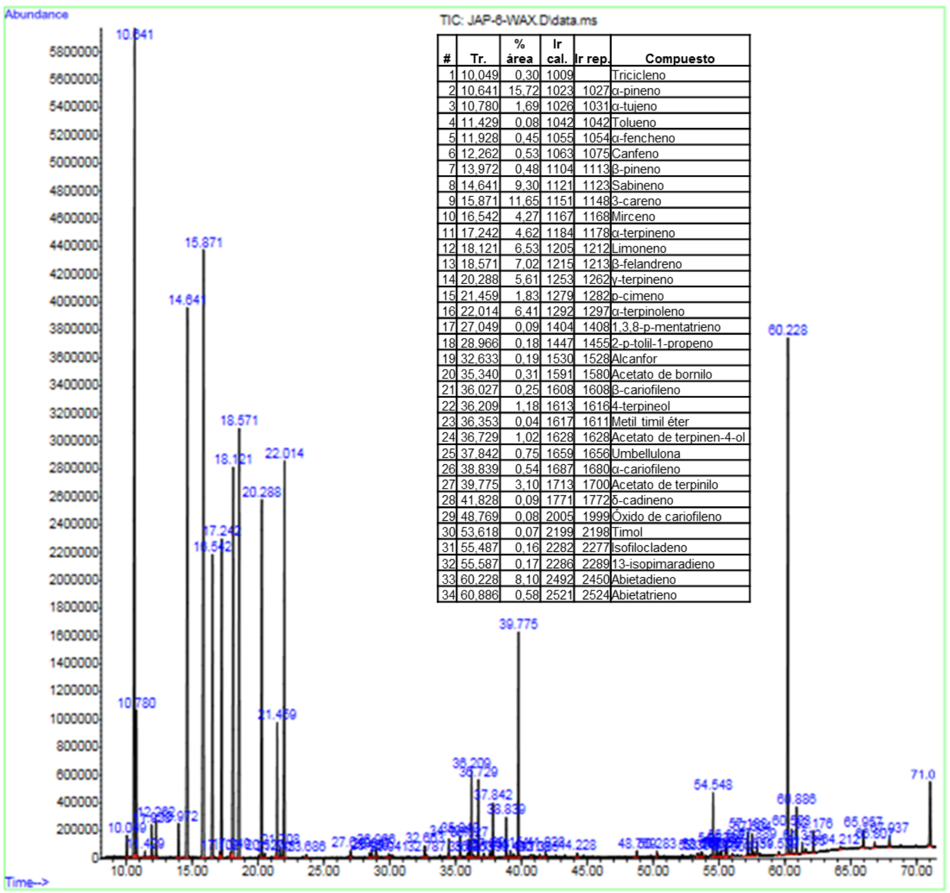

#	Tr.	% área	Ir cal.	Ir rep.	Compuesto
1	10,049	0,30	1009		Tricicleno
2	10,641	15,72	1023	1027	α-pineno
3	10,780	1,69	1026	1031	α-tujeno
4	11,429	0,08	1042	1042	Tolueno
5	11,928	0,45	1055	1054	α-fencheno
6	12,262	0,53	1063	1075	Canfeno
7	13,972	0,48	1104	1113	β-pineno
8	14,641	9,30	1121	1123	Sabineno
9	15,871	11,65	1151	1148	3-careno
10	16,542	4,27	1167	1168	Mirceno
11	17,242	4,62	1184	1178	α-terpineno
12	18,121	6,53	1205	1212	Limoneno
13	18,571	7,02	1215	1213	β-felandreno
14	20,288	5,61	1253	1262	γ-terpineno
15	21,459	1,83	1279	1282	p-cimeno
16	22,014	6,41	1292	1297	α-terpinoleno
17	27,049	0,09	1404	1408	1,3,8-p-mentatrieno
18	28,966	0,18	1447	1455	2-p-tolil-1-propeno
19	32,633	0,19	1530	1528	Alcanfor
20	35,340	0,31	1591	1580	Acetato de bornilo
21	36,027	0,25	1608	1608	β-cariofileno
22	36,209	1,18	1613	1616	4-terpineol
23	36,353	0,04	1617	1611	Metil timil éter
24	36,729	1,02	1628	1628	Acetato de terpinen-4-ol
25	37,842	0,75	1659	1656	Umbellulona
26	38,839	0,54	1687	1680	α-cariofileno
27	39,775	3,10	1713	1700	Acetato de terpinilo
28	41,828	0,09	1771	1772	δ-cadineno
29	48,769	0,08	2005	1999	Óxido de cariofileno
30	53,618	0,07	2199	2198	Timol
31	55,487	0,16	2282	2277	Isofilocladeno
32	55,587	0,17	2286	2289	13-isopimaradieno
33	60,228	8,10	2492	2450	Abietadieno
34	60,886	0,58	2521	2524	Abietatrieno

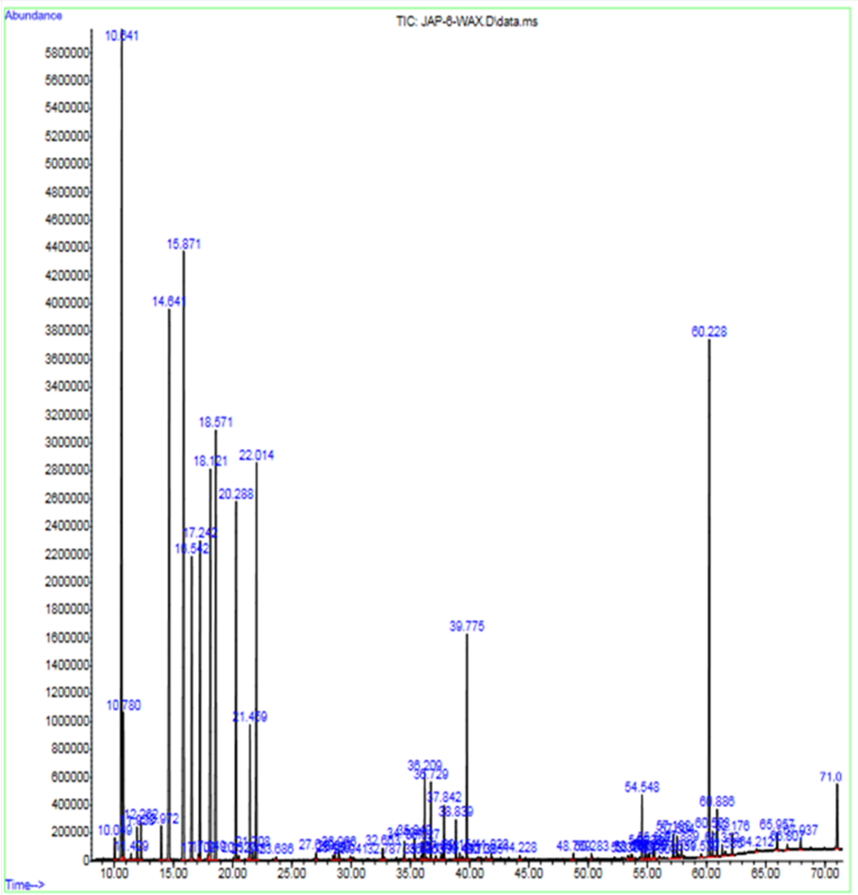

Chromatographic profiles obtained for the essential oil of *H.mexicanum* leaves and steams.

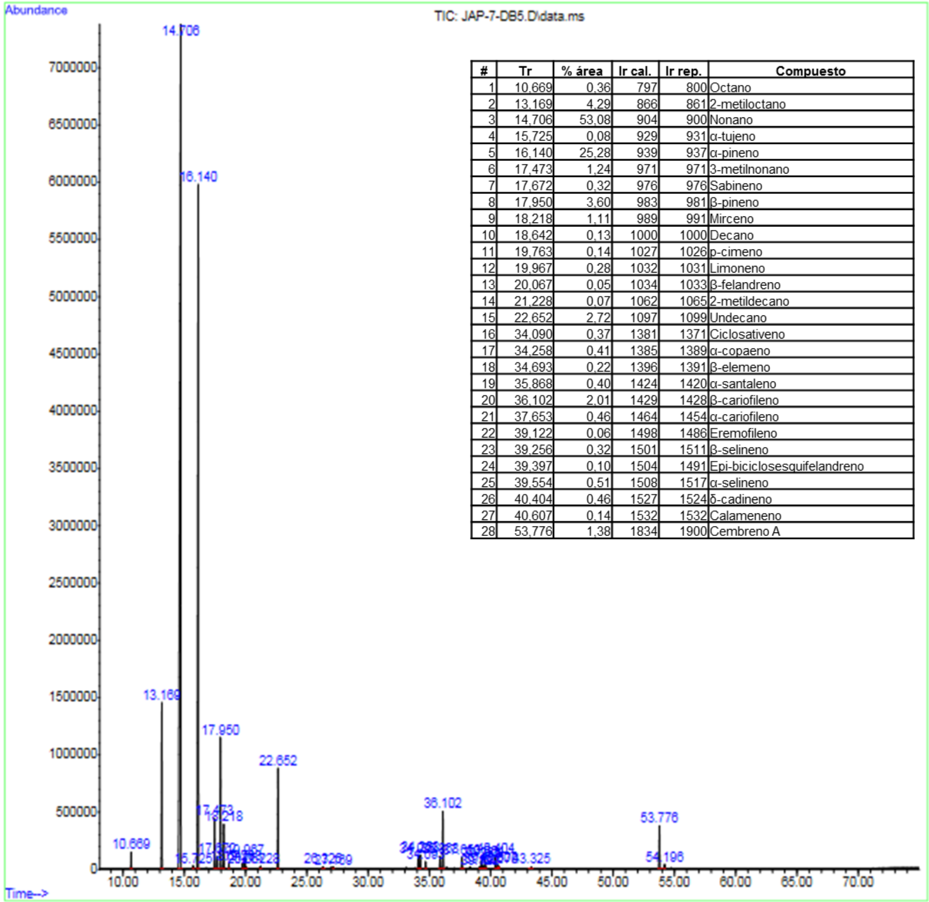

#	Tr	% área	Ir cal.	Ir rep.	Compuesto
1	10,669	0,36	797	800	Octano
2	13,169	4,29	866	861	2-metiloctano
3	14,706	53,08	904	900	Nonano
4	15,725	0,08	929	931	α-tujeno
5	16,140	25,28	939	937	α-pineno
6	17,473	1,24	971	971	3-metilnonano
7	17,672	0,32	976	976	Sabineno
8	17,950	3,60	983	981	β-pineno
9	18,218	1,11	989	991	Mirceno
10	18,642	0,13	1000	1000	Decano
11	19,763	0,14	1027	1026	p-cimeno
12	19,967	0,28	1032	1031	Limoneno
13	20,067	0,05	1034	1033	β-felandreno
14	21,228	0,07	1062	1065	2-metildecano
15	22,652	2,72	1097	1099	Undecano
16	34,090	0,37	1381	1371	Ciclosativeno
17	34,258	0,41	1385	1389	α-copaeno
18	34,693	0,22	1396	1391	β-elemeno
19	35,868	0,40	1424	1420	α-santaleno
20	36,102	2,01	1429	1428	β-cariofileno
21	37,653	0,46	1464	1454	α-cariofileno
22	39,122	0,06	1498	1486	Eremofileno
23	39,256	0,32	1501	1511	β-selineno
24	39,397	0,10	1504	1491	Epi-biciclosesquifelandreno
25	39,554	0,51	1508	1517	α-selineno
26	40,404	0,46	1527	1524	δ-cadineno
27	40,607	0,14	1532	1532	Calameneno
28	53,776	1,38	1834	1900	Cembreno A

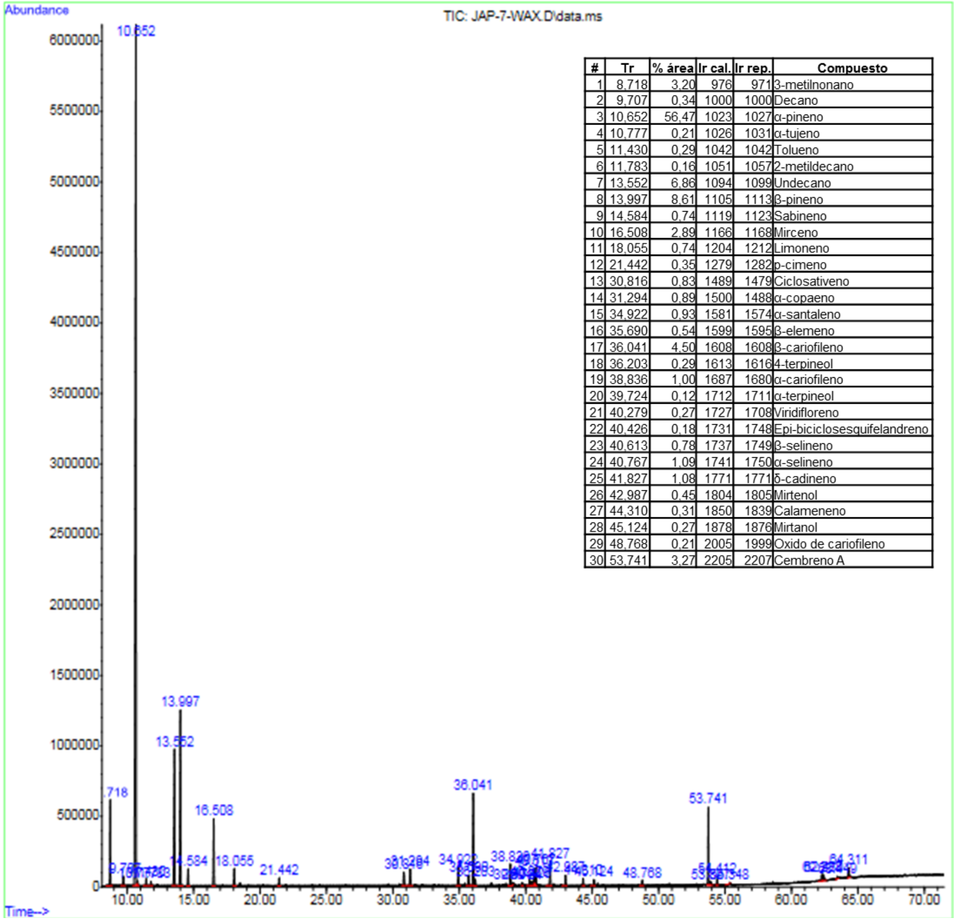

#	Tr	% área	Ir cal.	Ir rep.	Compuesto
1	8,718	3,20	976	971	3-metilnonano
2	9,707	0,34	1000	1000	Decano
3	10,652	56,47	1023	1027	α-pineno
4	10,777	0,21	1026	1031	α-tujeno
5	11,430	0,29	1042	1042	Tolueno
6	11,783	0,16	1051	1057	2-metildecano
7	13,552	6,86	1094	1099	Undecano
8	13,997	8,61	1105	1113	β-pineno
9	14,584	0,74	1119	1123	Sabineno
10	16,508	2,89	1166	1168	Mirceno
11	18,055	0,74	1204	1212	Limoneno
12	21,442	0,35	1279	1282	p-cimeno
13	30,816	0,83	1489	1479	Ciclosativeno
14	31,294	0,89	1500	1488	α-copaeno
15	34,922	0,93	1581	1574	α-santaleno
16	35,690	0,54	1599	1595	β-elemeno
17	36,041	4,50	1608	1608	β-cariofileno
18	36,203	0,29	1613	1616	4-terpineol
19	38,836	1,00	1687	1680	α-cariofileno
20	39,724	0,12	1712	1711	α-terpineol
21	40,279	0,27	1727	1708	Viridifloreno
22	40,426	0,18	1731	1748	Epi-biciclosesquifelandreno
23	40,613	0,78	1737	1749	β-selineno
24	40,767	1,09	1741	1750	α-selineno
25	41,827	1,08	1771	1771	δ-cadineno
26	42,987	0,45	1804	1805	Mirtenol
27	44,310	0,31	1850	1839	Calameneno
28	45,124	0,27	1878	1876	Mirtanol
29	48,768	0,21	2005	1999	Oxido de cariofileno
30	53,741	3,27	2205	2207	Cembreno A

Chromatographic profiles obtained for the essential oil of *H. myricariifolium* leaves and steams.

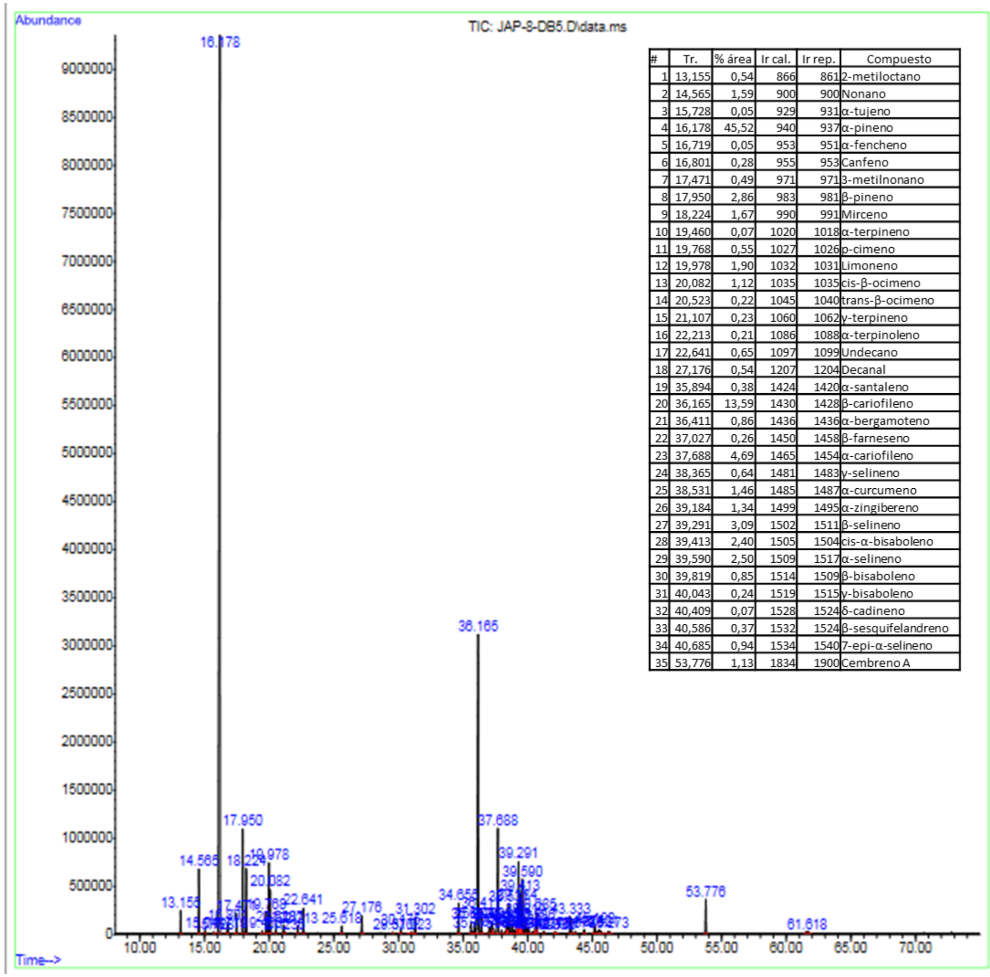

#	Tr.	% área	Ir cal.	Ir rep.	Compuesto
1	13,155	0,54	866	861	2-metiloctano
2	14,565	1,59	900	900	Nonano
3	15,728	0,05	929	931	α-tujeno
4	16,178	45,52	940	937	α-pineno
5	16,719	0,05	953	951	α-fencheno
6	16,801	0,28	955	953	Canfeno
7	17,471	0,49	971	971	3-metilnonano
8	17,950	2,86	983	981	β-pineno
9	18,224	1,67	990	991	Mirceno
10	19,460	0,07	1020	1018	α-terpineno
11	19,768	0,55	1027	1026	p-cimeno
12	19,978	1,90	1032	1031	Limoneno
13	20,082	1,12	1035	1035	cis-β-ocimeno
14	20,523	0,22	1045	1040	trans-β-ocimeno
15	21,107	0,23	1060	1062	γ-terpineno
16	22,213	0,21	1086	1088	α-terpinoleno
17	22,641	0,65	1097	1099	Undecano
18	27,176	0,54	1207	1204	Decanal
19	35,894	0,38	1424	1420	α-santaleno
20	36,165	13,59	1430	1428	β-cariofileno
21	36,411	0,86	1436	1436	α-bergamoteno
22	37,027	0,26	1450	1458	β-farneseno
23	37,688	4,69	1465	1454	α-cariofileno
24	38,365	0,64	1481	1483	γ-selineno
25	38,531	1,46	1485	1487	α-curcumeno
26	39,184	1,34	1499	1495	α-zingibereno
27	39,291	3,09	1502	1511	β-selineno
28	39,413	2,40	1505	1504	cis-α-bisaboleno
29	39,590	2,50	1509	1517	α-selineno
30	39,819	0,85	1514	1509	β-bisaboleno
31	40,043	0,24	1519	1515	γ-bisaboleno
32	40,409	0,07	1528	1524	δ-cadineno
33	40,586	0,37	1532	1524	β-sesquifelandreno
34	40,685	0,94	1534	1540	7-epi-α-selineno
35	53,776	1,13	1834	1900	Cembreno A

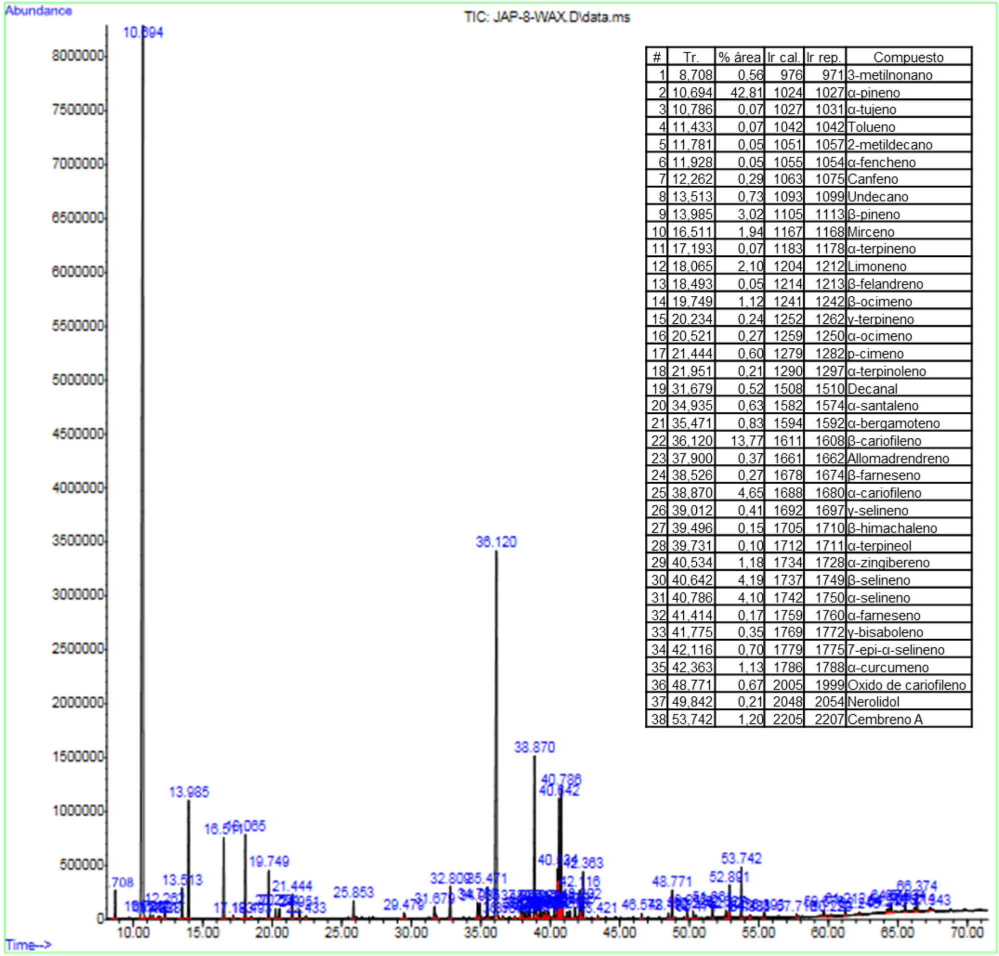

#	Tr.	% área	Ir cal.	Ir rep.	Compuesto
1	8,708	0,56	976	971	3-metilnonano
2	10,694	42,81	1024	1027	α-pineno
3	10,786	0,07	1027	1031	α-tujeno
4	11,433	0,07	1042	1042	Tolueno
5	11,781	0,05	1051	1057	2-metildecano
6	11,928	0,05	1055	1054	α-fencheno
7	12,262	0,29	1063	1075	Canfeno
8	13,513	0,73	1093	1099	Undecano
9	13,985	3,02	1105	1113	β-pineno
10	16,511	1,94	1167	1168	Mirceno
11	17,193	0,07	1183	1178	α-terpineno
12	18,065	2,10	1204	1212	Limoneno
13	18,493	0,05	1214	1213	β-felandreno
14	19,749	1,12	1241	1242	β-ocimeno
15	20,234	0,24	1252	1262	γ-terpineno
16	20,521	0,27	1259	1250	α-ocimeno
17	21,444	0,60	1279	1282	p-cimeno
18	21,951	0,21	1290	1297	α-terpinoleno
19	31,679	0,52	1508	1510	Decanal
20	34,935	0,63	1582	1574	α-santaleno
21	35,471	0,83	1594	1592	α-bergamoteno
22	36,120	13,77	1611	1608	β-cariofileno
23	37,900	0,37	1661	1662	Allomadrendreno
24	38,526	0,27	1678	1674	β-farneseno
25	38,870	4,65	1688	1680	α-cariofileno
26	39,012	0,41	1692	1697	γ-selineno
27	39,496	0,15	1705	1710	β-himachaleno
28	39,731	0,10	1712	1711	α-terpineol
29	40,534	1,18	1734	1728	α-zingibereno
30	40,642	4,19	1737	1749	β-selineno
31	40,786	4,10	1742	1750	α-selineno
32	41,414	0,17	1759	1760	α-farneseno
33	41,775	0,35	1769	1772	γ-bisaboleno
34	42,116	0,70	1779	1775	7-epi-α-selineno
35	42,363	1,13	1786	1788	α-curcumeno
36	48,771	0,67	2005	1999	Oxido de cariofileno
37	49,842	0,21	2048	2054	Nerolidol
38	53,742	1,20	2205	2207	Cembreno A

Chromatographic profiles obtained for the essential oil of *I. verum* fruits.

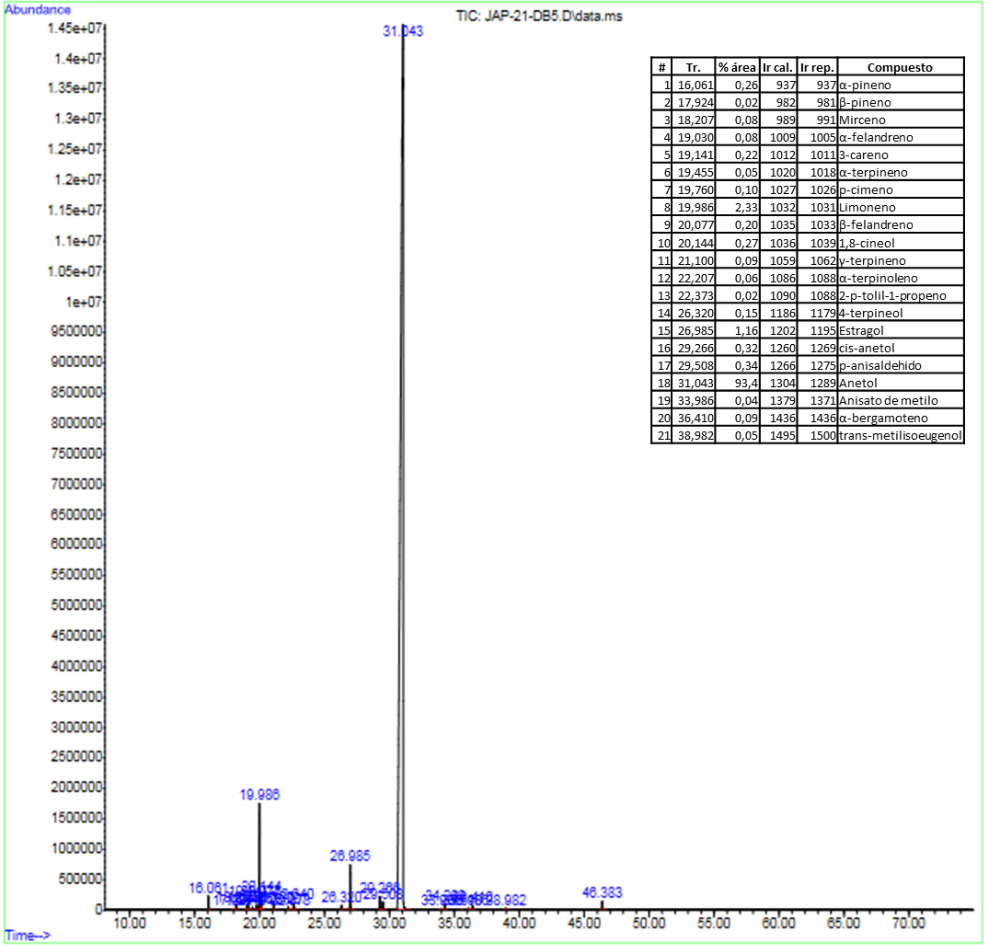

#	Tr.	% área	Ir cal.	Ir rep.	Compuesto
1	16,061	0,26	937	937	α-pineno
2	17,924	0,02	982	981	β-pineno
3	18,207	0,08	989	991	Mirceno
4	19,030	0,08	1009	1005	α-felandreno
5	19,141	0,22	1012	1011	3-careno
6	19,455	0,05	1020	1018	α-terpineno
7	19,760	0,10	1027	1026	p-cimeno
8	19,986	2,33	1032	1031	Limoneno
9	20,077	0,20	1035	1033	β-felandreno
10	20,144	0,27	1036	1039	1,8-cineol
11	21,100	0,09	1059	1062	γ-terpineno
12	22,207	0,06	1086	1088	α-terpinoleno
13	22,373	0,02	1090	1088	2-p-tolil-1-propeno
14	26,320	0,15	1186	1179	4-terpineol
15	26,985	1,16	1202	1195	Estragol
16	29,266	0,32	1260	1269	cis-anetol
17	29,508	0,34	1266	1275	p-anisaldehido
18	31,043	93,4	1304	1289	Anetol
19	33,986	0,04	1379	1371	Anisato de metilo
20	36,410	0,09	1436	1436	α-bergamoteno
21	38,982	0,05	1495	1500	trans-metilisoeugenol

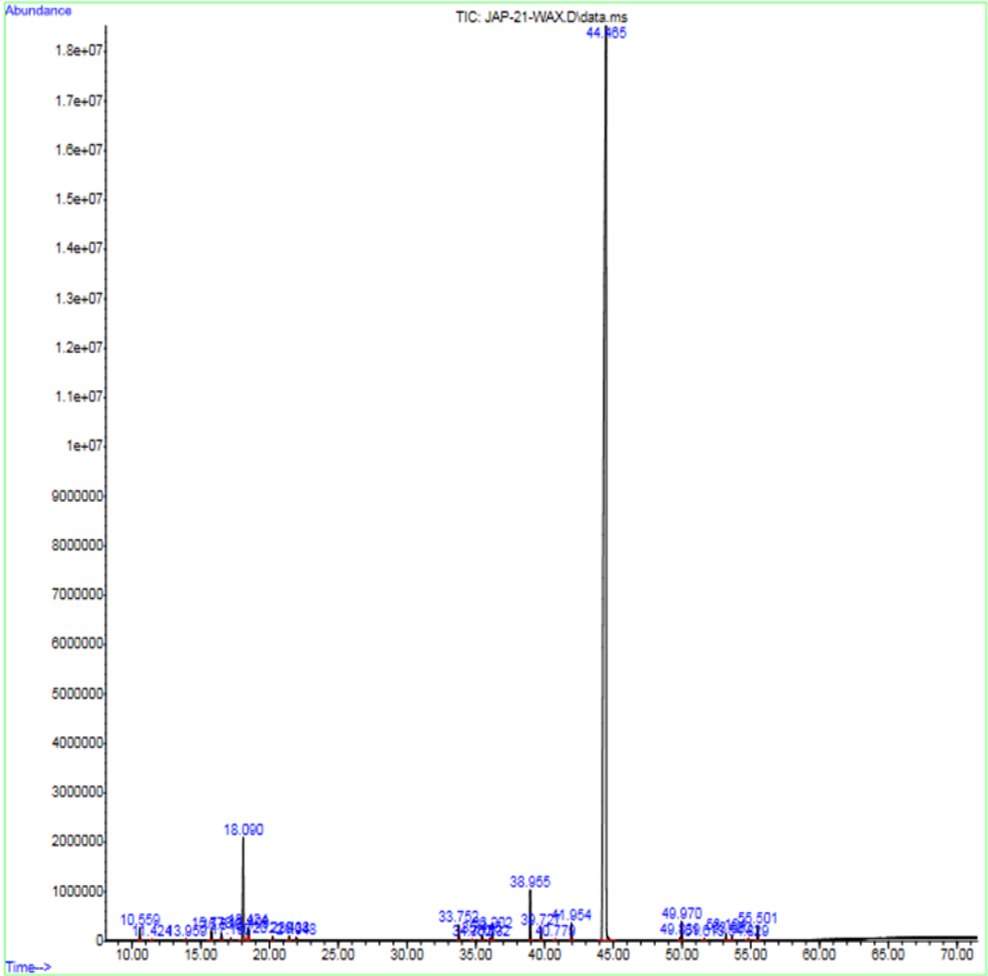

Chromatographic profiles obtained for the essential oil of *R. officinalis* leaves and steams.

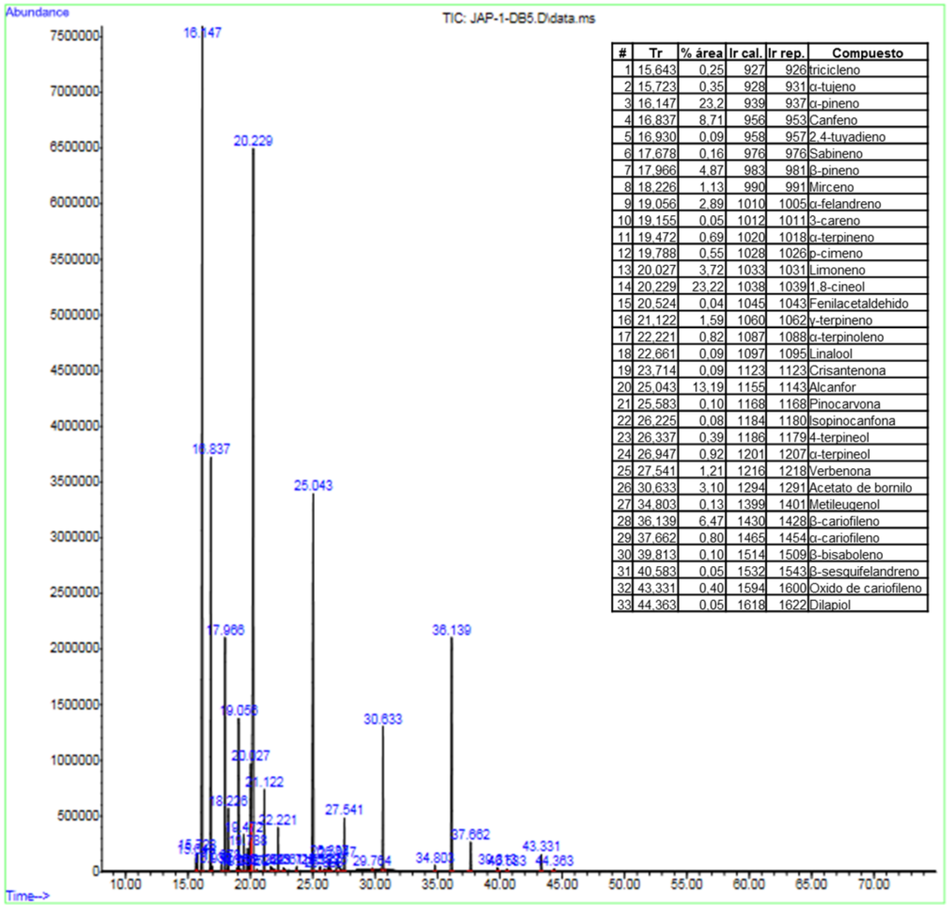

#	Tr	% área	Ir cal.	Ir rep.	Compuesto
1	15,643	0,25	927	926	tricicleno
2	15,723	0,35	928	931	α-tujeno
3	16,147	23,2	939	937	α-pineno
4	16,837	8,71	956	953	Canfeno
5	16,930	0,09	958	957	2,4-tuyadieno
6	17,678	0,16	976	976	Sabineno
7	17,966	4,87	983	981	β-pineno
8	18,226	1,13	990	991	Mirceno
9	19,056	2,89	1010	1005	α-felandreno
10	19,155	0,05	1012	1011	3-careno
11	19,472	0,69	1020	1018	α-terpineno
12	19,788	0,55	1028	1026	p-cimeno
13	20,027	3,72	1033	1031	Limoneno
14	20,229	23,22	1038	1039	1,8-cineol
15	20,524	0,04	1045	1043	Fenilacetaldehido
16	21,122	1,59	1060	1062	γ-terpineno
17	22,221	0,82	1087	1088	α-terpinoleno
18	22,661	0,09	1097	1095	Linalool
19	23,714	0,09	1123	1123	Crisantenona
20	25,043	13,19	1155	1143	Alcanfor
21	25,583	0,10	1168	1168	Pinocarvona
22	26,225	0,08	1184	1180	Isopinocanfona
23	26,337	0,39	1186	1179	4-terpineol
24	26,947	0,92	1201	1207	α-terpineol
25	27,541	1,21	1216	1218	Verbenona
26	30,633	3,10	1294	1291	Acetato de bornilo
27	34,803	0,13	1399	1401	Metileugenol
28	36,139	6,47	1430	1428	β-cariofileno
29	37,662	0,80	1465	1454	α-cariofileno
30	39,813	0,10	1514	1509	β-bisaboleno
31	40,583	0,05	1532	1543	β-sesquifelandreno
32	43,331	0,40	1594	1600	Oxido de cariofileno
33	44,363	0,05	1618	1622	Dilapiol

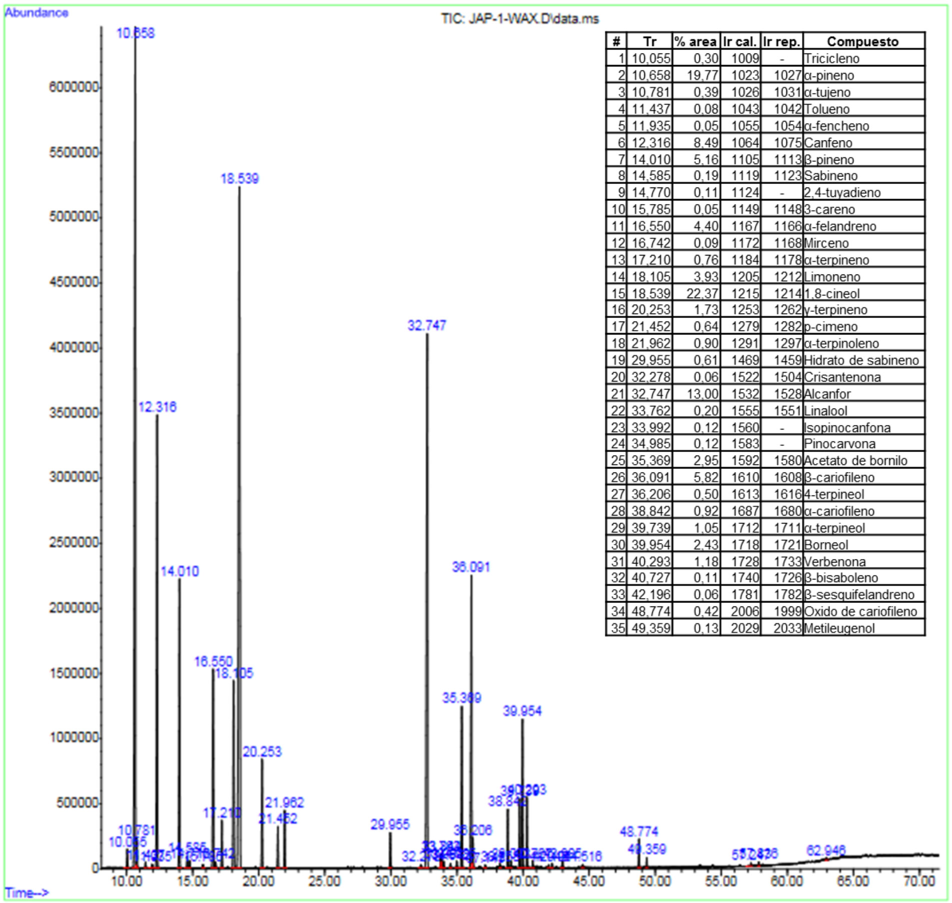

#	Tr	% area	Ir cal.	Ir rep.	Compuesto
1	10,055	0,30	1009	-	Tricicleno
2	10,658	19,77	1023	1027	α-pineno
3	10,781	0,39	1026	1031	α-tujeno
4	11,437	0,08	1043	1042	Tolueno
5	11,935	0,05	1055	1054	α-fencheno
6	12,316	8,49	1064	1075	Canfeno
7	14,010	5,16	1105	1113	β-pineno
8	14,585	0,19	1119	1123	Sabineno
9	14,770	0,11	1124	-	2,4-tuyadieno
10	15,785	0,05	1149	1148	3-careno
11	16,550	4,40	1167	1166	α-felandreno
12	16,742	0,09	1172	1168	Mirceno
13	17,210	0,76	1184	1178	α-terpineno
14	18,105	3,93	1205	1212	Limoneno
15	18,539	22,37	1215	1214	1,8-cineol
16	20,253	1,73	1253	1262	γ-terpineno
17	21,452	0,64	1279	1282	p-cimeno
18	21,962	0,90	1291	1297	α-terpinoleno
19	29,955	0,61	1469	1459	Hidrato de sabineno
20	32,278	0,06	1522	1504	Crisantenona
21	32,747	13,00	1532	1528	Alcanfor
22	33,762	0,20	1555	1551	Linalool
23	33,992	0,12	1560	-	Isopinocanfona
24	34,985	0,12	1583	-	Pinocarvona
25	35,369	2,95	1592	1580	Acetato de bornilo
26	36,091	5,82	1610	1608	β-cariofileno
27	36,206	0,50	1613	1616	4-terpineol
28	38,842	0,92	1687	1680	α-cariofileno
29	39,739	1,05	1712	1711	α-terpineol
30	39,954	2,43	1718	1721	Borneol
31	40,293	1,18	1728	1733	Verbenona
32	40,727	0,11	1740	1726	β-bisaboleno
33	42,196	0,06	1781	1782	β-sesquifelandreno
34	48,774	0,42	2006	1999	Oxido de cariofileno
35	49,359	0,13	2029	2033	Metileugenol

Chromatographic profiles obtained for the essential oil of *L. stoechas* leaves and flowers.

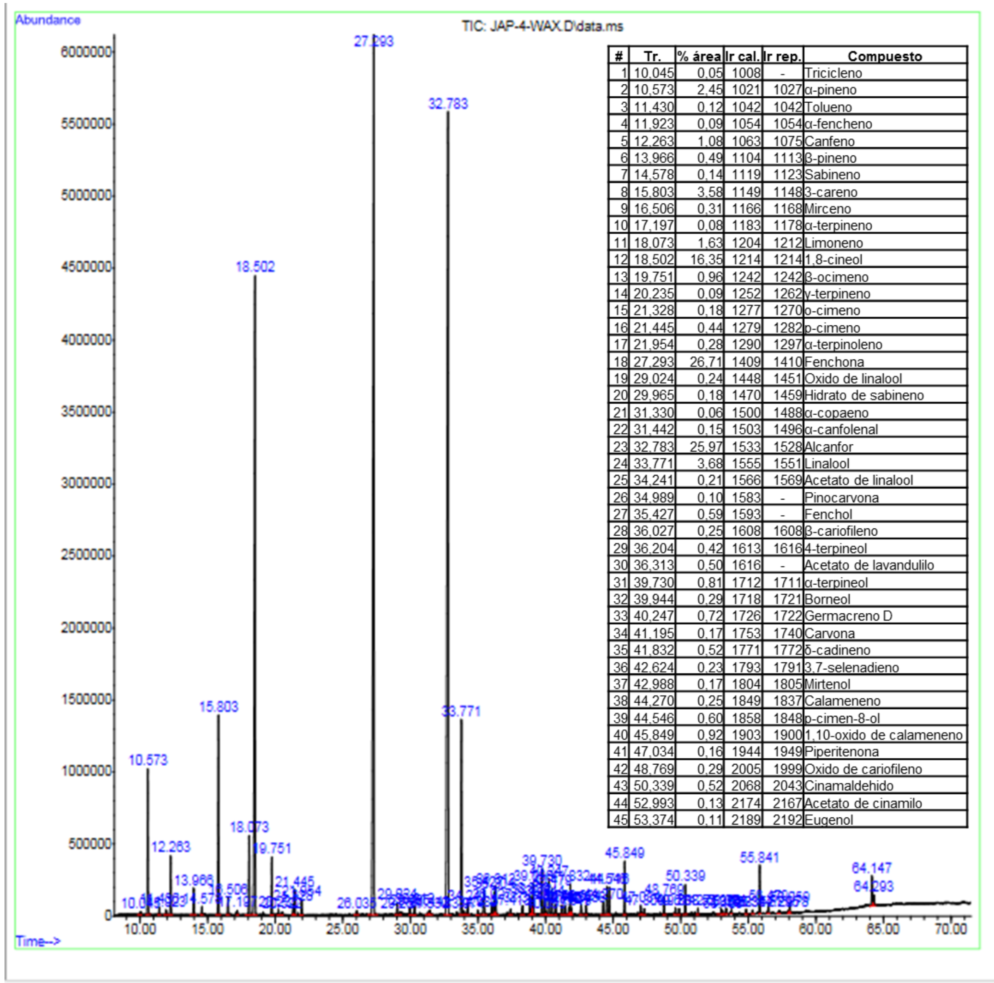

#	Tr.	% área	Ir cal.	Ir rep.	Compuesto
1	10,045	0,05	1008	-	Tricicleno
2	10,573	2,45	1021	1027	α-pineno
3	11,430	0,12	1042	1042	Tolueno
4	11,923	0,09	1054	1054	α-fencheno
5	12,263	1,08	1063	1075	Canfeno
6	13,966	0,49	1104	1113	β-pineno
7	14,578	0,14	1119	1123	Sabineno
8	15,803	3,58	1149	1148	3-careno
9	16,506	0,31	1166	1168	Mirceno
10	17,197	0,08	1183	1178	α-terpineno
11	18,073	1,63	1204	1212	Limoneno
12	18,502	16,35	1214	1214	1,8-cineol
13	19,751	0,96	1242	1242	β-ocimeno
14	20,235	0,09	1252	1262	γ-terpineno
15	21,328	0,18	1277	1270	o-cimeno
16	21,445	0,44	1279	1282	p-cimeno
17	21,954	0,28	1290	1297	α-terpinoleno
18	27,293	26,71	1409	1410	Fenchona
19	29,024	0,24	1448	1451	Oxido de linalool
20	29,965	0,18	1470	1459	Hidrato de sabineno
21	31,330	0,06	1500	1488	α-copaeno
22	31,442	0,15	1503	1496	α-canfolenal
23	32,783	25,97	1533	1528	Alcanfor
24	33,771	3,68	1555	1551	Linalool
25	34,241	0,21	1566	1569	Acetato de linalool
26	34,989	0,10	1583	-	Pinocarvona
27	35,427	0,59	1593	-	Fenchol
28	36,027	0,25	1608	1608	β-cariofileno
29	36,204	0,42	1613	1616	4-terpineol
30	36,313	0,50	1616	-	Acetato de lavandulilo
31	39,730	0,81	1712	1711	α-terpineol
32	39,944	0,29	1718	1721	Borneol
33	40,247	0,72	1726	1722	Germacreno D
34	41,195	0,17	1753	1740	Carvona
35	41,832	0,52	1771	1772	δ-cadineno
36	42,624	0,23	1793	1791	3,7-selenadieno
37	42,988	0,17	1804	1805	Mirtenol
38	44,270	0,25	1849	1837	Calameneno
39	44,546	0,60	1858	1848	p-cimen-8-ol
40	45,849	0,92	1903	1900	1,10-oxido de calameneno
41	47,034	0,16	1944	1949	Piperitenona
42	48,769	0,29	2005	1999	Oxido de cariofileno
43	50,339	0,52	2068	2043	Cinamaldehido
44	52,993	0,13	2174	2167	Acetato de cinamilo
45	53,374	0,11	2189	2192	Eugenol

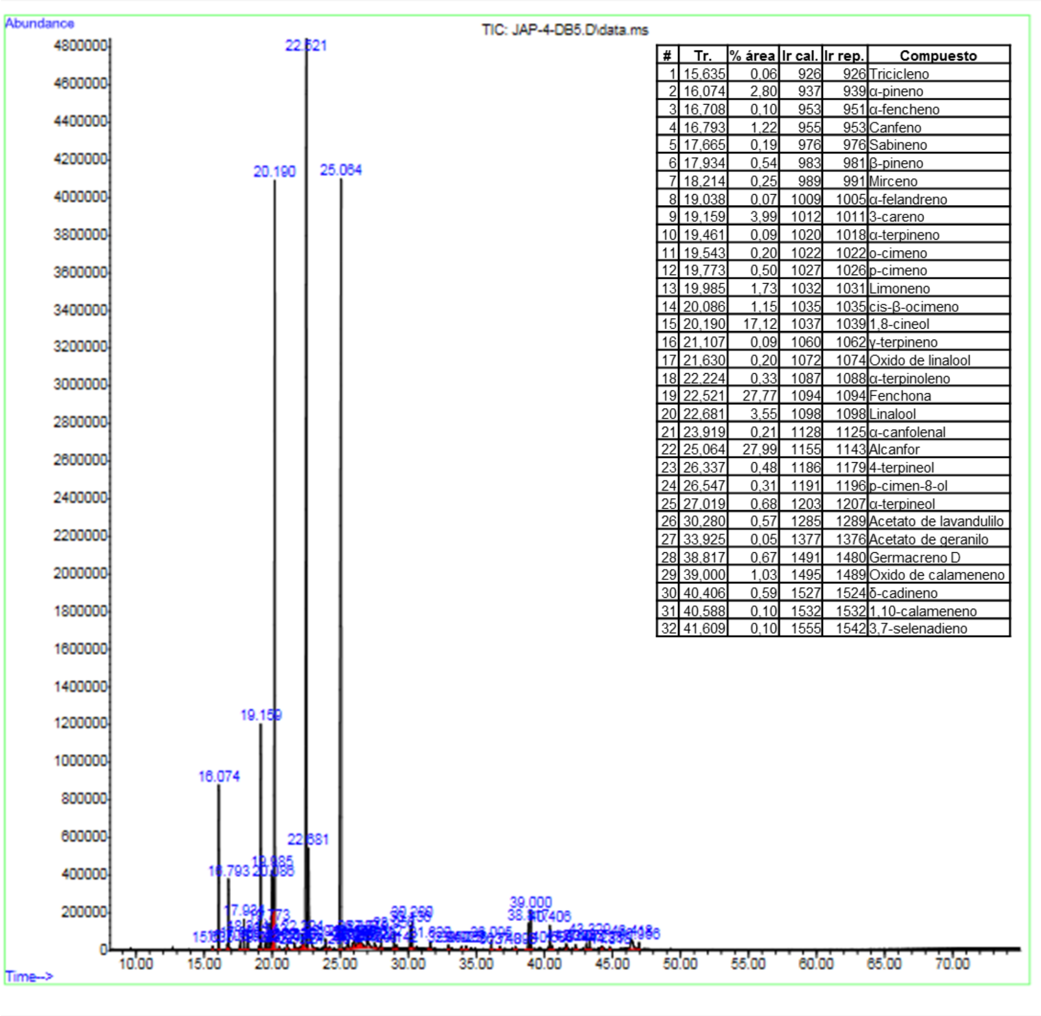

#	Tr.	% área	Ir cal.	Ir rep.	Compuesto
1	15,635	0,06	926	926	Tricicleno
2	16,074	2,80	937	939	α-pineno
3	16,708	0,10	953	951	α-fencheno
4	16,793	1,22	955	953	Canfeno
5	17,665	0,19	976	976	Sabineno
6	17,934	0,54	983	981	β-pineno
7	18,214	0,25	989	991	Mirceno
8	19,038	0,07	1009	1005	α-felandreno
9	19,159	3,99	1012	1011	3-careno
10	19,461	0,09	1020	1018	α-terpineno
11	19,543	0,20	1022	1022	o-cimeno
12	19,773	0,50	1027	1026	p-cimeno
13	19,985	1,73	1032	1031	Limoneno
14	20,086	1,15	1035	1035	cis-β-ocimeno
15	20,190	17,12	1037	1039	1,8-cineol
16	21,107	0,09	1060	1062	γ-terpineno
17	21,630	0,20	1072	1074	Oxido de linalool
18	22,224	0,33	1087	1088	α-terpinoleno
19	22,521	27,77	1094	1094	Fenchona
20	22,681	3,55	1098	1098	Linalool
21	23,919	0,21	1128	1125	α-canfolenal
22	25,064	27,99	1155	1143	Alcanfor
23	26,337	0,48	1186	1179	4-terpineol
24	26,547	0,31	1191	1196	p-cimen-8-ol
25	27,019	0,68	1203	1207	α-terpineol
26	30,280	0,57	1285	1289	Acetato de lavandulilo
27	33,925	0,05	1377	1376	Acetato de geranilo
28	38,817	0,67	1491	1480	Germacreno D
29	39,000	1,03	1495	1489	Oxido de calameneno
30	40,406	0,59	1527	1524	δ-cadineno
31	40,588	0,10	1532	1532	1,10-calameneno
32	41,609	0,10	1555	1542	3,7-selenadieno

Chromatographic profiles obtained for the essential oil of *S. viminea* leaves and steams.

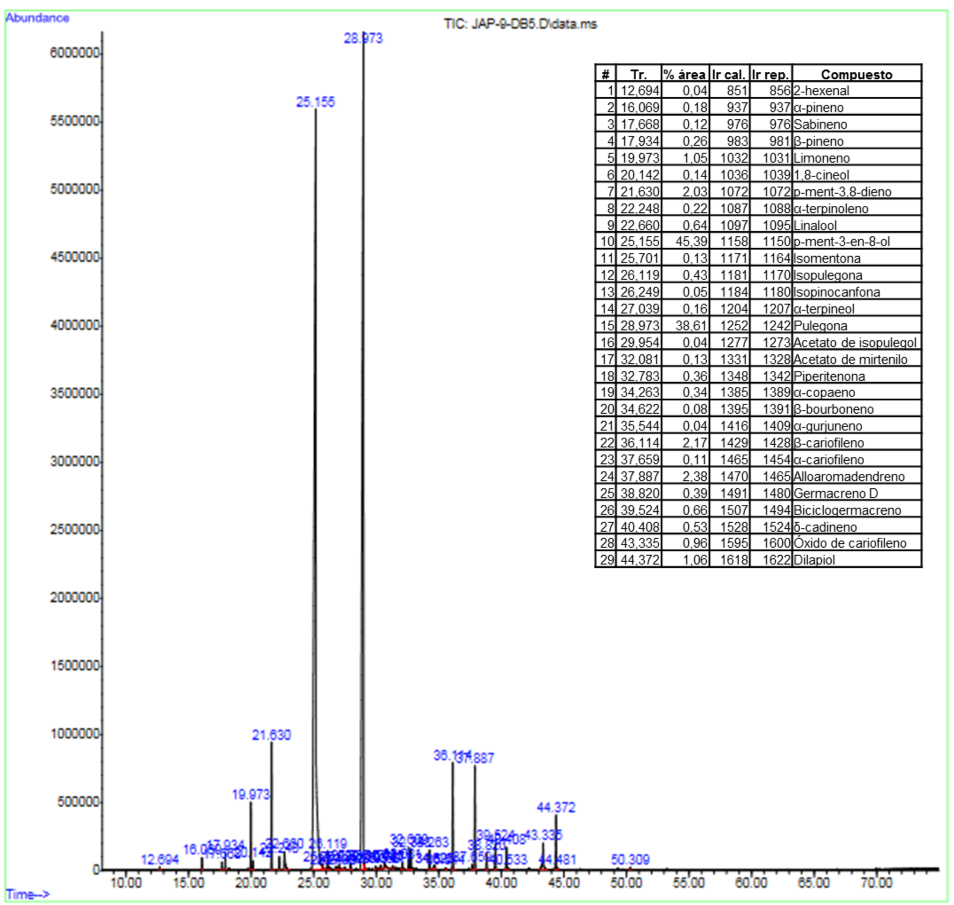

#	Tr.	% área	Ir cal.	Ir rep.	Compuesto
1	12,694	0,04	851	856	2-hexenal
2	16,069	0,18	937	937	α-pineno
3	17,668	0,12	976	976	Sabineno
4	17,934	0,26	983	981	β-pineno
5	19,973	1,05	1032	1031	Limoneno
6	20,142	0,14	1036	1039	1,8-cineol
7	21,630	2,03	1072	1072	p-ment-3,8-dieno
8	22,248	0,22	1087	1088	α-terpinoleno
9	22,660	0,64	1097	1095	Linalool
10	25,155	45,39	1158	1150	p-ment-3-en-8-ol
11	25,701	0,13	1171	1164	Isomentona
12	26,119	0,43	1181	1170	Isopulegona
13	26,249	0,05	1184	1180	Isopinocanfona
14	27,039	0,16	1204	1207	α-terpineol
15	28,973	38,61	1252	1242	Pulegona
16	29,954	0,04	1277	1273	Acetato de isopulegol
17	32,081	0,13	1331	1328	Acetato de mirtenilo
18	32,783	0,36	1348	1342	Piperitenona
19	34,263	0,34	1385	1389	α-copaeno
20	34,622	0,08	1395	1391	β-bourboneno
21	35,544	0,04	1416	1409	α-gurjuneno
22	36,114	2,17	1429	1428	β-cariofileno
23	37,659	0,11	1465	1454	α-cariofileno
24	37,887	2,38	1470	1465	Alloaromadendreno
25	38,820	0,39	1491	1480	Germacreno D
26	39,524	0,66	1507	1494	Biciclogermacreno
27	40,408	0,53	1528	1524	δ-cadineno
28	43,335	0,96	1595	1600	Óxido de cariofileno
29	44,372	1,06	1618	1622	Dilapiol

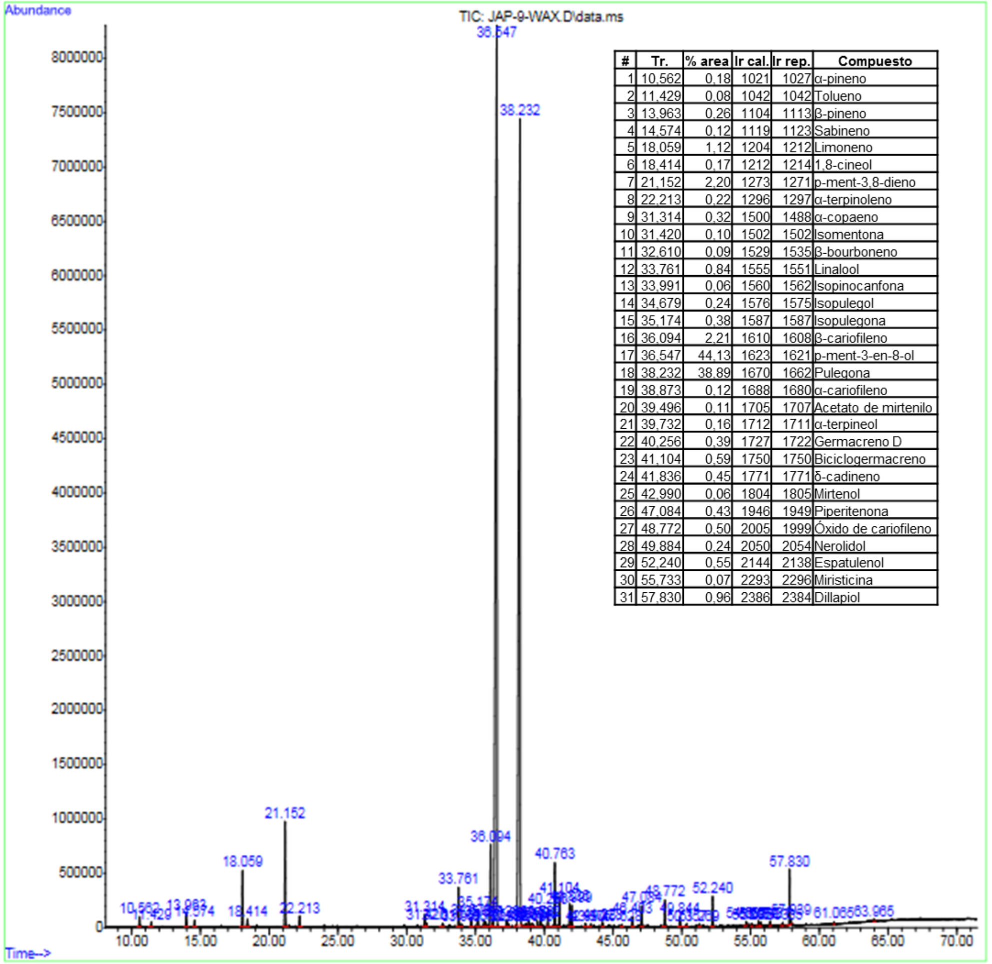

#	Tr.	% area	Ir cal.	Ir rep.	Compuesto
1	10,562	0,18	1021	1027	α-pineno
2	11,429	0,08	1042	1042	Tolueno
3	13,963	0,26	1104	1113	β-pineno
4	14,574	0,12	1119	1123	Sabineno
5	18,059	1,12	1204	1212	Limoneno
6	18,414	0,17	1212	1214	1,8-cineol
7	21,152	2,20	1273	1271	p-ment-3,8-dieno
8	22,213	0,22	1296	1297	α-terpinoleno
9	31,314	0,32	1500	1488	α-copaeno
10	31,420	0,10	1502	1502	Isomentona
11	32,610	0,09	1529	1535	β-bourboneno
12	33,761	0,84	1555	1551	Linalool
13	33,991	0,06	1560	1562	Isopinocanfona
14	34,679	0,24	1576	1575	Isopulegol
15	35,174	0,38	1587	1587	Isopulegona
16	36,094	2,21	1610	1608	β-cariofileno
17	36,547	44,13	1623	1621	p-ment-3-en-8-ol
18	38,232	38,89	1670	1662	Pulegona
19	38,873	0,12	1688	1680	α-cariofileno
20	39,496	0,11	1705	1707	Acetato de mirtenilo
21	39,732	0,16	1712	1711	α-terpineol
22	40,256	0,39	1727	1722	Germacreno D
23	41,104	0,59	1750	1750	Biciclogermacreno
24	41,836	0,45	1771	1771	δ-cadineno
25	42,990	0,06	1804	1805	Mirtenol
26	47,084	0,43	1946	1949	Piperitenona
27	48,772	0,50	2005	1999	Óxido de cariofileno
28	49,884	0,24	2050	2054	Nerolidol
29	52,240	0,55	2144	2138	Espatulenol
30	55,733	0,07	2293	2296	Miristicina
31	57,830	0,96	2386	2384	Dillapiol

Chromatographic profiles obtained for the essential oil of *M. septentrionalis* leaves and steams.

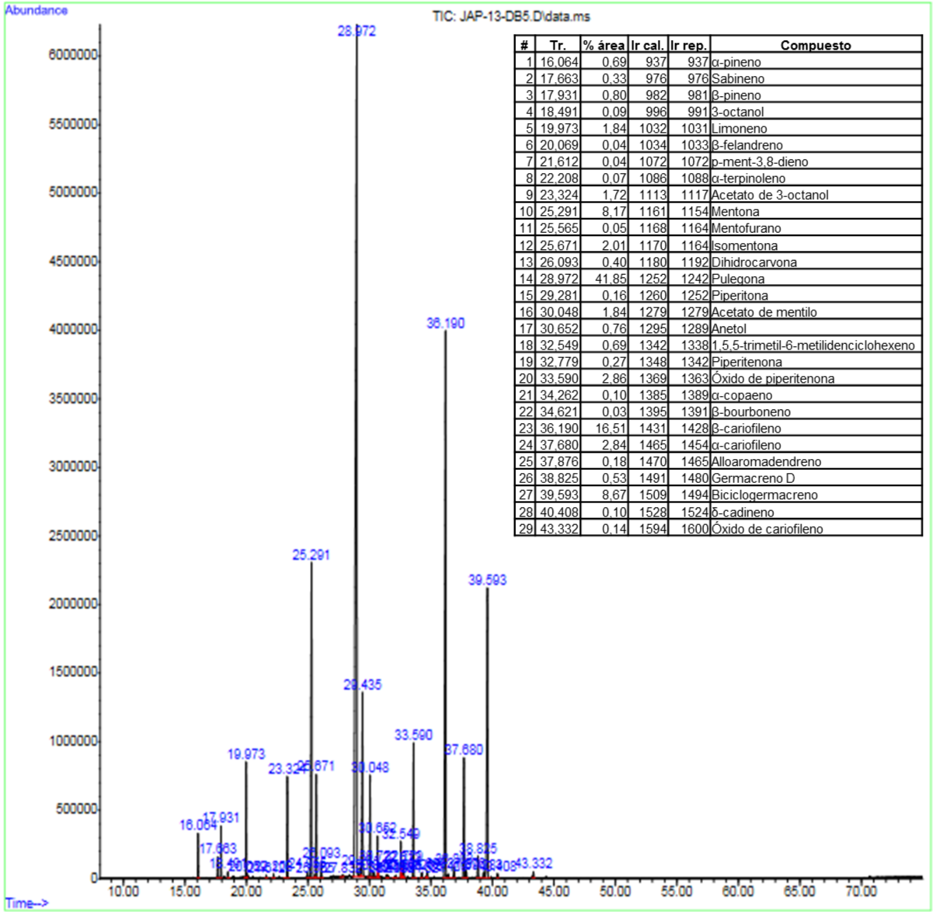

#	Tr.	% área	Ir cal.	Ir rep.	Compuesto
1	16,064	0,69	937	937	α-pineno
2	17,663	0,33	976	976	Sabineno
3	17,931	0,80	982	981	β-pineno
4	18,491	0,09	996	991	3-octanol
5	19,973	1,84	1032	1031	Limoneno
6	20,069	0,04	1034	1033	β-felandreno
7	21,612	0,04	1072	1072	p-ment-3,8-dieno
8	22,208	0,07	1086	1088	α-terpinoleno
9	23,324	1,72	1113	1117	Acetato de 3-octanol
10	25,291	8,17	1161	1154	Mentona
11	25,565	0,05	1168	1164	Mentofurano
12	25,671	2,01	1170	1164	Isomentona
13	26,093	0,40	1180	1192	Dihidrocarvona
14	28,972	41,85	1252	1242	Pulegona
15	29,281	0,16	1260	1252	Piperitona
16	30,048	1,84	1279	1279	Acetato de mentilo
17	30,652	0,76	1295	1289	Anetol
18	32,549	0,69	1342	1338	1,5,5-trimetil-6-metilidenciclohexeno
19	32,779	0,27	1348	1342	Piperitenona
20	33,590	2,86	1369	1363	Óxido de piperitenona
21	34,262	0,10	1385	1389	α-copaeno
22	34,621	0,03	1395	1391	β-bourboneno
23	36,190	16,51	1431	1428	β-cariofileno
24	37,680	2,84	1465	1454	α-cariofileno
25	37,876	0,18	1470	1465	Alloaromadendreno
26	38,825	0,53	1491	1480	Germacreno D
27	39,593	8,67	1509	1494	Biciclogermacreno
28	40,408	0,10	1528	1524	δ-cadineno
29	43,332	0,14	1594	1600	Óxido de cariofileno

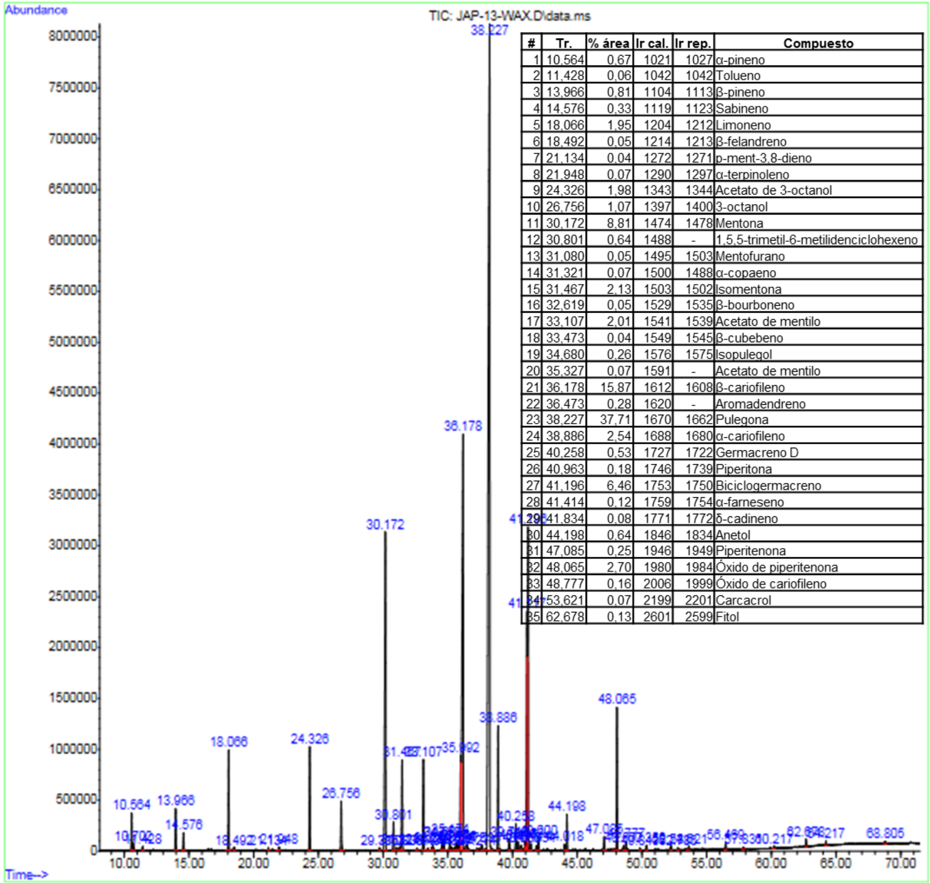

#	Tr.	% área	Ir cal.	Ir rep.	Compuesto
1	10,564	0,67	1021	1027	α-pineno
2	11,428	0,06	1042	1042	Tolueno
3	13,966	0,81	1104	1113	β-pineno
4	14,576	0,33	1119	1123	Sabineno
5	18,066	1,95	1204	1212	Limoneno
6	18,492	0,05	1214	1213	β-felandreno
7	21,134	0,04	1272	1271	p-ment-3,8-dieno
8	21,948	0,07	1290	1297	α-terpinoleno
9	24,326	1,98	1343	1344	Acetato de 3-octanol
10	26,756	1,07	1397	1400	3-octanol
11	30,172	8,81	1474	1478	Mentona
12	30,801	0,64	1488	-	1,5,5-trimetil-6-metilidenciclohexeno
13	31,080	0,05	1495	1503	Mentofurano
14	31,321	0,07	1500	1488	α-copaeno
15	31,467	2,13	1503	1502	Isomentona
16	32,619	0,05	1529	1535	β-bourboneno
17	33,107	2,01	1541	1539	Acetato de mentilo
18	33,473	0,04	1549	1545	β-cubebeno
19	34,680	0,26	1576	1575	Isopulegol
20	35,327	0,07	1591	-	Acetato de mentilo
21	36,178	15,87	1612	1608	β-cariofileno
22	36,473	0,28	1620	-	Aromadendreno
23	38,227	37,71	1670	1662	Pulegona
24	38,886	2,54	1688	1680	α-cariofileno
25	40,258	0,53	1727	1722	Germacreno D
26	40,963	0,18	1746	1739	Piperitona
27	41,196	6,46	1753	1750	Biciclogermacreno
28	41,414	0,12	1759	1754	α-farneseno
29	41,834	0,08	1771	1772	δ-cadineno
30	44,198	0,64	1846	1834	Anetol
31	47,085	0,25	1946	1949	Piperitenona
32	48,065	2,70	1980	1984	Óxido de piperitenona
33	48,777	0,16	2006	1999	Óxido de cariofileno
34	53,621	0,07	2199	2201	Carcacrol
35	62,678	0,13	2601	2599	Fitol

Chromatographic profiles obtained for the essential oil of *O. basilicum* leaves.

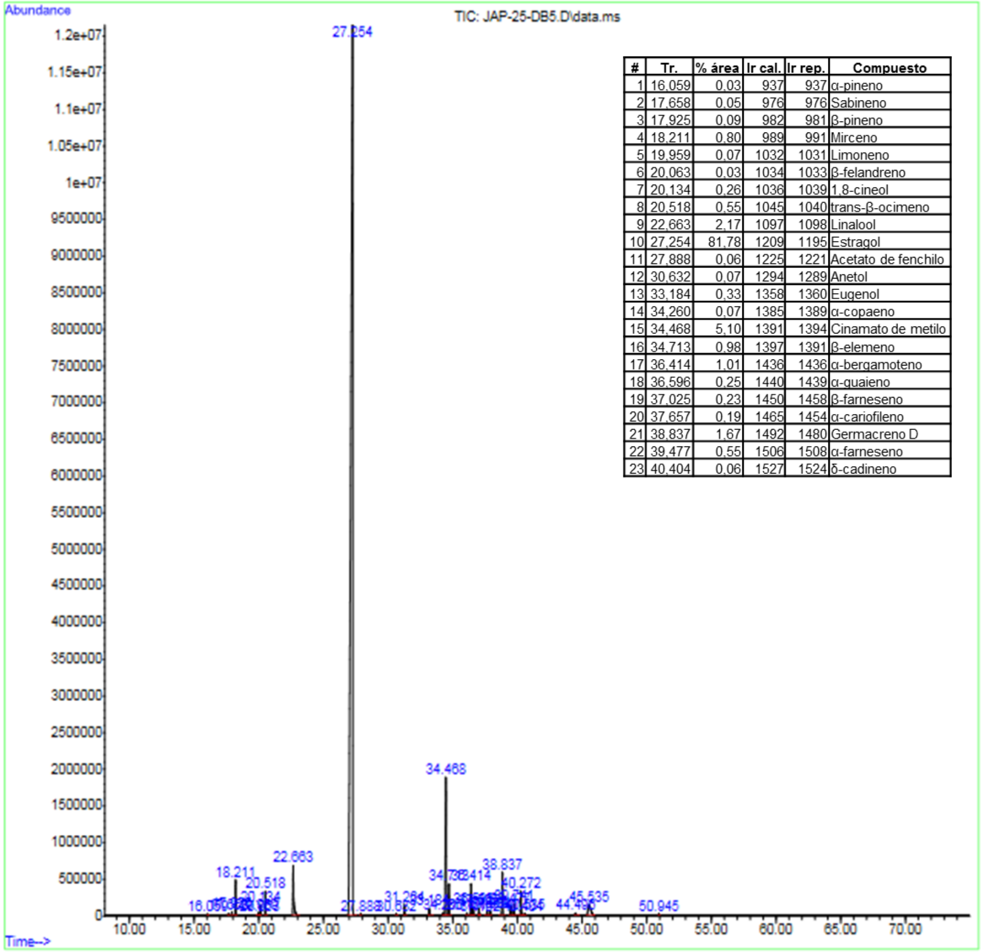

#	Tr.	% área	Ir cal.	Ir rep.	Compuesto
1	16,059	0,03	937	937	α-pineno
2	17,658	0,05	976	976	Sabineno
3	17,925	0,09	982	981	β-pineno
4	18,211	0,80	989	991	Mirceno
5	19,959	0,07	1032	1031	Limoneno
6	20,063	0,03	1034	1033	β-felandreno
7	20,134	0,26	1036	1039	1,8-cineol
8	20,518	0,55	1045	1040	trans-β-ocimeno
9	22,663	2,17	1097	1098	Linalool
10	27,254	81,78	1209	1195	Estragol
11	27,888	0,06	1225	1221	Acetato de fenchilo
12	30,632	0,07	1294	1289	Anetol
13	33,184	0,33	1358	1360	Eugenol
14	34,260	0,07	1385	1389	α-copaeno
15	34,468	5,10	1391	1394	Cinamato de metilo
16	34,713	0,98	1397	1391	β-elemeno
17	36,414	1,01	1436	1436	α-bergamoteno
18	36,596	0,25	1440	1439	α-guaieno
19	37,025	0,23	1450	1458	β-farneseno
20	37,657	0,19	1465	1454	α-cariofileno
21	38,837	1,67	1492	1480	Germacreno D
22	39,477	0,55	1506	1508	α-farneseno
23	40,404	0,06	1527	1524	δ-cadineno

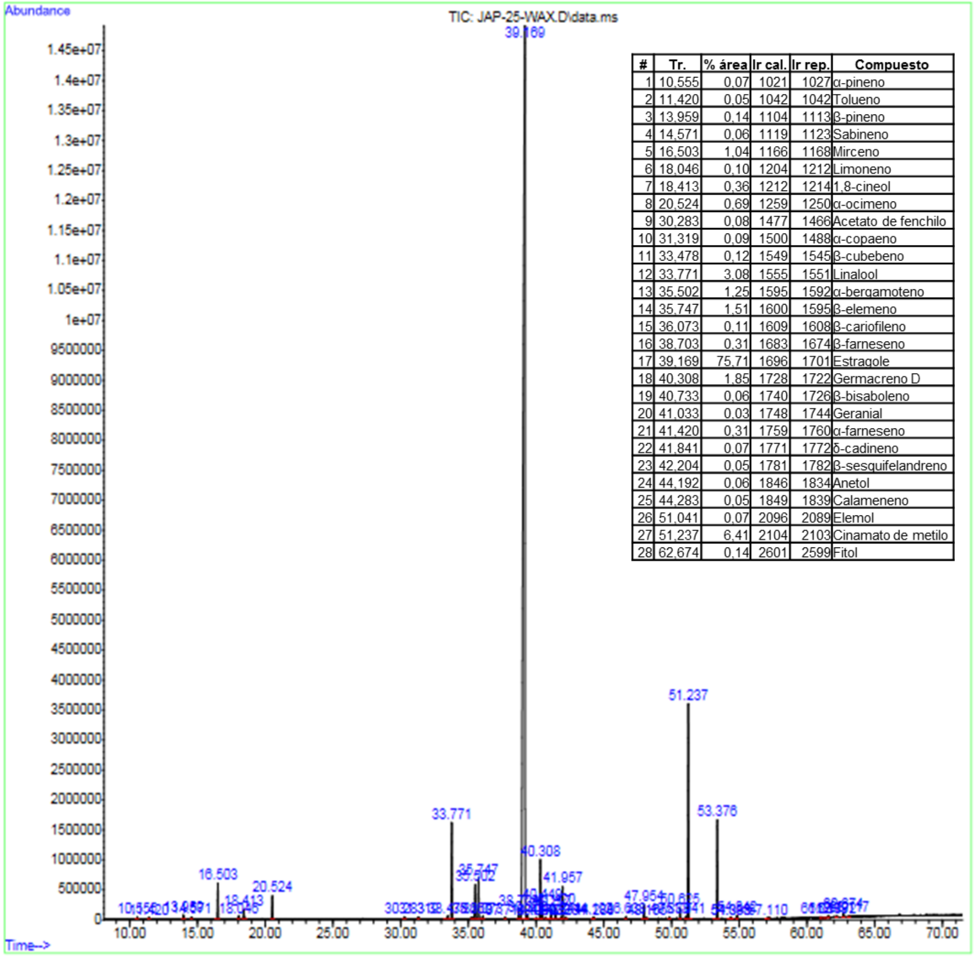

#	Tr.	% área	Ir cal.	Ir rep.	Compuesto
1	10,555	0,07	1021	1027	α-pineno
2	11,420	0,05	1042	1042	Tolueno
3	13,959	0,14	1104	1113	β-pineno
4	14,571	0,06	1119	1123	Sabineno
5	16,503	1,04	1166	1168	Mirceno
6	18,046	0,10	1204	1212	Limoneno
7	18,413	0,36	1212	1214	1,8-cineol
8	20,524	0,69	1259	1250	α-ocimeno
9	30,283	0,08	1477	1466	Acetato de fenchilo
10	31,319	0,09	1500	1488	α-copaeno
11	33,478	0,12	1549	1545	β-cubebeno
12	33,771	3,08	1555	1551	Linalool
13	35,502	1,25	1595	1592	α-bergamoteno
14	35,747	1,51	1600	1595	β-elemeno
15	36,073	0,11	1609	1608	β-cariofileno
16	38,703	0,31	1683	1674	β-farneseno
17	39,169	75,71	1696	1701	Estragole
18	40,308	1,85	1728	1722	Germacreno D
19	40,733	0,06	1740	1726	β-bisaboleno
20	41,033	0,03	1748	1744	Geranial
21	41,420	0,31	1759	1760	α-farneseno
22	41,841	0,07	1771	1772	δ-cadineno
23	42,204	0,05	1781	1782	β-sesquifelandreno
24	44,192	0,06	1846	1834	Anetol
25	44,283	0,05	1849	1839	Calameneno
26	51,041	0,07	2096	2089	Elemol
27	51,237	6,41	2104	2103	Cinamato de metilo
28	62,674	0,14	2601	2599	Fitol

Chromatographic profiles obtained for the essential oil of *Ocotea* sp.

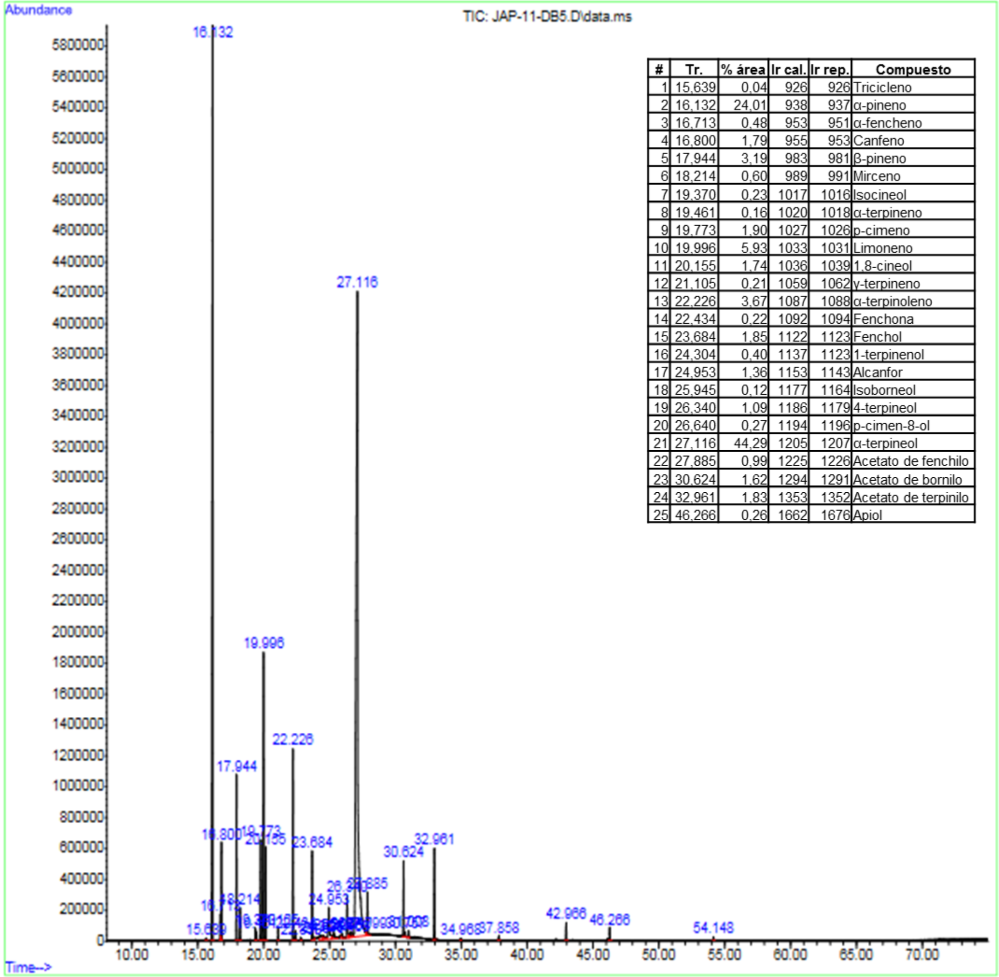

#	Tr.	% área	Ir cal.	Ir rep.	Compuesto
1	15,639	0,04	926	926	Tricicleno
2	16,132	24,01	938	937	α-pineno
3	16,713	0,48	953	951	α-fencheno
4	16,800	1,79	955	953	Canfeno
5	17,944	3,19	983	981	β-pineno
6	18,214	0,60	989	991	Mirceno
7	19,370	0,23	1017	1016	Isocineol
8	19,461	0,16	1020	1018	α-terpineno
9	19,773	1,90	1027	1026	p-cimeno
10	19,996	5,93	1033	1031	Limoneno
11	20,155	1,74	1036	1039	1,8-cineol
12	21,105	0,21	1059	1062	γ-terpineno
13	22,226	3,67	1087	1088	α-terpinoleno
14	22,434	0,22	1092	1094	Fenchona
15	23,684	1,85	1122	1123	Fenchol
16	24,304	0,40	1137	1123	1-terpinenol
17	24,953	1,36	1153	1143	Alcanfor
18	25,945	0,12	1177	1164	Isoborneol
19	26,340	1,09	1186	1179	4-terpineol
20	26,640	0,27	1194	1196	p-cimen-8-ol
21	27,116	44,29	1205	1207	α-terpineol
22	27,885	0,99	1225	1226	Acetato de fenchilo
23	30,624	1,62	1294	1291	Acetato de bornilo
24	32,961	1,83	1353	1352	Acetato de terpinilo
25	46,266	0,26	1662	1676	Apiol

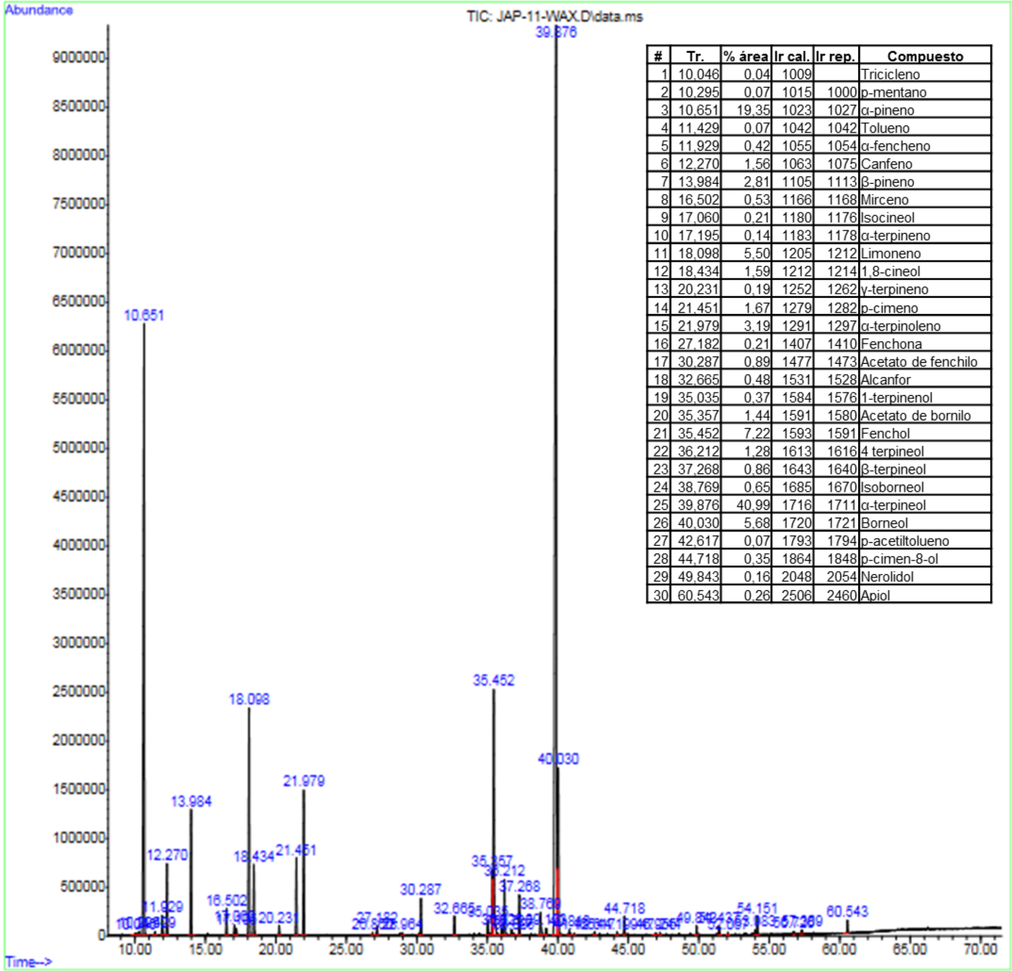

#	Tr.	% área	Ir cal.	Ir rep.	Compuesto
1	10,046	0,04	1009		Tricicleno
2	10,295	0,07	1015	1000	p-mentano
3	10,651	19,35	1023	1027	α-pineno
4	11,429	0,07	1042	1042	Tolueno
5	11,929	0,42	1055	1054	α-fencheno
6	12,270	1,56	1063	1075	Canfeno
7	13,984	2,81	1105	1113	β-pineno
8	16,502	0,53	1166	1168	Mirceno
9	17,060	0,21	1180	1176	Isocineol
10	17,195	0,14	1183	1178	α-terpineno
11	18,098	5,50	1205	1212	Limoneno
12	18,434	1,59	1212	1214	1,8-cineol
13	20,231	0,19	1252	1262	γ-terpineno
14	21,451	1,67	1279	1282	p-cimeno
15	21,979	3,19	1291	1297	α-terpinoleno
16	27,182	0,21	1407	1410	Fenchona
17	30,287	0,89	1477	1473	Acetato de fenchilo
18	32,665	0,48	1531	1528	Alcanfor
19	35,035	0,37	1584	1576	1-terpinenol
20	35,357	1,44	1591	1580	Acetato de bornilo
21	35,452	7,22	1593	1591	Fenchol
22	36,212	1,28	1613	1616	4 terpineol
23	37,268	0,86	1643	1640	β-terpineol
24	38,769	0,65	1685	1670	Isoborneol
25	39,876	40,99	1716	1711	α-terpineol
26	40,030	5,68	1720	1721	Borneol
27	42,617	0,07	1793	1794	p-acetiltolueno
28	44,718	0,35	1864	1848	p-cimen-8-ol
29	49,843	0,16	2048	2054	Nerolidol
30	60,543	0,26	2506	2460	Apiol

Chromatographic profiles obtained for the essential oil of *Eucalyptus* sp. leaves

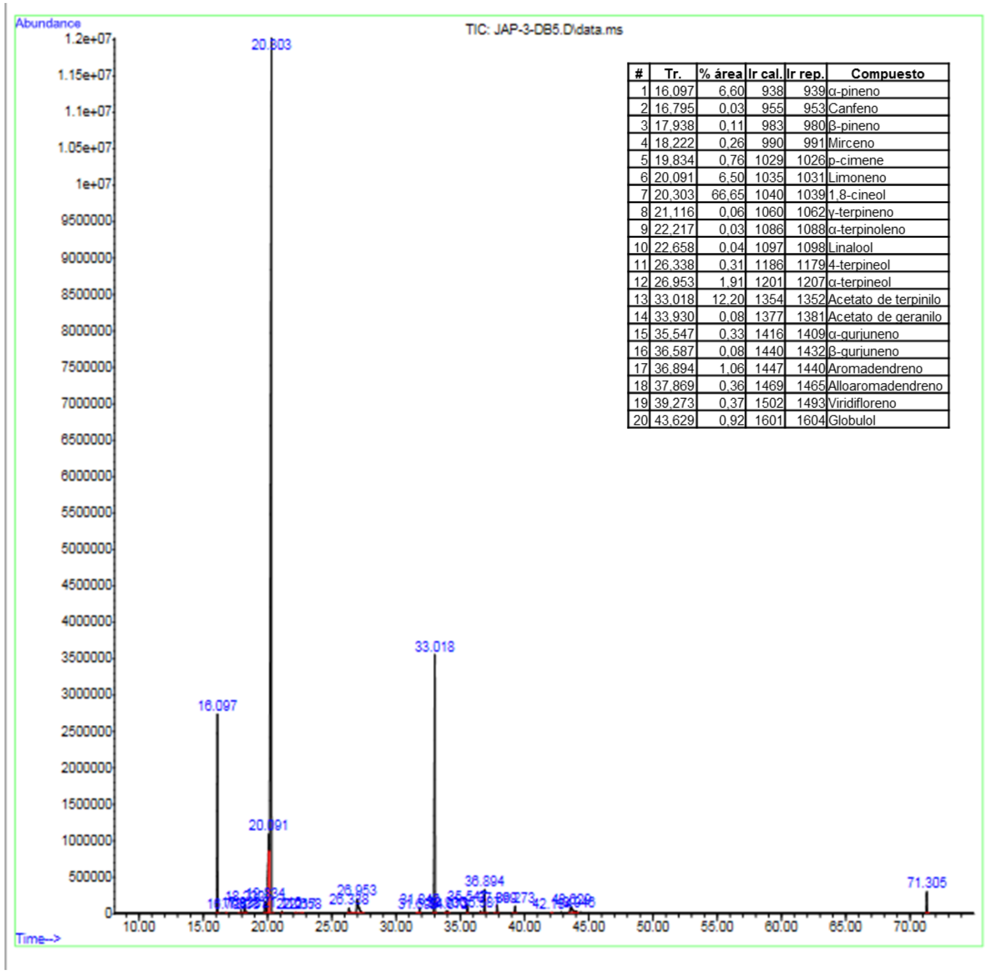

#	Tr.	% área	Ir cal.	Ir rep.	Compuesto
1	16,097	6,60	938	939	α-pineno
2	16,795	0,03	955	953	Canfeno
3	17,938	0,11	983	980	β-pineno
4	18,222	0,26	990	991	Mirceno
5	19,834	0,76	1029	1026	p-cimene
6	20,091	6,50	1035	1031	Limoneno
7	20,303	66,65	1040	1039	1,8-cineol
8	21,116	0,06	1060	1062	γ-terpineno
9	22,217	0,03	1086	1088	α-terpinoleno
10	22,658	0,04	1097	1098	Linalool
11	26,338	0,31	1186	1179	4-terpineol
12	26,953	1,91	1201	1207	α-terpineol
13	33,018	12,20	1354	1352	Acetato de terpinilo
14	33,930	0,08	1377	1381	Acetato de geranilo
15	35,547	0,33	1416	1409	α-gurjuneno
16	36,587	0,08	1440	1432	β-gurjuneno
17	36,894	1,06	1447	1440	Aromadendreno
18	37,869	0,36	1469	1465	Alloaromadendreno
19	39,273	0,37	1502	1493	Viridifloreno
20	43,629	0,92	1601	1604	Globulol

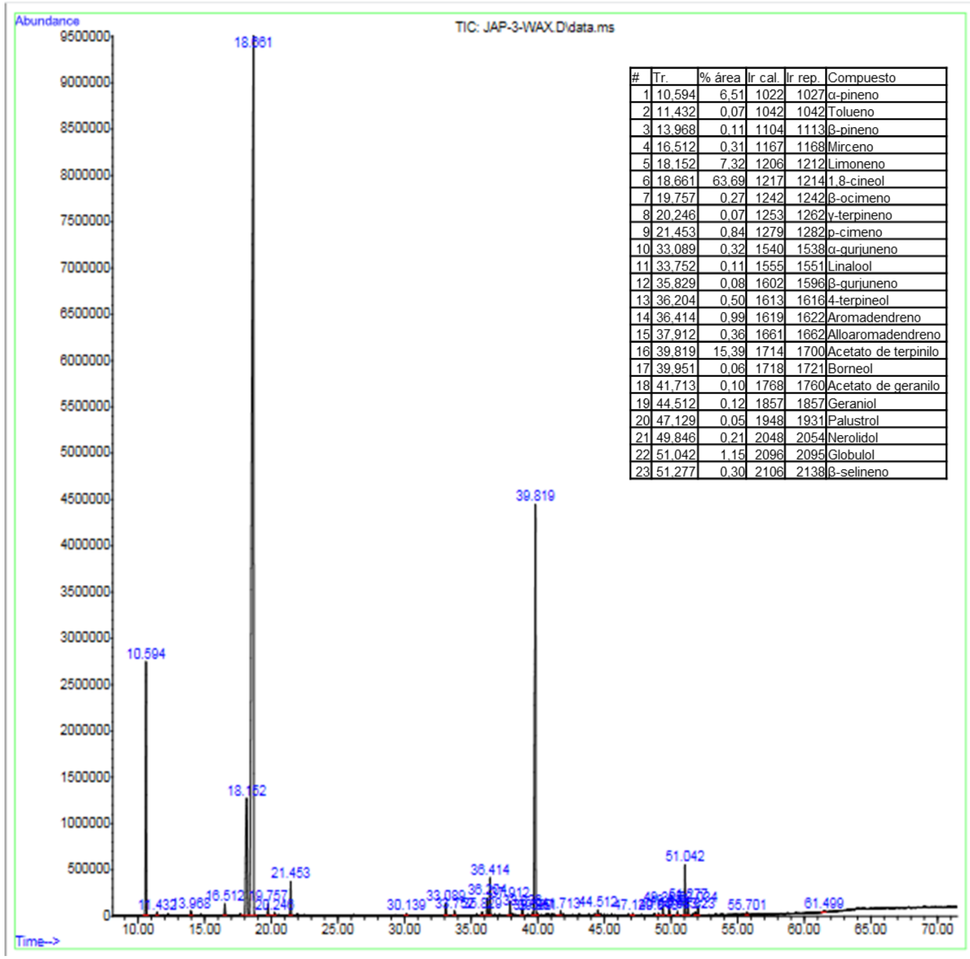

#	Tr.	% área	Ir cal.	Ir rep.	Compuesto
1	10,594	6,51	1022	1027	α-pineno
2	11,432	0,07	1042	1042	Tolueno
3	13,968	0,11	1104	1113	β-pineno
4	16,512	0,31	1167	1168	Mirceno
5	18,152	7,32	1206	1212	Limoneno
6	18,661	63,69	1217	1214	1,8-cineol
7	19,757	0,27	1242	1242	β-ocimeno
8	20,246	0,07	1253	1262	γ-terpineno
9	21,453	0,84	1279	1282	p-cimeno
10	33,089	0,32	1540	1538	α-gurjuneno
11	33,752	0,11	1555	1551	Linalool
12	35,829	0,08	1602	1596	β-gurjuneno
13	36,204	0,50	1613	1616	4-terpineol
14	36,414	0,99	1619	1622	Aromadendreno
15	37,912	0,36	1661	1662	Alloaromadendreno
16	39,819	15,39	1714	1700	Acetato de terpinilo
17	39,951	0,06	1718	1721	Borneol
18	41,713	0,10	1768	1760	Acetato de geranilo
19	44,512	0,12	1857	1857	Geraniol
20	47,129	0,05	1948	1931	Palustrol
21	49,846	0,21	2048	2054	Nerolidol
22	51,042	1,15	2096	2095	Globulol
23	51,277	0,30	2106	2138	β-selineno

Chromatographic profiles obtained for the essential oil of *P. el-metanum* leaves

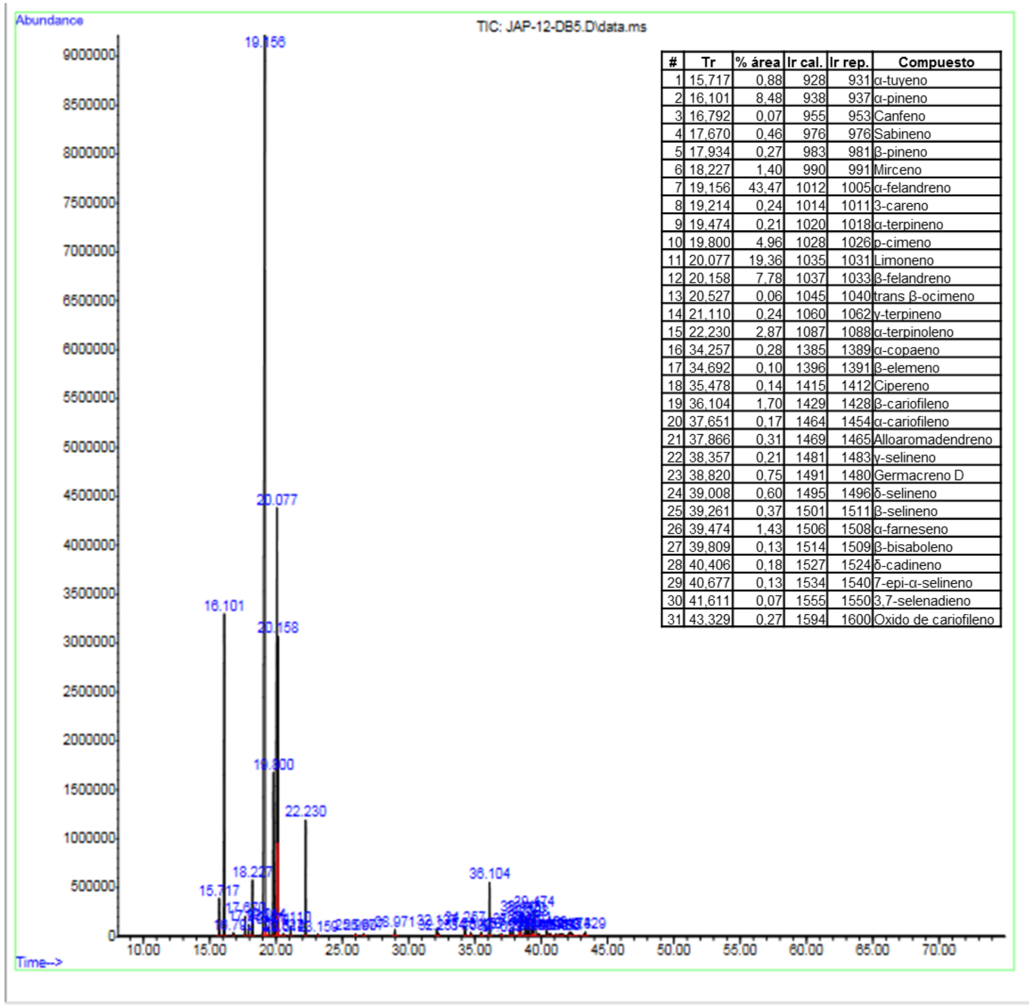

#	Tr	% área	Ir cal.	Ir rep.	Compuesto
1	15,717	0,88	928	931	α-tuyeno
2	16,101	8,48	938	937	α-pineno
3	16,792	0,07	955	953	Canfeno
4	17,670	0,46	976	976	Sabineno
5	17,934	0,27	983	981	β-pineno
6	18,227	1,40	990	991	Mirceno
7	19,156	43,47	1012	1005	α-felandreno
8	19,214	0,24	1014	1011	3-careno
9	19,474	0,21	1020	1018	α-terpineno
10	19,800	4,96	1028	1026	p-cimeno
11	20,077	19,36	1035	1031	Limoneno
12	20,158	7,78	1037	1033	β-felandreno
13	20,527	0,06	1045	1040	trans β-ocimeno
14	21,110	0,24	1060	1062	γ-terpineno
15	22,230	2,87	1087	1088	α-terpinoleno
16	34,257	0,28	1385	1389	α-copaeno
17	34,692	0,10	1396	1391	β-elemeno
18	35,478	0,14	1415	1412	Cipereno
19	36,104	1,70	1429	1428	β-cariofileno
20	37,651	0,17	1464	1454	α-cariofileno
21	37,866	0,31	1469	1465	Alloaromadendreno
22	38,357	0,21	1481	1483	γ-selineno
23	38,820	0,75	1491	1480	Germacreno D
24	39,008	0,60	1495	1496	δ-selineno
25	39,261	0,37	1501	1511	β-selineno
26	39,474	1,43	1506	1508	α-farneseno
27	39,809	0,13	1514	1509	β-bisaboleno
28	40,406	0,18	1527	1524	δ-cadineno
29	40,677	0,13	1534	1540	7-epi-α-selineno
30	41,611	0,07	1555	1550	3,7-selenadieno
31	43,329	0,27	1594	1600	Oxido de cariofileno

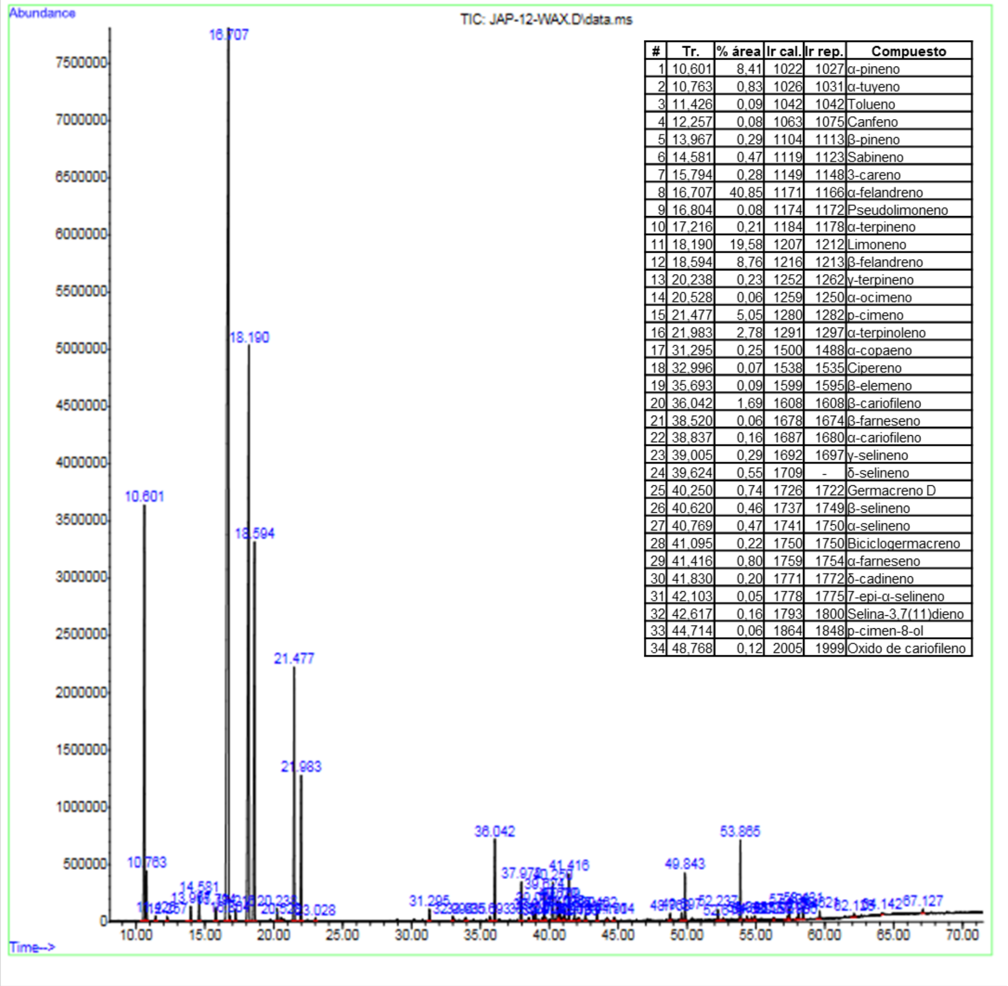

#	Tr.	% área	Ir cal.	Ir rep.	Compuesto
1	10,601	8,41	1022	1027	α-pineno
2	10,763	0,83	1026	1031	α-tuyeno
3	11,426	0,09	1042	1042	Tolueno
4	12,257	0,08	1063	1075	Canfeno
5	13,967	0,29	1104	1113	β-pineno
6	14,581	0,47	1119	1123	Sabineno
7	15,794	0,28	1149	1148	3-careno
8	16,707	40,85	1171	1166	α-felandreno
9	16,804	0,08	1174	1172	Pseudolimoneno
10	17,216	0,21	1184	1178	α-terpineno
11	18,190	19,58	1207	1212	Limoneno
12	18,594	8,76	1216	1213	β-felandreno
13	20,238	0,23	1252	1262	γ-terpineno
14	20,528	0,06	1259	1250	α-ocimeno
15	21,477	5,05	1280	1282	p-cimeno
16	21,983	2,78	1291	1297	α-terpinoleno
17	31,295	0,25	1500	1488	α-copaeno
18	32,996	0,07	1538	1535	Cipereno
19	35,693	0,09	1599	1595	β-elemeno
20	36,042	1,69	1608	1608	β-cariofileno
21	38,520	0,06	1678	1674	β-farneseno
22	38,837	0,16	1687	1680	α-cariofileno
23	39,005	0,29	1692	1697	γ-selineno
24	39,624	0,55	1709	-	δ-selineno
25	40,250	0,74	1726	1722	Germacreno D
26	40,620	0,46	1737	1749	β-selineno
27	40,769	0,47	1741	1750	α-selineno
28	41,095	0,22	1750	1750	Biciclogermacreno
29	41,416	0,80	1759	1754	α-farneseno
30	41,830	0,20	1771	1772	δ-cadineno
31	42,103	0,05	1778	1775	7-epi-α-selineno
32	42,617	0,16	1793	1800	Selina-3,7(11)dieno
33	44,714	0,06	1864	1848	p-cimen-8-ol
34	48,768	0,12	2005	1999	Oxido de cariofileno

Chromatographic profiles obtained for the essential oil of *P. nubigenum* leaves and steams

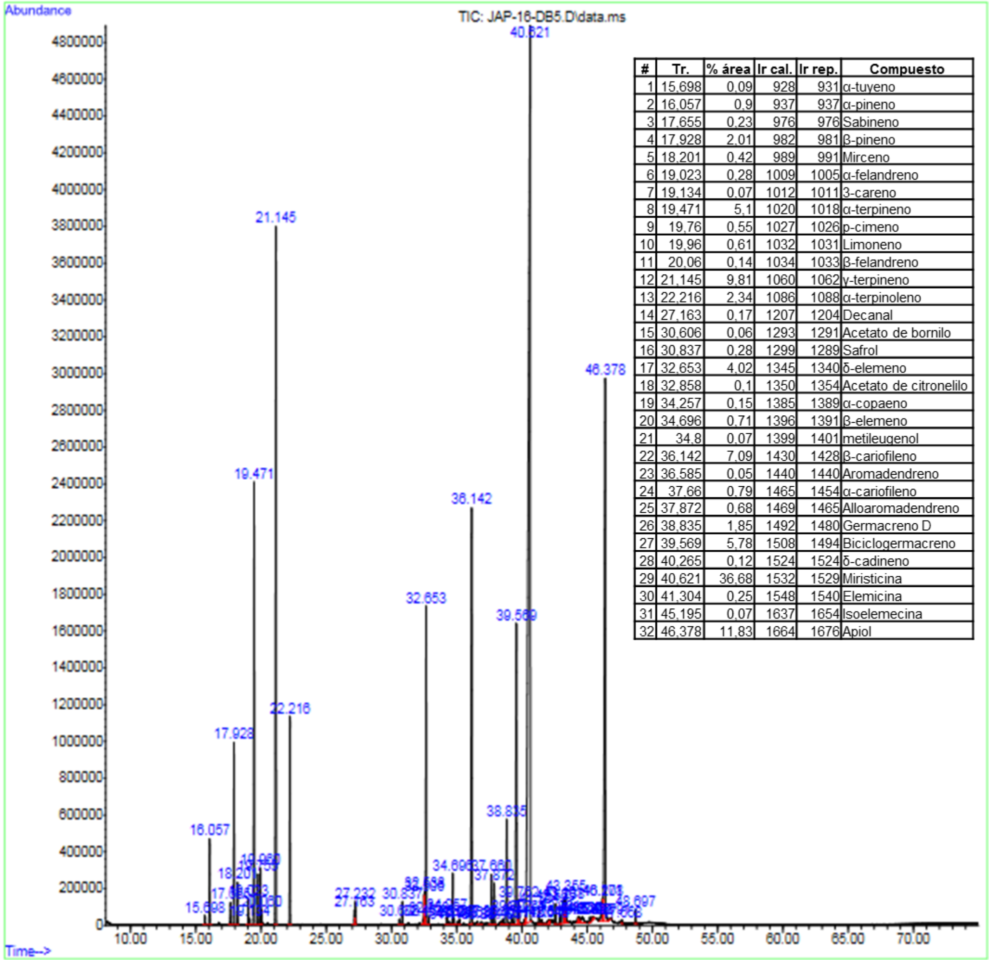

#	Tr.	% área	Ir cal.	Ir rep.	Compuesto
1	15,698	0,09	928	931	α-tuyeno
2	16,057	0,9	937	937	α-pineno
3	17,655	0,23	976	976	Sabineno
4	17,928	2,01	982	981	β-pineno
5	18,201	0,42	989	991	Mirceno
6	19,023	0,28	1009	1005	α-felandreno
7	19,134	0,07	1012	1011	3-careno
8	19,471	5,1	1020	1018	α-terpineno
9	19,76	0,55	1027	1026	p-cimeno
10	19,96	0,61	1032	1031	Limoneno
11	20,06	0,14	1034	1033	β-felandreno
12	21,145	9,81	1060	1062	γ-terpineno
13	22,216	2,34	1086	1088	α-terpinoleno
14	27,163	0,17	1207	1204	Decanal
15	30,606	0,06	1293	1291	Acetato de bornilo
16	30,837	0,28	1299	1289	Safrol
17	32,653	4,02	1345	1340	δ-elemeno
18	32,858	0,1	1350	1354	Acetato de citronelilo
19	34,257	0,15	1385	1389	α-copaeno
20	34,696	0,71	1396	1391	β-elemeno
21	34,8	0,07	1399	1401	metileugenol
22	36,142	7,09	1430	1428	β-cariofileno
23	36,585	0,05	1440	1440	Aromadendreno
24	37,66	0,79	1465	1454	α-cariofileno
25	37,872	0,68	1469	1465	Alloaromadendreno
26	38,835	1,85	1492	1480	Germacreno D
27	39,569	5,78	1508	1494	Biciclogermacreno
28	40,265	0,12	1524	1524	δ-cadineno
29	40,621	36,68	1532	1529	Miristicina
30	41,304	0,25	1548	1540	Elemicina
31	45,195	0,07	1637	1654	Isoelemecina
32	46,378	11,83	1664	1676	Apiol

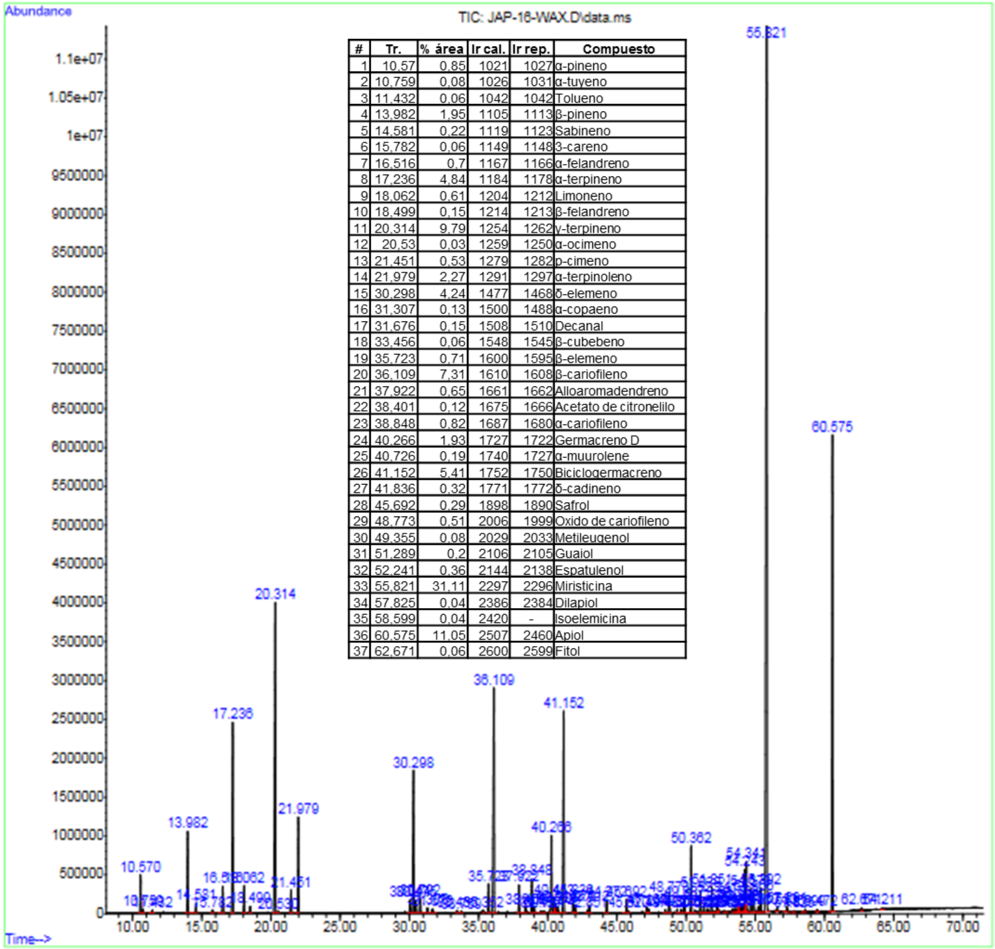

#	Tr.	% área	Ir cal.	Ir rep.	Compuesto
1	10,57	0,85	1021	1027	α-pineno
2	10,759	0,08	1026	1031	α-tuyeno
3	11,432	0,06	1042	1042	Tolueno
4	13,982	1,95	1105	1113	β-pineno
5	14,581	0,22	1119	1123	Sabineno
6	15,782	0,06	1149	1148	3-careno
7	16,516	0,7	1167	1166	α-felandreno
8	17,236	4,84	1184	1178	α-terpineno
9	18,062	0,61	1204	1212	Limoneno
10	18,499	0,15	1214	1213	β-felandreno
11	20,314	9,79	1254	1262	γ-terpineno
12	20,53	0,03	1259	1250	α-ocimeno
13	21,451	0,53	1279	1282	p-cimeno
14	21,979	2,27	1291	1297	α-terpinoleno
15	30,298	4,24	1477	1468	δ-elemeno
16	31,307	0,13	1500	1488	α-copaeno
17	31,676	0,15	1508	1510	Decanal
18	33,456	0,06	1548	1545	β-cubebeno
19	35,723	0,71	1600	1595	β-elemeno
20	36,109	7,31	1610	1608	β-cariofileno
21	37,922	0,65	1661	1662	Alloaromadendreno
22	38,401	0,12	1675	1666	Acetato de citronelilo
23	38,848	0,82	1687	1680	α-cariofileno
24	40,266	1,93	1727	1722	Germacreno D
25	40,726	0,19	1740	1727	α-muurolene
26	41,152	5,41	1752	1750	Biciclogermacreno
27	41,836	0,32	1771	1772	δ-cadineno
28	45,692	0,29	1898	1890	Safrol
29	48,773	0,51	2006	1999	Oxido de cariofileno
30	49,355	0,08	2029	2033	Metileugenol
31	51,289	0,2	2106	2105	Guaiol
32	52,241	0,36	2144	2138	Espatulenol
33	55,821	31,11	2297	2296	Miristicina
34	57,825	0,04	2386	2384	Dilapiol
35	58,599	0,04	2420	-	Isoelemicina
36	60,575	11,05	2507	2460	Apiol
37	62,671	0,06	2600	2599	Fitol

Chromatographic profiles obtained for the essential oil of *P. aduncum* inflorescences

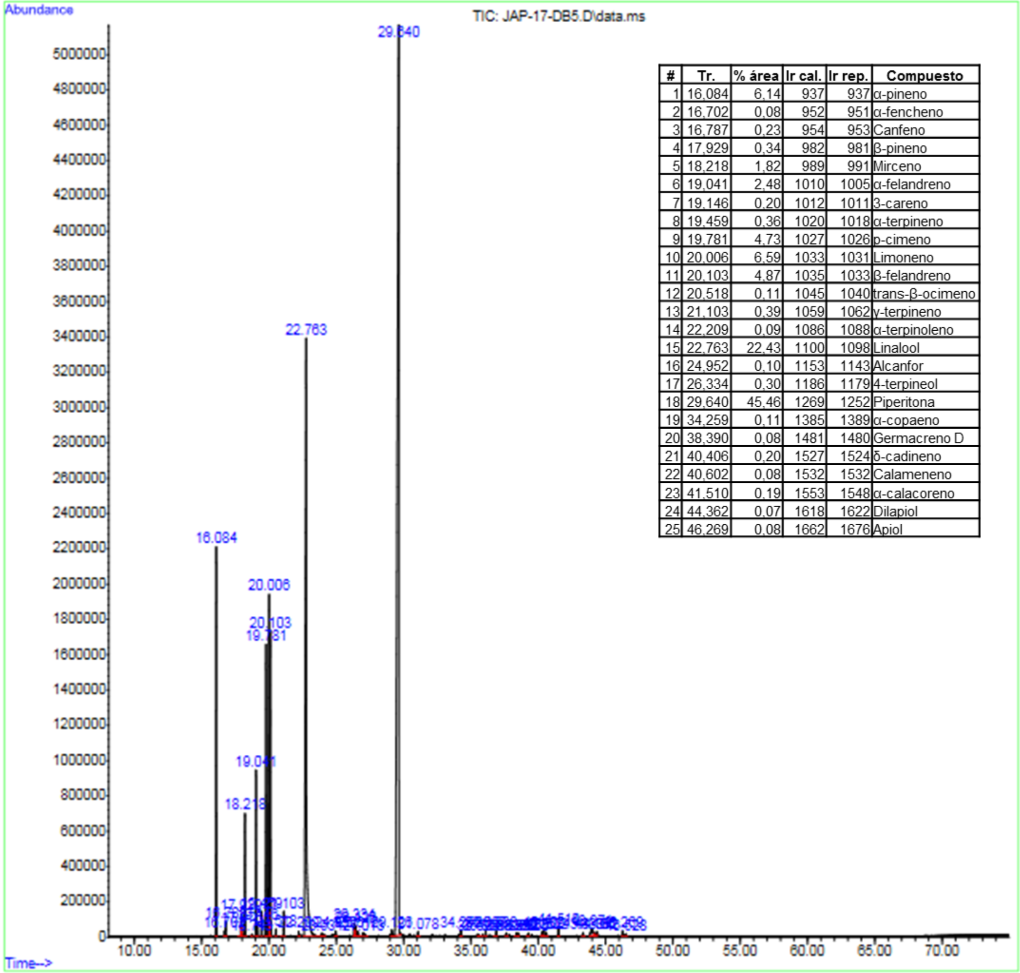

#	Tr.	% área	Ir cal.	Ir rep.	Compuesto
1	16,084	6,14	937	937	α-pineno
2	16,702	0,08	952	951	α-fencheno
3	16,787	0,23	954	953	Canfeno
4	17,929	0,34	982	981	β-pineno
5	18,218	1,82	989	991	Mirceno
6	19,041	2,48	1010	1005	α-felandreno
7	19,146	0,20	1012	1011	3-careno
8	19,459	0,36	1020	1018	α-terpineno
9	19,781	4,73	1027	1026	p-cimeno
10	20,006	6,59	1033	1031	Limoneno
11	20,103	4,87	1035	1033	β-felandreno
12	20,518	0,11	1045	1040	trans-β-ocimeno
13	21,103	0,39	1059	1062	γ-terpineno
14	22,209	0,09	1086	1088	α-terpinoleno
15	22,763	22,43	1100	1098	Linalool
16	24,952	0,10	1153	1143	Alcanfor
17	26,334	0,30	1186	1179	4-terpineol
18	29,640	45,46	1269	1252	Piperitona
19	34,259	0,11	1385	1389	α-copaeno
20	38,390	0,08	1481	1480	Germacreno D
21	40,406	0,20	1527	1524	δ-cadineno
22	40,602	0,08	1532	1532	Calameneno
23	41,510	0,19	1553	1548	α-calacoreno
24	44,362	0,07	1618	1622	Dilapiol
25	46,269	0,08	1662	1676	Apiol

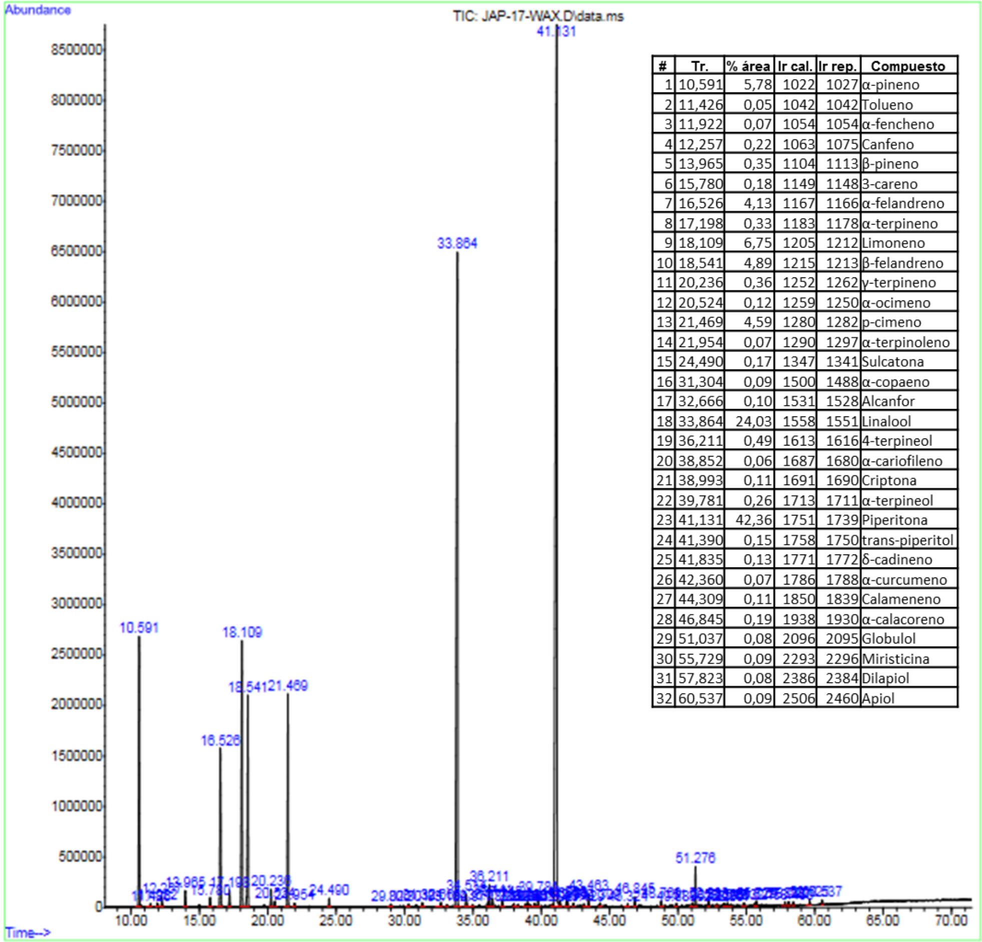

#	Tr.	% área	Ir cal.	Ir rep.	Compuesto
1	10,591	5,78	1022	1027	α-pineno
2	11,426	0,05	1042	1042	Tolueno
3	11,922	0,07	1054	1054	α-fencheno
4	12,257	0,22	1063	1075	Canfeno
5	13,965	0,35	1104	1113	β-pineno
6	15,780	0,18	1149	1148	3-careno
7	16,526	4,13	1167	1166	α-felandreno
8	17,198	0,33	1183	1178	α-terpineno
9	18,109	6,75	1205	1212	Limoneno
10	18,541	4,89	1215	1213	β-felandreno
11	20,236	0,36	1252	1262	γ-terpineno
12	20,524	0,12	1259	1250	α-ocimeno
13	21,469	4,59	1280	1282	p-cimeno
14	21,954	0,07	1290	1297	α-terpinoleno
15	24,490	0,17	1347	1341	Sulcatona
16	31,304	0,09	1500	1488	α-copaeno
17	32,666	0,10	1531	1528	Alcanfor
18	33,864	24,03	1558	1551	Linalool
19	36,211	0,49	1613	1616	4-terpineol
20	38,852	0,06	1687	1680	α-cariofileno
21	38,993	0,11	1691	1690	Criptona
22	39,781	0,26	1713	1711	α-terpineol
23	41,131	42,36	1751	1739	Piperitona
24	41,390	0,15	1758	1750	trans-piperitol
25	41,835	0,13	1771	1772	δ-cadineno
26	42,360	0,07	1786	1788	α-curcumeno
27	44,309	0,11	1850	1839	Calameneno
28	46,845	0,19	1938	1930	α-calacoreno
29	51,037	0,08	2096	2095	Globulol
30	55,729	0,09	2293	2296	Miristicina
31	57,823	0,08	2386	2384	Dilapiol
32	60,537	0,09	2506	2460	Apiol

Chromatographic profiles obtained for the essential oil of *P. asperiusculum* inflorescences

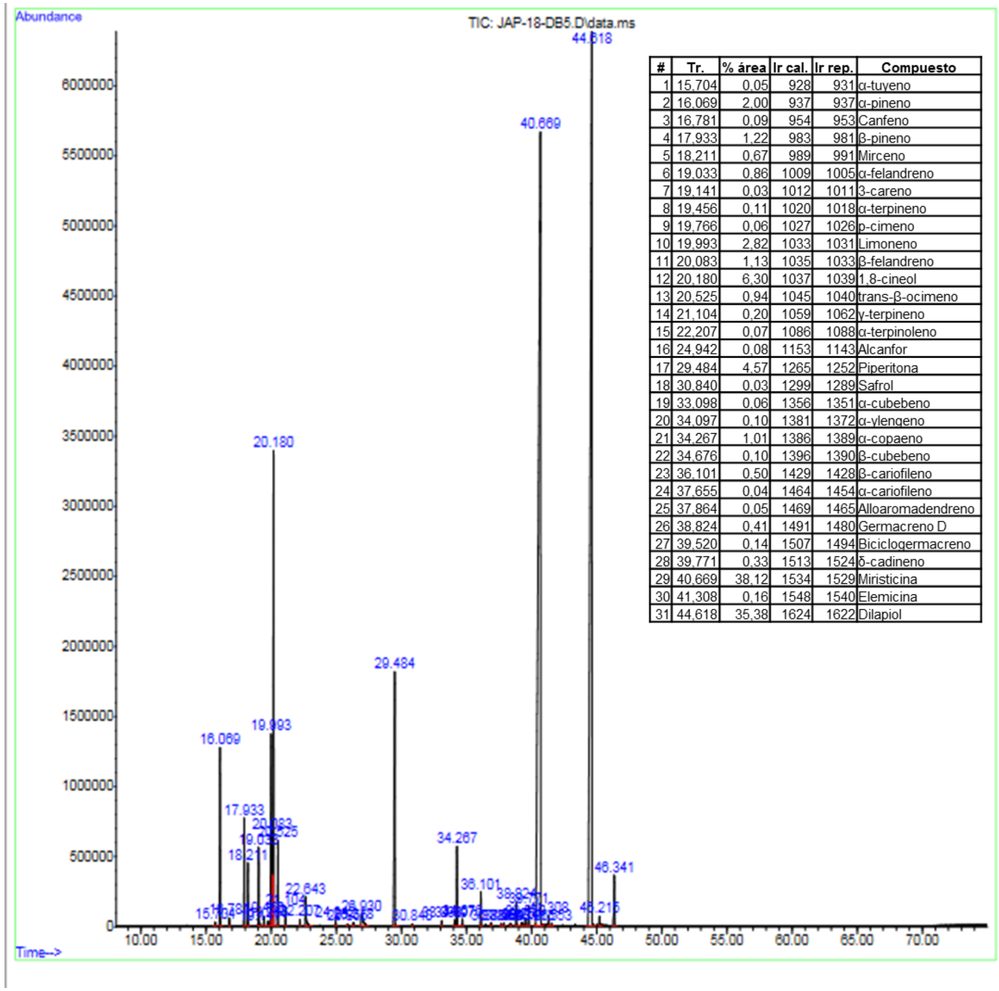

#	Tr.	% área	Ir cal.	Ir rep.	Compuesto
1	15,704	0,05	928	931	α-tuyeno
2	16,069	2,00	937	937	α-pineno
3	16,781	0,09	954	953	Canfeno
4	17,933	1,22	983	981	β-pineno
5	18,211	0,67	989	991	Mirceno
6	19,033	0,86	1009	1005	α-felandreno
7	19,141	0,03	1012	1011	3-careno
8	19,456	0,11	1020	1018	α-terpineno
9	19,766	0,06	1027	1026	p-cimeno
10	19,993	2,82	1033	1031	Limoneno
11	20,083	1,13	1035	1033	β-felandreno
12	20,180	6,30	1037	1039	1,8-cineol
13	20,525	0,94	1045	1040	trans-β-ocimeno
14	21,104	0,20	1059	1062	γ-terpineno
15	22,207	0,07	1086	1088	α-terpinoleno
16	24,942	0,08	1153	1143	Alcanfor
17	29,484	4,57	1265	1252	Piperitona
18	30,840	0,03	1299	1289	Safrol
19	33,098	0,06	1356	1351	α-cubebeno
20	34,097	0,10	1381	1372	α-ylengeno
21	34,267	1,01	1386	1389	α-copaeno
22	34,676	0,10	1396	1390	β-cubebeno
23	36,101	0,50	1429	1428	β-cariofileno
24	37,655	0,04	1464	1454	α-cariofileno
25	37,864	0,05	1469	1465	Alloaromadendreno
26	38,824	0,41	1491	1480	Germacreno D
27	39,520	0,14	1507	1494	Biciclogermacreno
28	39,771	0,33	1513	1524	δ-cadineno
29	40,669	38,12	1534	1529	Miristicina
30	41,308	0,16	1548	1540	Elemicina
31	44,618	35,38	1624	1622	Dilapiol

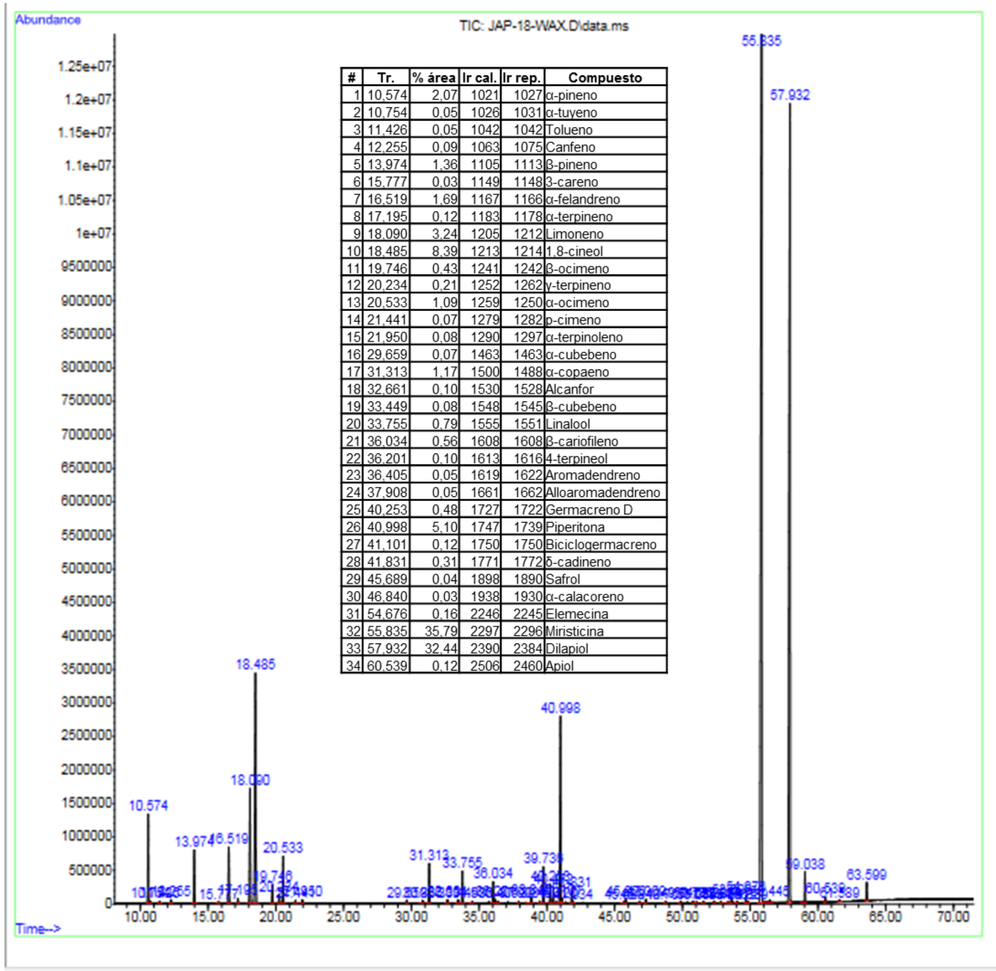

#	Tr.	% área	Ir cal.	Ir rep.	Compuesto
1	10,574	2,07	1021	1027	α-pineno
2	10,754	0,05	1026	1031	α-tuyeno
3	11,426	0,05	1042	1042	Tolueno
4	12,255	0,09	1063	1075	Canfeno
5	13,974	1,36	1105	1113	β-pineno
6	15,777	0,03	1149	1148	3-careno
7	16,519	1,69	1167	1166	α-felandreno
8	17,195	0,12	1183	1178	α-terpineno
9	18,090	3,24	1205	1212	Limoneno
10	18,485	8,39	1213	1214	1,8-cineol
11	19,746	0,43	1241	1242	β-ocimeno
12	20,234	0,21	1252	1262	γ-terpineno
13	20,533	1,09	1259	1250	α-ocimeno
14	21,441	0,07	1279	1282	p-cimeno
15	21,950	0,08	1290	1297	α-terpinoleno
16	29,659	0,07	1463	1463	α-cubebeno
17	31,313	1,17	1500	1488	α-copaeno
18	32,661	0,10	1530	1528	Alcanfor
19	33,449	0,08	1548	1545	β-cubebeno
20	33,755	0,79	1555	1551	Linalool
21	36,034	0,56	1608	1608	β-cariofileno
22	36,201	0,10	1613	1616	4-terpineol
23	36,405	0,05	1619	1622	Aromadendreno
24	37,908	0,05	1661	1662	Alloaromadendreno
25	40,253	0,48	1727	1722	Germacreno D
26	40,998	5,10	1747	1739	Piperitona
27	41,101	0,12	1750	1750	Biciclogermacreno
28	41,831	0,31	1771	1772	δ-cadineno
29	45,689	0,04	1898	1890	Safrol
30	46,840	0,03	1938	1930	α-calacoreno
31	54,676	0,16	2246	2245	Elemecina
32	55,835	35,79	2297	2296	Miristicina
33	57,932	32,44	2390	2384	Dilapiol
34	60,539	0,12	2506	2460	Apiol

Chromatographic profiles obtained for the essential oil of *P. pertomentellum* inflorescences

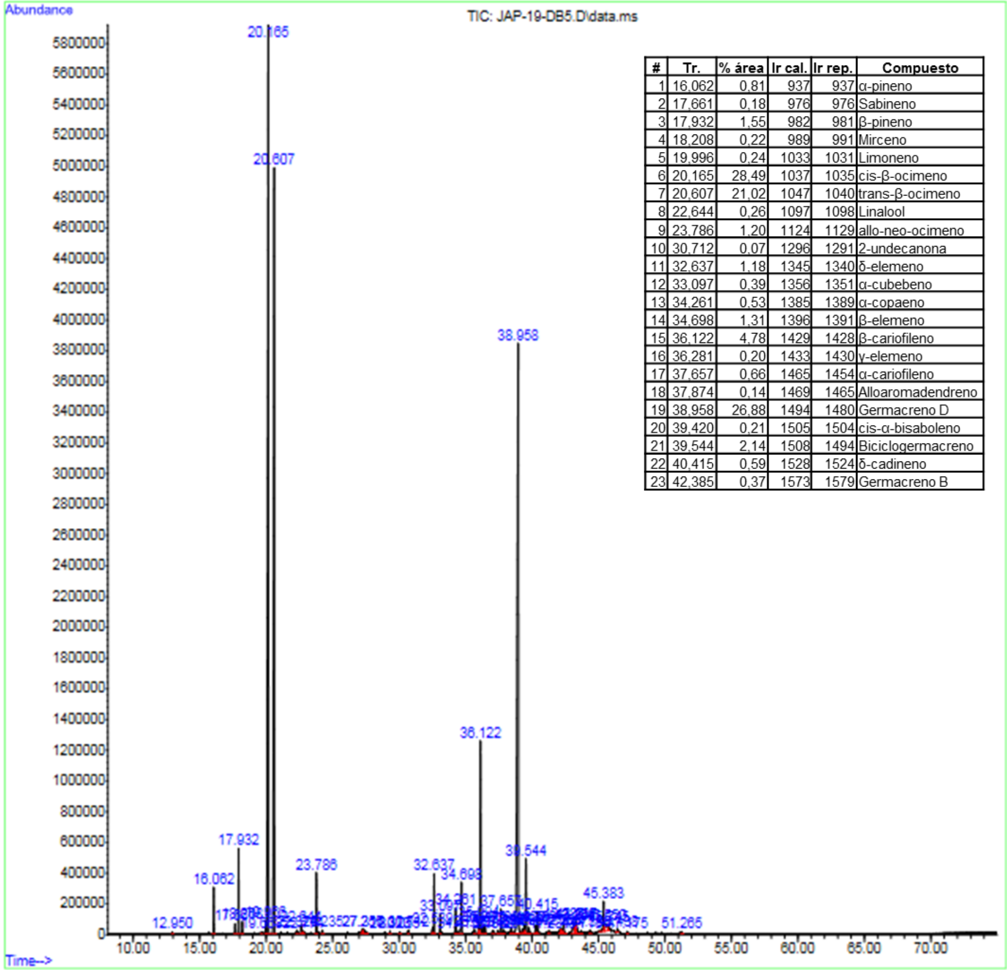

#	Tr.	% área	Ir cal.	Ir rep.	Compuesto
1	16,062	0,81	937	937	α-pineno
2	17,661	0,18	976	976	Sabineno
3	17,932	1,55	982	981	β-pineno
4	18,208	0,22	989	991	Mirceno
5	19,996	0,24	1033	1031	Limoneno
6	20,165	28,49	1037	1035	cis-β-ocimeno
7	20,607	21,02	1047	1040	trans-β-ocimeno
8	22,644	0,26	1097	1098	Linalool
9	23,786	1,20	1124	1129	allo-neo-ocimeno
10	30,712	0,07	1296	1291	2-undecanona
11	32,637	1,18	1345	1340	δ-elemeno
12	33,097	0,39	1356	1351	α-cubebeno
13	34,261	0,53	1385	1389	α-copaeno
14	34,698	1,31	1396	1391	β-elemeno
15	36,122	4,78	1429	1428	β-cariofileno
16	36,281	0,20	1433	1430	γ-elemeno
17	37,657	0,66	1465	1454	α-cariofileno
18	37,874	0,14	1469	1465	Alloaromadendreno
19	38,958	26,88	1494	1480	Germacreno D
20	39,420	0,21	1505	1504	cis-α-bisaboleno
21	39,544	2,14	1508	1494	Biciclogermacreno
22	40,415	0,59	1528	1524	δ-cadineno
23	42,385	0,37	1573	1579	Germacreno B

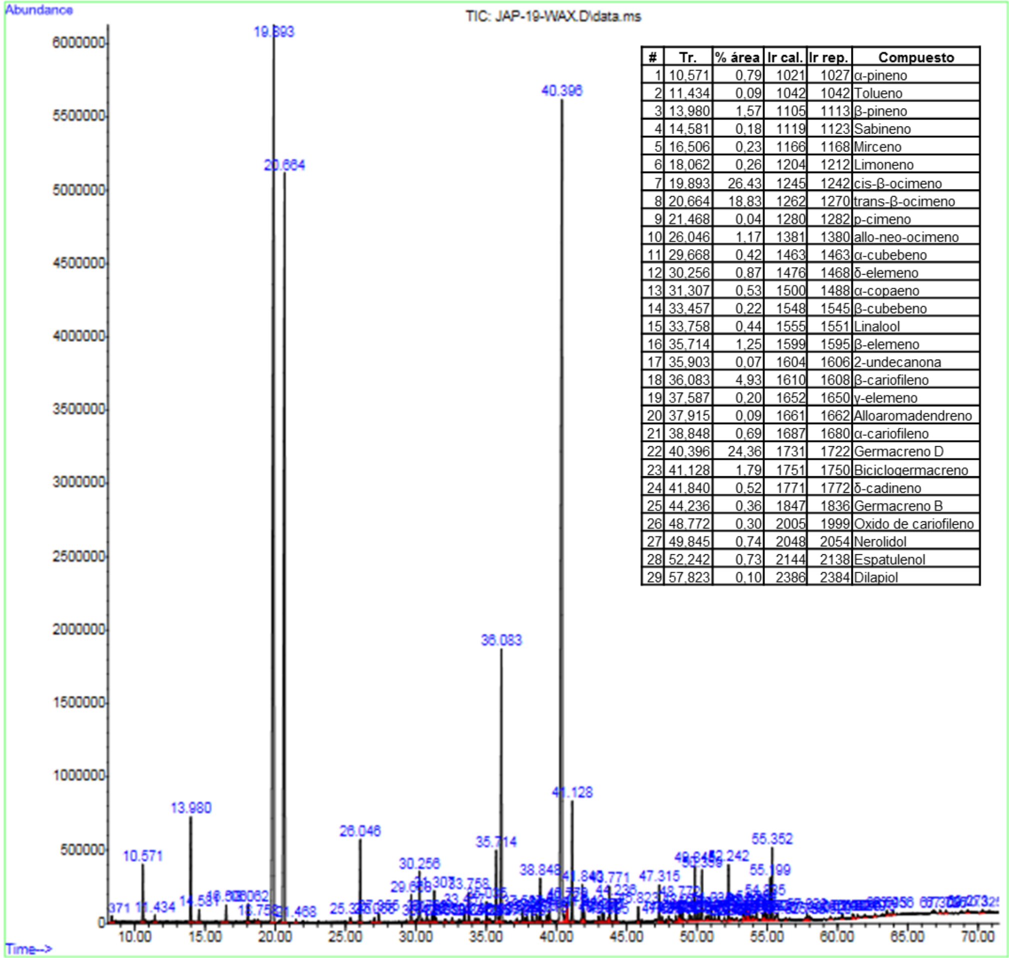

#	Tr.	% área	Ir cal.	Ir rep.	Compuesto
1	10,571	0,79	1021	1027	α-pineno
2	11,434	0,09	1042	1042	Tolueno
3	13,980	1,57	1105	1113	β-pineno
4	14,581	0,18	1119	1123	Sabineno
5	16,506	0,23	1166	1168	Mirceno
6	18,062	0,26	1204	1212	Limoneno
7	19,893	26,43	1245	1242	cis-β-ocimeno
8	20,664	18,83	1262	1270	trans-β-ocimeno
9	21,468	0,04	1280	1282	p-cimeno
10	26,046	1,17	1381	1380	allo-neo-ocimeno
11	29,668	0,42	1463	1463	α-cubebeno
12	30,256	0,87	1476	1468	δ-elemeno
13	31,307	0,53	1500	1488	α-copaeno
14	33,457	0,22	1548	1545	β-cubebeno
15	33,758	0,44	1555	1551	Linalool
16	35,714	1,25	1599	1595	β-elemeno
17	35,903	0,07	1604	1606	2-undecanona
18	36,083	4,93	1610	1608	β-cariofileno
19	37,587	0,20	1652	1650	γ-elemeno
20	37,915	0,09	1661	1662	Alloaromadendreno
21	38,848	0,69	1687	1680	α-cariofileno
22	40,396	24,36	1731	1722	Germacreno D
23	41,128	1,79	1751	1750	Biciclogermacreno
24	41,840	0,52	1771	1772	δ-cadineno
25	44,236	0,36	1847	1836	Germacreno B
26	48,772	0,30	2005	1999	Oxido de cariofileno
27	49,845	0,74	2048	2054	Nerolidol
28	52,242	0,73	2144	2138	Espatulenol
29	57,823	0,10	2386	2384	Dilapiol

Chromatographic profiles obtained for the essential oil of *C. nardus* leaves

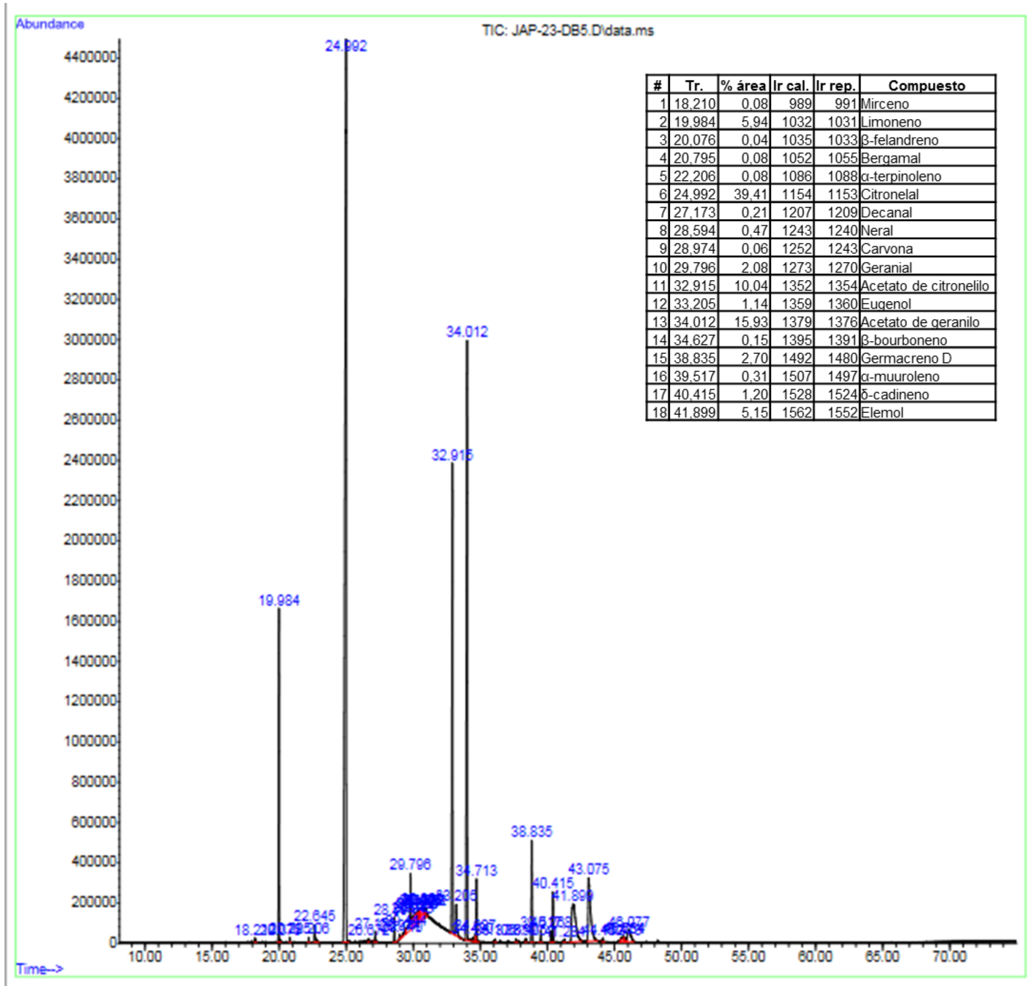

#	Tr.	% área	Ir cal.	Ir rep.	Compuesto
1	18,210	0,08	989	991	Mirceno
2	19,984	5,94	1032	1031	Limoneno
3	20,076	0,04	1035	1033	β-felandreno
4	20,795	0,08	1052	1055	Bergamal
5	22,206	0,08	1086	1088	α-terpinoleno
6	24,992	39,41	1154	1153	Citronelal
7	27,173	0,21	1207	1209	Decanal
8	28,594	0,47	1243	1240	Neral
9	28,974	0,06	1252	1243	Carvona
10	29,796	2,08	1273	1270	Geranial
11	32,915	10,04	1352	1354	Acetato de citronelilo
12	33,205	1,14	1359	1360	Eugenol
13	34,012	15,93	1379	1376	Acetato de geranilo
14	34,627	0,15	1395	1391	β-bourboneno
15	38,835	2,70	1492	1480	Germacreno D
16	39,517	0,31	1507	1497	α-muuroleno
17	40,415	1,20	1528	1524	δ-cadineno
18	41,899	5,15	1562	1552	Elemol

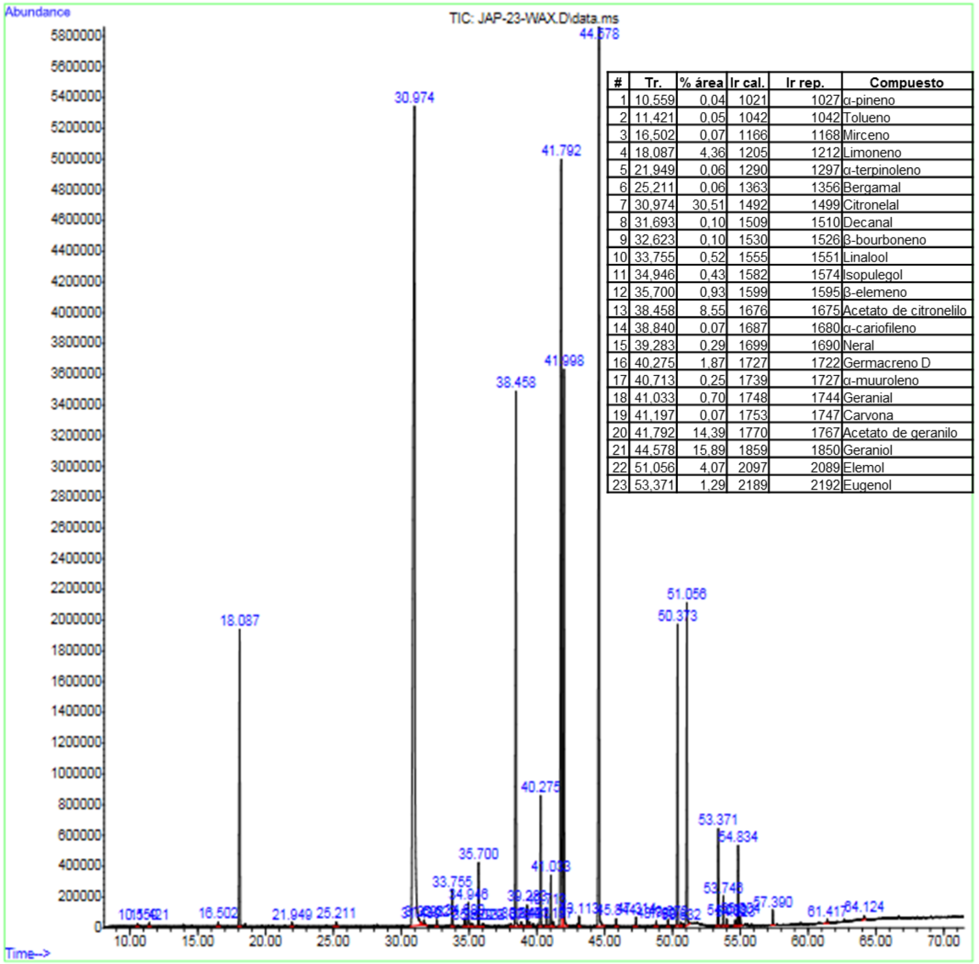

#	Tr.	% área	Ir cal.	Ir rep.	Compuesto
1	10,559	0,04	1021	1027	α-pineno
2	11,421	0,05	1042	1042	Tolueno
3	16,502	0,07	1166	1168	Mirceno
4	18,087	4,36	1205	1212	Limoneno
5	21,949	0,06	1290	1297	α-terpinoleno
6	25,211	0,06	1363	1356	Bergamal
7	30,974	30,51	1492	1499	Citronelal
8	31,693	0,10	1509	1510	Decanal
9	32,623	0,10	1530	1526	β-bourboneno
10	33,755	0,52	1555	1551	Linalool
11	34,946	0,43	1582	1574	Isopulegol
12	35,700	0,93	1599	1595	β-elemeno
13	38,458	8,55	1676	1675	Acetato de citronelilo
14	38,840	0,07	1687	1680	α-cariofileno
15	39,283	0,29	1699	1690	Neral
16	40,275	1,87	1727	1722	Germacreno D
17	40,713	0,25	1739	1727	α-muuroleno
18	41,033	0,70	1748	1744	Geranial
19	41,197	0,07	1753	1747	Carvona
20	41,792	14,39	1770	1767	Acetato de geranilo
21	44,578	15,89	1859	1850	Geraniol
22	51,056	4,07	2097	2089	Elemol
23	53,371	1,29	2189	2192	Eugenol

Chromatographic profiles obtained for the essential oil of *C. citratus* leaves

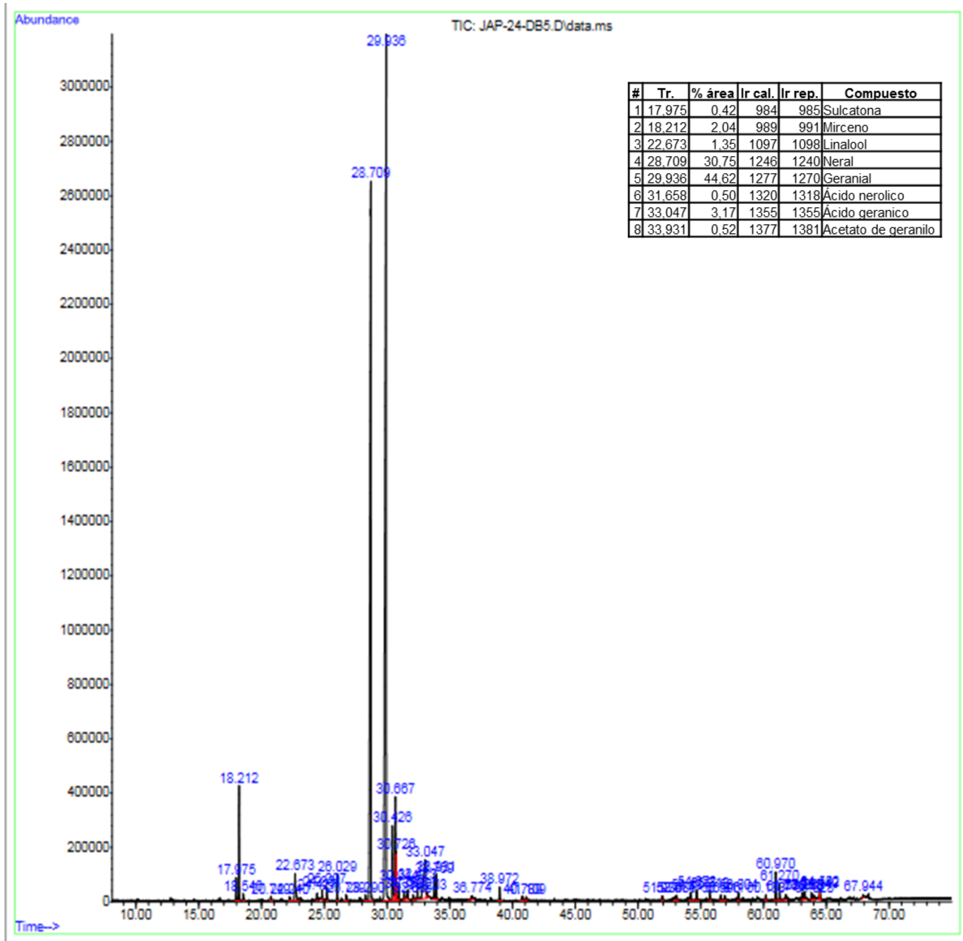

#	Tr.	% área	Ir cal.	Ir rep.	Compuesto
1	17,975	0,42	984	985	Sulcatona
2	18,212	2,04	989	991	Mirceno
3	22,673	1,35	1097	1098	Linalool
4	28,709	30,75	1246	1240	Neral
5	29,936	44,62	1277	1270	Geranial
6	31,658	0,50	1320	1318	Ácido nerolico
7	33,047	3,17	1355	1355	Ácido geranico
8	33,931	0,52	1377	1381	Acetato de geranilo

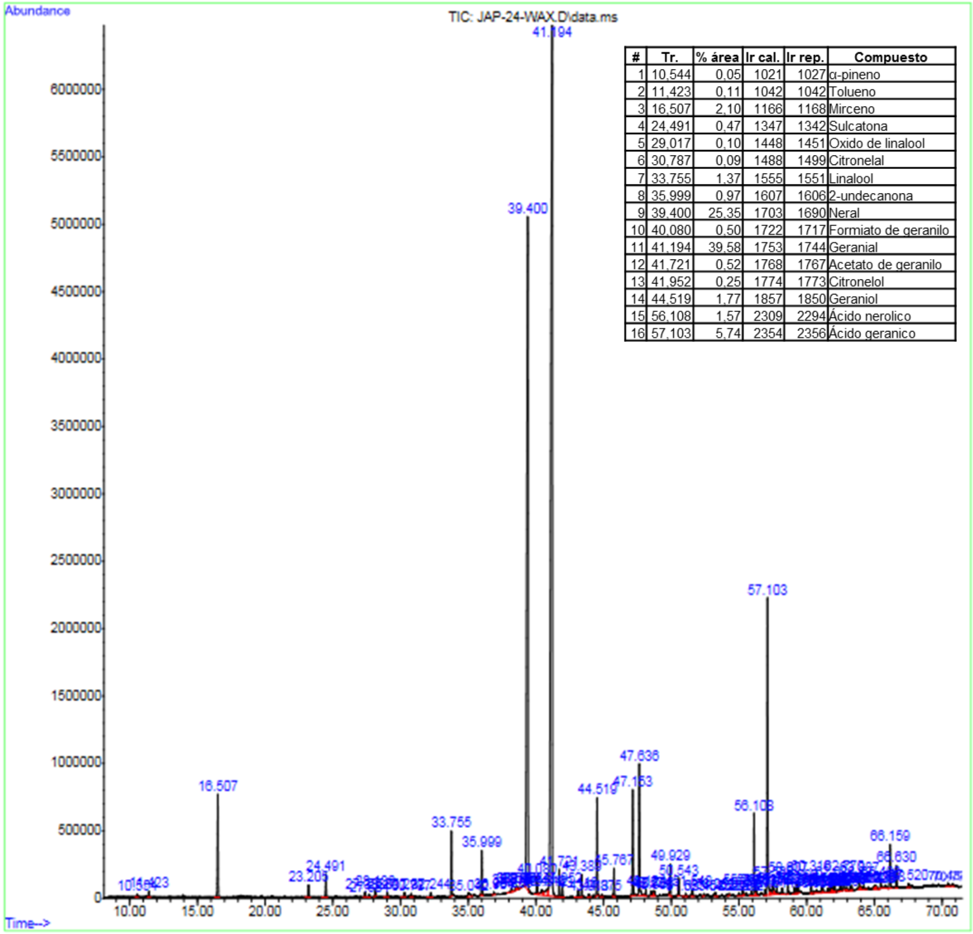

#	Tr.	% área	Ir cal.	Ir rep.	Compuesto
1	10,544	0,05	1021	1027	α-pineno
2	11,423	0,11	1042	1042	Tolueno
3	16,507	2,10	1166	1168	Mirceno
4	24,491	0,47	1347	1342	Sulcatona
5	29,017	0,10	1448	1451	Oxido de linalool
6	30,787	0,09	1488	1499	Citronelal
7	33,755	1,37	1555	1551	Linalool
8	35,999	0,97	1607	1606	2-undecanona
9	39,400	25,35	1703	1690	Neral
10	40,080	0,50	1722	1717	Formiato de geranilo
11	41,194	39,58	1753	1744	Geranial
12	41,721	0,52	1768	1767	Acetato de geranilo
13	41,952	0,25	1774	1773	Citronelol
14	44,519	1,77	1857	1850	Geraniol
15	56,108	1,57	2309	2294	Ácido nerolico
16	57,103	5,74	2354	2356	Ácido geranico

Chromatographic profiles obtained for the essential oil of *C.* × *sinensis* leaves

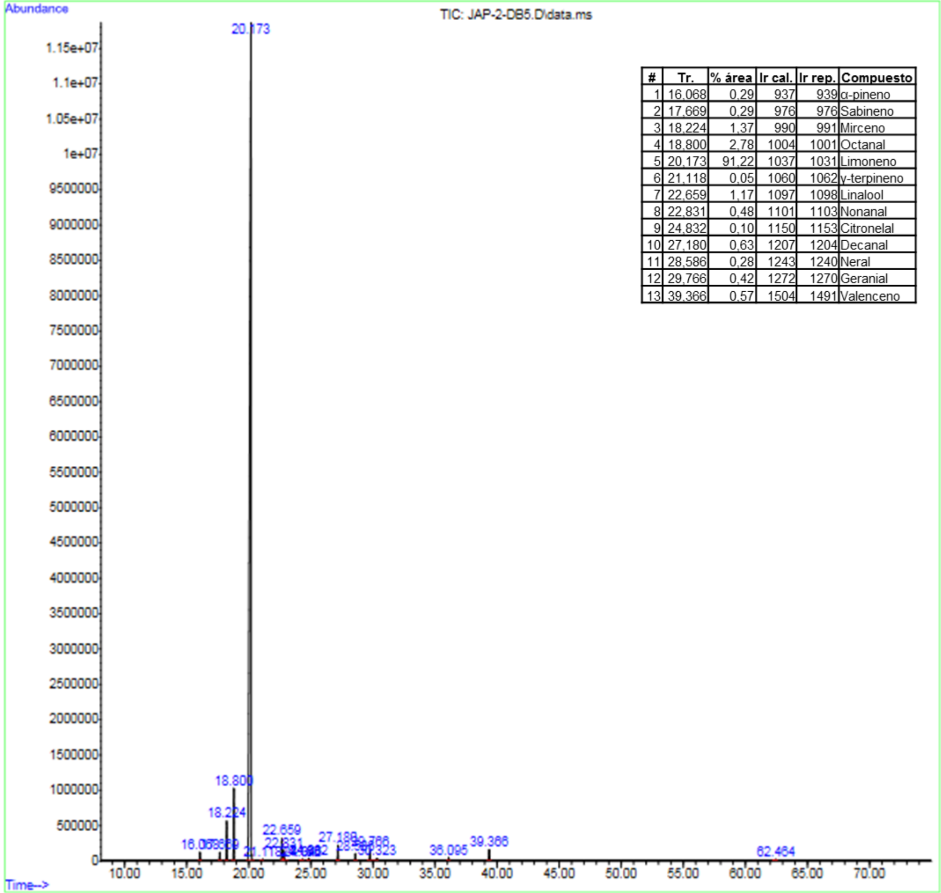

#	Tr.	% área	Ir cal.	Ir rep.	Compuesto
1	16,068	0,29	937	939	α-pineno
2	17,669	0,29	976	976	Sabineno
3	18,224	1,37	990	991	Mirceno
4	18,800	2,78	1004	1001	Octanal
5	20,173	91,22	1037	1031	Limoneno
6	21,118	0,05	1060	1062	γ-terpineno
7	22,659	1,17	1097	1098	Linalool
8	22,831	0,48	1101	1103	Nonanal
9	24,832	0,10	1150	1153	Citronelal
10	27,180	0,63	1207	1204	Decanal
11	28,586	0,28	1243	1240	Neral
12	29,766	0,42	1272	1270	Geranial
13	39,366	0,57	1504	1491	Valenceno

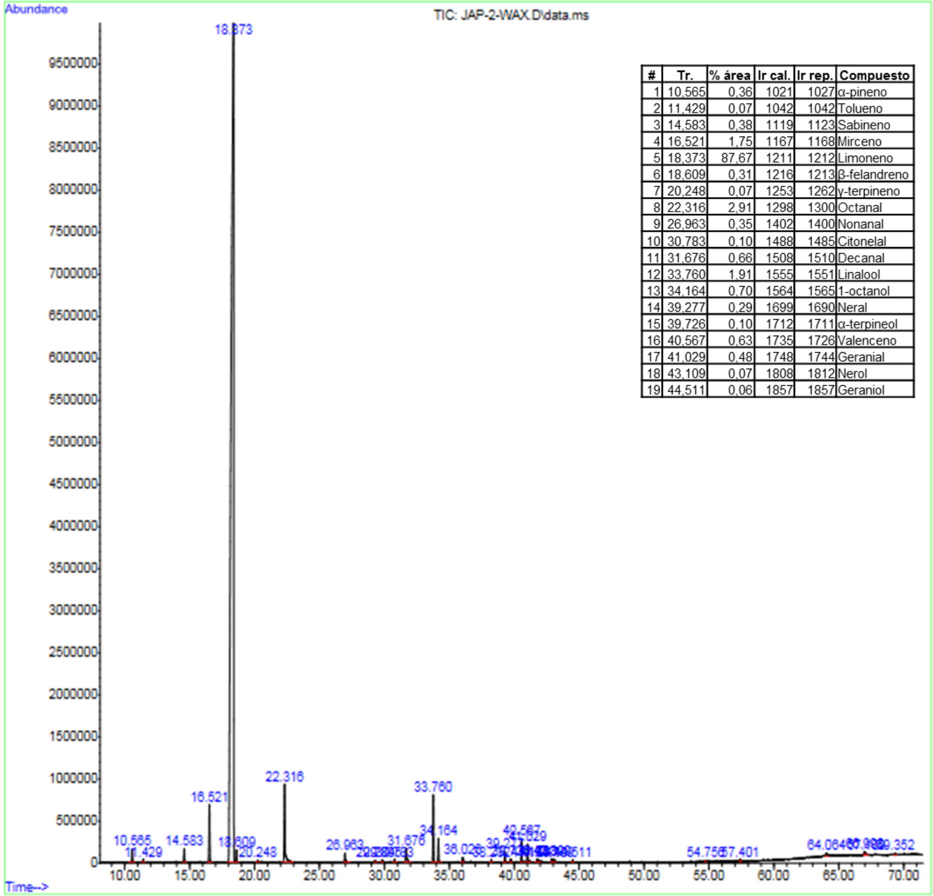

#	Tr.	% área	Ir cal.	Ir rep.	Compuesto
1	10,565	0,36	1021	1027	α-pineno
2	11,429	0,07	1042	1042	Tolueno
3	14,583	0,38	1119	1123	Sabineno
4	16,521	1,75	1167	1168	Mirceno
5	18,373	87,67	1211	1212	Limoneno
6	18,609	0,31	1216	1213	β-felandreno
7	20,248	0,07	1253	1262	γ-terpineno
8	22,316	2,91	1298	1300	Octanal
9	26,963	0,35	1402	1400	Nonanal
10	30,783	0,10	1488	1485	Citonelal
11	31,676	0,66	1508	1510	Decanal
12	33,760	1,91	1555	1551	Linalool
13	34,164	0,70	1564	1565	1-octanol
14	39,277	0,29	1699	1690	Neral
15	39,726	0,10	1712	1711	α-terpineol
16	40,567	0,63	1735	1726	Valenceno
17	41,029	0,48	1748	1744	Geranial
18	43,109	0,07	1808	1812	Nerol
19	44,511	0,06	1857	1857	Geraniol

Chromatographic profiles obtained for the essential oil of *C. album* leaves and steams

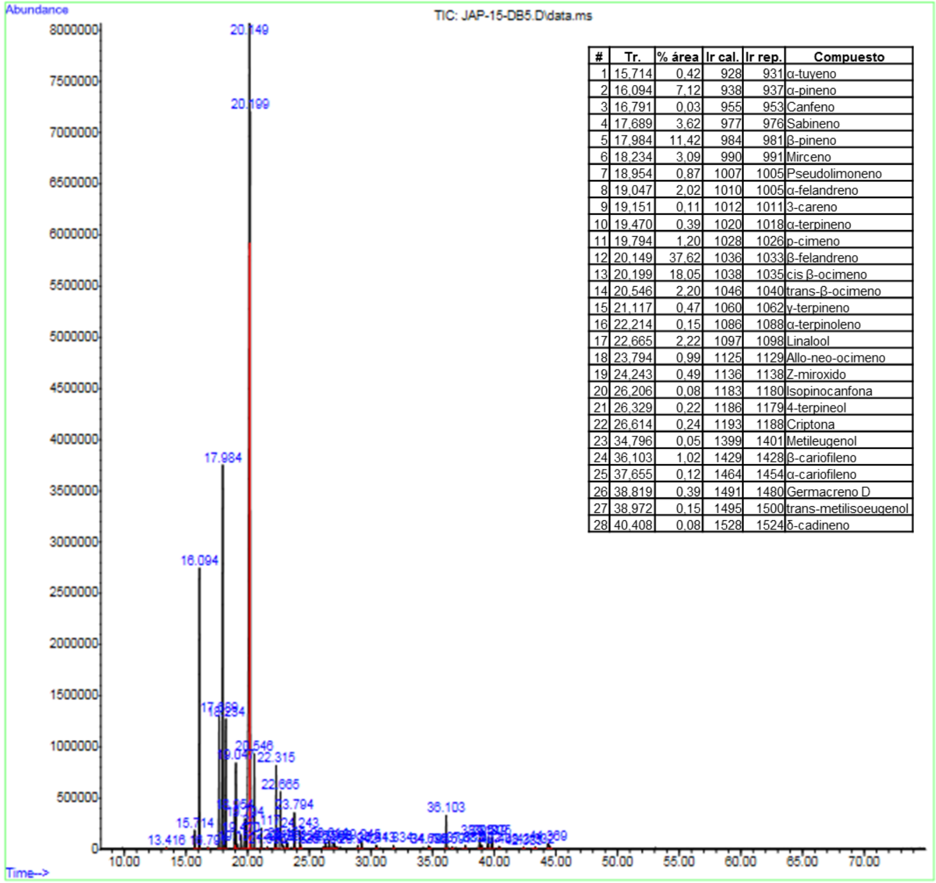

#	Tr.	% área	Ir cal.	Ir rep.	Compuesto
1	15,714	0,42	928	931	α-tuyeno
2	16,094	7,12	938	937	α-pineno
3	16,791	0,03	955	953	Canfeno
4	17,689	3,62	977	976	Sabineno
5	17,984	11,42	984	981	β-pineno
6	18,234	3,09	990	991	Mirceno
7	18,954	0,87	1007	1005	Pseudolimoneno
8	19,047	2,02	1010	1005	α-felandreno
9	19,151	0,11	1012	1011	3-careno
10	19,470	0,39	1020	1018	α-terpineno
11	19,794	1,20	1028	1026	p-cimeno
12	20,149	37,62	1036	1033	β-felandreno
13	20,199	18,05	1038	1035	cis β-ocimeno
14	20,546	2,20	1046	1040	trans-β-ocimeno
15	21,117	0,47	1060	1062	γ-terpineno
16	22,214	0,15	1086	1088	α-terpinoleno
17	22,665	2,22	1097	1098	Linalool
18	23,794	0,99	1125	1129	Allo-neo-ocimeno
19	24,243	0,49	1136	1138	Z-miroxido
20	26,206	0,08	1183	1180	Isopinocanfona
21	26,329	0,22	1186	1179	4-terpineol
22	26,614	0,24	1193	1188	Criptona
23	34,796	0,05	1399	1401	Metileugenol
24	36,103	1,02	1429	1428	β-cariofileno
25	37,655	0,12	1464	1454	α-cariofileno
26	38,819	0,39	1491	1480	Germacreno D
27	38,972	0,15	1495	1500	trans-metilisoeugenol
28	40,408	0,08	1528	1524	δ-cadineno

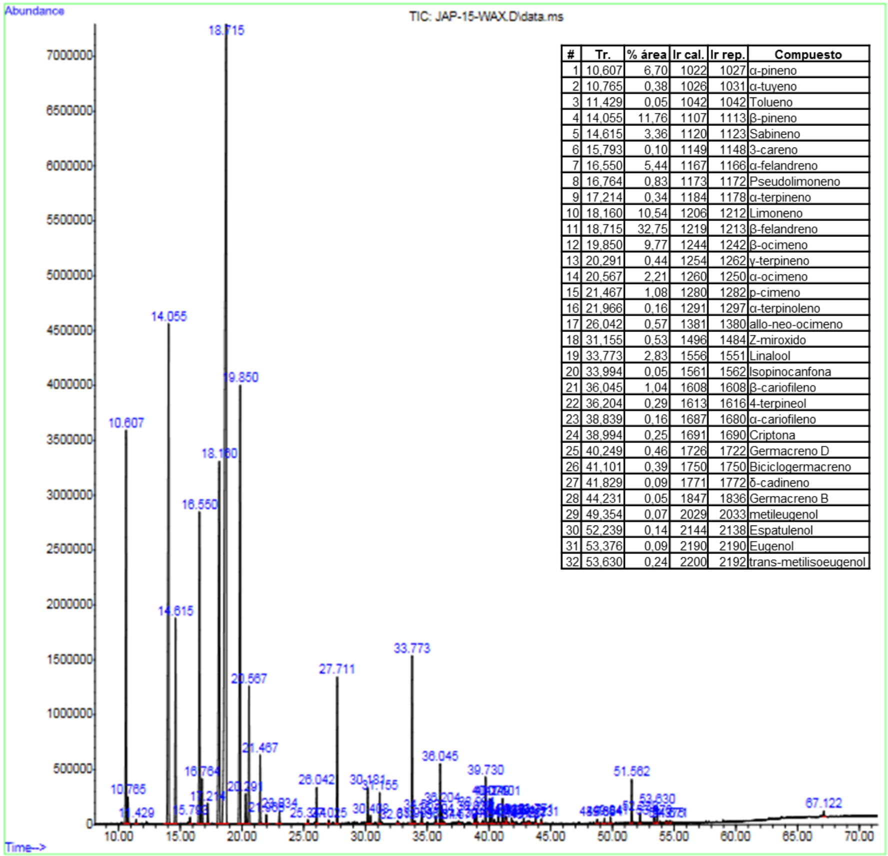

#	Tr.	% área	Ir cal.	Ir rep.	Compuesto
1	10,607	6,70	1022	1027	α-pineno
2	10,765	0,38	1026	1031	α-tuyeno
3	11,429	0,05	1042	1042	Tolueno
4	14,055	11,76	1107	1113	β-pineno
5	14,615	3,36	1120	1123	Sabineno
6	15,793	0,10	1149	1148	3-careno
7	16,550	5,44	1167	1166	α-felandreno
8	16,764	0,83	1173	1172	Pseudolimoneno
9	17,214	0,34	1184	1178	α-terpineno
10	18,160	10,54	1206	1212	Limoneno
11	18,715	32,75	1219	1213	β-felandreno
12	19,850	9,77	1244	1242	β-ocimeno
13	20,291	0,44	1254	1262	γ-terpineno
14	20,567	2,21	1260	1250	α-ocimeno
15	21,467	1,08	1280	1282	p-cimeno
16	21,966	0,16	1291	1297	α-terpinoleno
17	26,042	0,57	1381	1380	allo-neo-ocimeno
18	31,155	0,53	1496	1484	Z-miroxido
19	33,773	2,83	1556	1551	Linalool
20	33,994	0,05	1561	1562	Isopinocanfona
21	36,045	1,04	1608	1608	β-cariofileno
22	36,204	0,29	1613	1616	4-terpineol
23	38,839	0,16	1687	1680	α-cariofileno
24	38,994	0,25	1691	1690	Criptona
25	40,249	0,46	1726	1722	Germacreno D
26	41,101	0,39	1750	1750	Biciclogermacreno
27	41,829	0,09	1771	1772	δ-cadineno
28	44,231	0,05	1847	1836	Germacreno B
29	49,354	0,07	2029	2033	metileugenol
30	52,239	0,14	2144	2138	Espatulenol
31	53,376	0,09	2190	2190	Eugenol
32	53,630	0,24	2200	2192	trans-metilisoeugenol

Chromatographic profiles obtained for the essential oil of *L. alba* leaves and flowers

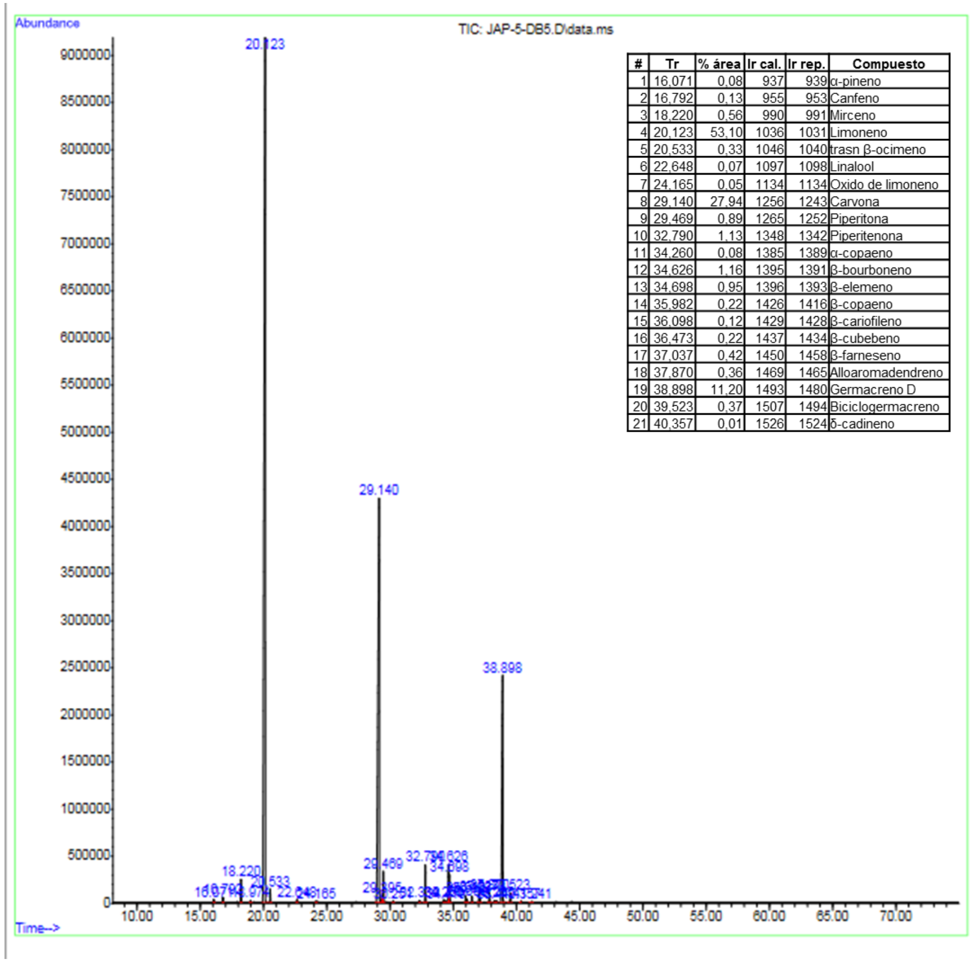

#	Tr	% área	Ir cal.	Ir rep.	Compuesto
1	16,071	0,08	937	939	α-pineno
2	16,792	0,13	955	953	Canfeno
3	18,220	0,56	990	991	Mirceno
4	20,123	53,10	1036	1031	Limoneno
5	20,533	0,33	1046	1040	trasn β-ocimeno
6	22,648	0,07	1097	1098	Linalool
7	24,165	0,05	1134	1134	Oxido de limoneno
8	29,140	27,94	1256	1243	Carvona
9	29,469	0,89	1265	1252	Piperitona
10	32,790	1,13	1348	1342	Piperitenona
11	34,260	0,08	1385	1389	α-copaeno
12	34,626	1,16	1395	1391	β-bourboneno
13	34,698	0,95	1396	1393	β-elemeno
14	35,982	0,22	1426	1416	β-copaeno
15	36,098	0,12	1429	1428	β-cariofileno
16	36,473	0,22	1437	1434	β-cubebeno
17	37,037	0,42	1450	1458	β-farneseno
18	37,870	0,36	1469	1465	Alloaromadendreno
19	38,898	11,20	1493	1480	Germacreno D
20	39,523	0,37	1507	1494	Biciclogermacreno
21	40,357	0,01	1526	1524	δ-cadineno

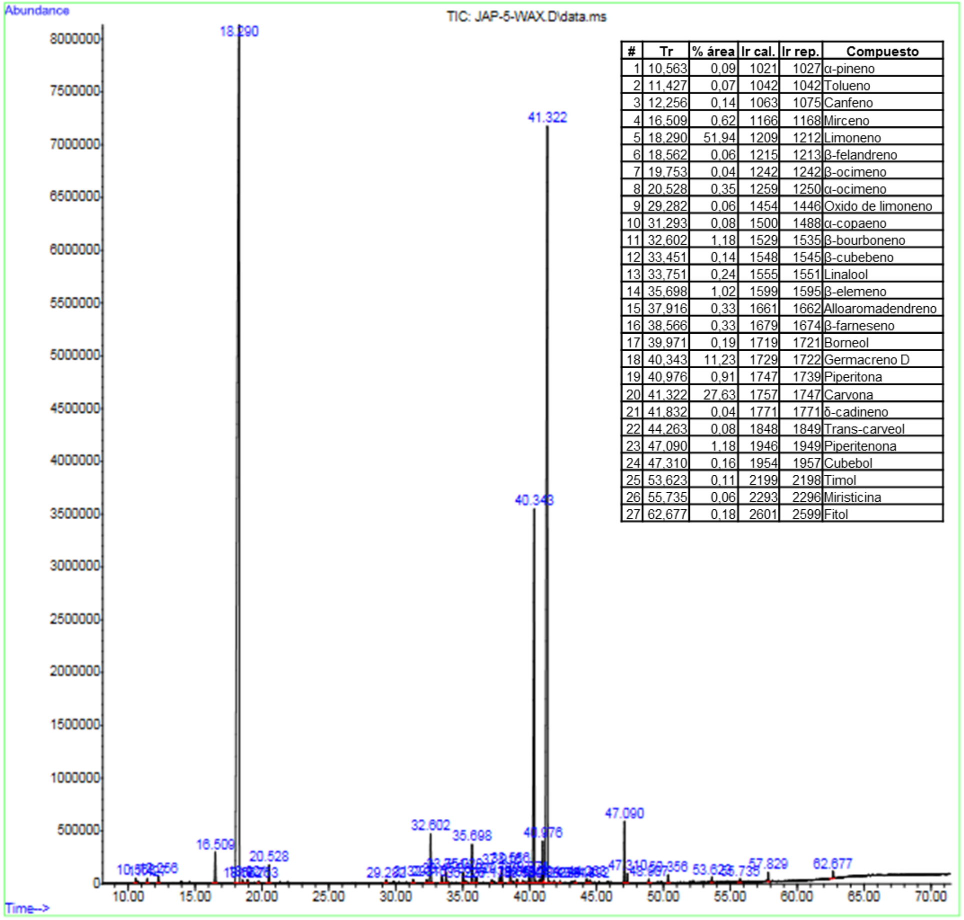

#	Tr	% área	Ir cal.	Ir rep.	Compuesto
1	10,563	0,09	1021	1027	α-pineno
2	11,427	0,07	1042	1042	Tolueno
3	12,256	0,14	1063	1075	Canfeno
4	16,509	0,62	1166	1168	Mirceno
5	18,290	51,94	1209	1212	Limoneno
6	18,562	0,06	1215	1213	β-felandreno
7	19,753	0,04	1242	1242	β-ocimeno
8	20,528	0,35	1259	1250	α-ocimeno
9	29,282	0,06	1454	1446	Oxido de limoneno
10	31,293	0,08	1500	1488	α-copaeno
11	32,602	1,18	1529	1535	β-bourboneno
12	33,451	0,14	1548	1545	β-cubebeno
13	33,751	0,24	1555	1551	Linalool
14	35,698	1,02	1599	1595	β-elemeno
15	37,916	0,33	1661	1662	Alloaromadendreno
16	38,566	0,33	1679	1674	β-farneseno
17	39,971	0,19	1719	1721	Borneol
18	40,343	11,23	1729	1722	Germacreno D
19	40,976	0,91	1747	1739	Piperitona
20	41,322	27,63	1757	1747	Carvona
21	41,832	0,04	1771	1771	δ-cadineno
22	44,263	0,08	1848	1849	Trans-carveol
23	47,090	1,18	1946	1949	Piperitenona
24	47,310	0,16	1954	1957	Cubebol
25	53,623	0,11	2199	2198	Timol
26	55,735	0,06	2293	2296	Miristicina
27	62,677	0,18	2601	2599	Fitol

Chromatographic profiles obtained for the essential oil of *L. origanoides* leaves

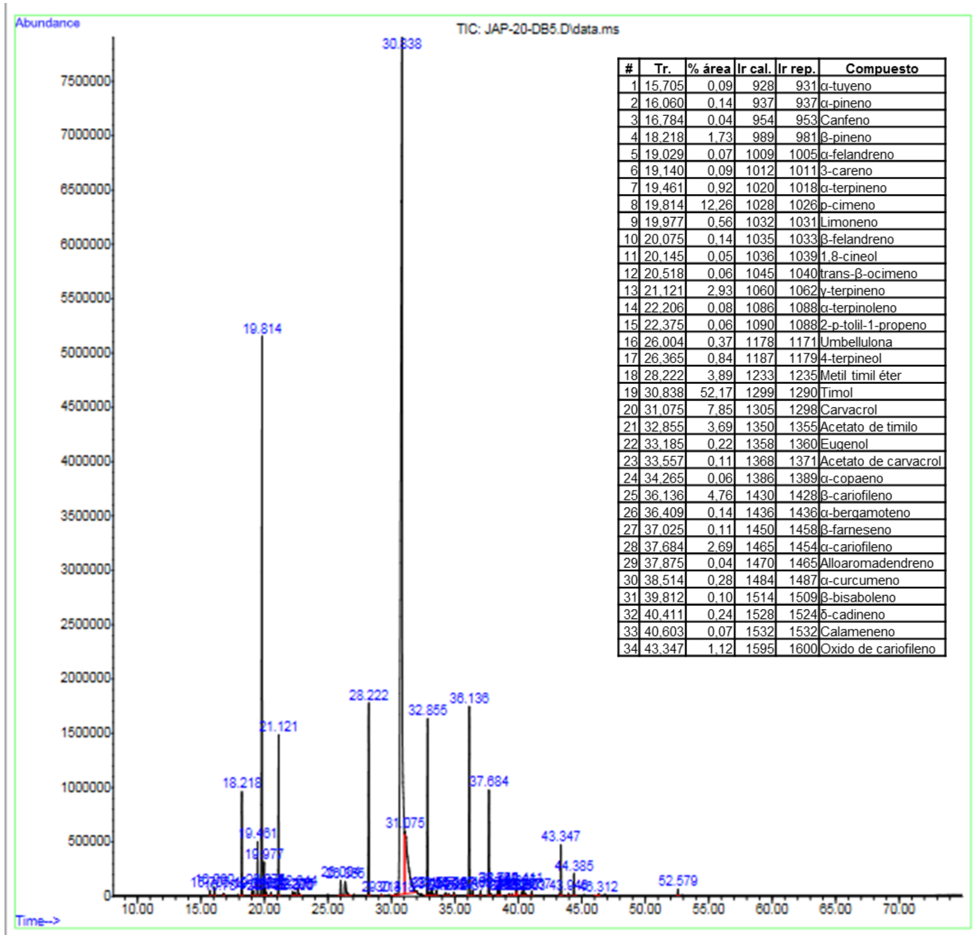

#	Tr.	% área	Ir cal.	Ir rep.	Compuesto
1	15,705	0,09	928	931	α-tuyeno
2	16,060	0,14	937	937	α-pineno
3	16,784	0,04	954	953	Canfeno
4	18,218	1,73	989	981	β-pineno
5	19,029	0,07	1009	1005	α-felandreno
6	19,140	0,09	1012	1011	3-careno
7	19,461	0,92	1020	1018	α-terpineno
8	19,814	12,26	1028	1026	p-cimeno
9	19,977	0,56	1032	1031	Limoneno
10	20,075	0,14	1035	1033	β-felandreno
11	20,145	0,05	1036	1039	1,8-cineol
12	20,518	0,06	1045	1040	trans-β-ocimeno
13	21,121	2,93	1060	1062	γ-terpineno
14	22,206	0,08	1086	1088	α-terpinoleno
15	22,375	0,06	1090	1088	2-p-tolil-1-propeno
16	26,004	0,37	1178	1171	Umbellulona
17	26,365	0,84	1187	1179	4-terpineol
18	28,222	3,89	1233	1235	Metil timil éter
19	30,838	52,17	1299	1290	Timol
20	31,075	7,85	1305	1298	Carvacrol
21	32,855	3,69	1350	1355	Acetato de timilo
22	33,185	0,22	1358	1360	Eugenol
23	33,557	0,11	1368	1371	Acetato de carvacrol
24	34,265	0,06	1386	1389	α-copaeno
25	36,136	4,76	1430	1428	β-cariofileno
26	36,409	0,14	1436	1436	α-bergamoteno
27	37,025	0,11	1450	1458	β-farneseno
28	37,684	2,69	1465	1454	α-cariofileno
29	37,875	0,04	1470	1465	Alloaromadendreno
30	38,514	0,28	1484	1487	α-curcumeno
31	39,812	0,10	1514	1509	β-bisaboleno
32	40,411	0,24	1528	1524	δ-cadineno
33	40,603	0,07	1532	1532	Calameneno
34	43,347	1,12	1595	1600	Oxido de cariofileno

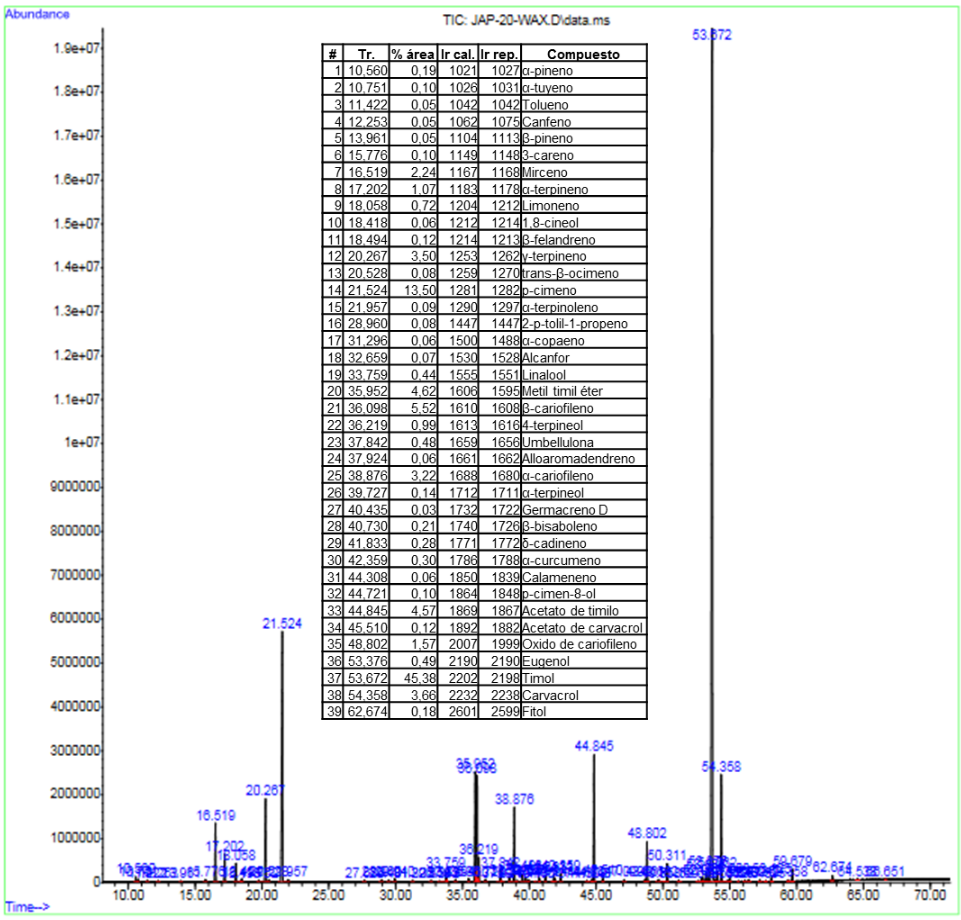

#	Tr.	% área	Ir cal.	Ir rep.	Compuesto
1	10,560	0,19	1021	1027	α-pineno
2	10,751	0,10	1026	1031	α-tuyeno
3	11,422	0,05	1042	1042	Tolueno
4	12,253	0,05	1062	1075	Canfeno
5	13,961	0,05	1104	1113	β-pineno
6	15,776	0,10	1149	1148	3-careno
7	16,519	2,24	1167	1168	Mirceno
8	17,202	1,07	1183	1178	α-terpineno
9	18,058	0,72	1204	1212	Limoneno
10	18,418	0,06	1212	1214	1,8-cineol
11	18,494	0,12	1214	1213	β-felandreno
12	20,267	3,50	1253	1262	γ-terpineno
13	20,528	0,08	1259	1270	trans-β-ocimeno
14	21,524	13,50	1281	1282	p-cimeno
15	21,957	0,09	1290	1297	α-terpinoleno
16	28,960	0,08	1447	1447	2-p-tolil-1-propeno
17	31,296	0,06	1500	1488	α-copaeno
18	32,659	0,07	1530	1528	Alcanfor
19	33,759	0,44	1555	1551	Linalool
20	35,952	4,62	1606	1595	Metil timil éter
21	36,098	5,52	1610	1608	β-cariofileno
22	36,219	0,99	1613	1616	4-terpineol
23	37,842	0,48	1659	1656	Umbellulona
24	37,924	0,06	1661	1662	Alloaromadendreno
25	38,876	3,22	1688	1680	α-cariofileno
26	39,727	0,14	1712	1711	α-terpineol
27	40,435	0,03	1732	1722	Germacreno D
28	40,730	0,21	1740	1726	β-bisaboleno
29	41,833	0,28	1771	1772	δ-cadineno
30	42,359	0,30	1786	1788	α-curcumeno
31	44,308	0,06	1850	1839	Calameneno
32	44,721	0,10	1864	1848	p-cimen-8-ol
33	44,845	4,57	1869	1867	Acetato de timilo
34	45,510	0,12	1892	1882	Acetato de carvacrol
35	48,802	1,57	2007	1999	Oxido de cariofileno
36	53,376	0,49	2190	2190	Eugenol
37	53,672	45,38	2202	2198	Timol
38	54,358	3,66	2232	2238	Carvacrol
39	62,674	0,18	2601	2599	Fitol
