# Supplementary material for: Effects of Essential Oils from 24 Plant Species on Sitophilus zeamais Motsch (Coleoptera, Curculionidae)
Source: Insects. 2021 Jun 8;12(6):532. doi: 10.3390/insects12060532 (PMC8230103; doi:10.3390/insects12060532)
Supplement: Supplementary file 1 [file insects-12-00532-s001.zip › insects-1160839-s/insects-1160839-supplementaryWITH CORRECTIONS.pdf]

Supplementary material

Table S1. Sample information of the 45 species plants worked.

Family	Specie	Common name	# Collection	Obtaining or collection site	State and used parts
Anacardiaceae	*Schinus molle**	Falso pimiento	Nd	Universidad Nacional de Colombia (Bogotá)	Fresh fruits
Annonaceae	*Xilopia discreta*	-	COL518691	Barrancabermeja (Santander)	Dry Fruits
Apiaceae	*Foeniculum vulgare**	Hinojo	Nd	Market place (Bogotá)	Fresh aerial parts
Asteraceae	*Ambrosia cumanensis*	Altamisa	COL595396	San Mateo (Boyacá)	Fresh aerial parts
	*Artemisia drancunculus**	Estragón	Nd	Market place (Bogotá)	Fresh aerial parts
Cupressaceae	*Cupressus sempervirens*	Ciprés	COL595397	Universidad Nacional de Colombia (Bogotá)	Fresh leaves
Hypericaceae	*Hypericum mexicanum*	Chite	JBB10659	Usme (Bogotá)	Fresh aerial parts
	Hypericum myricariifolium	Chite	JBB00350	Usme (Bogotá)	Aerial parts
	Hypericum juniperinum	Chite, Guardarocío	JBB00348	Usme (Bogotá)	Aerial parts
Illiciaceae	*Illicium verum*	Anís estrellado	COL595400	Market place (Bogotá)	Dry fruits
Lamiaceae	*Rosmarinus officinalis*	Romero	COL595398	Market place (Bogotá)	Fresh leaves
	Satureja viminea	Menta	COL596919	Botanical garden (Bogotá)	Fresh aerial parts
	Minthostachys septentrionalis	-	COL596918	Botanical garden (Bogotá)	Fresh aerial parts
	Ocimum basilicum	Albahaca	COL595395	Market place (Bogotá)	Fresh leaves
	Lavandula stoechas	Lavanda	JBB03053	Botanical garden (Bogotá)	Fresh aerial parts
Lauraceae	*Aniba puchury-minor*	-	COL544559	Fusagasugá (Cundinamarca)	Fresh leaves
	Beilschmiedia costaricensis	-	COL563246	La Vega (Cundinamarca)	Fresh leaves
	Aniba robusta	-	COL563245	La Vega (Cundinamarca)	Fresh leaves
	Ocotea sp.*	Cascarillo	Nd	Arauca (Arauca)	Fresh leaves
	Nectandra acutifolia	-	COL566240	Fusagasugá (Cundinamarca)	Fresh leaves
	Cinnamomum triplinerve	-	COL548028	Nocaima (Cundinamarca)	Fresh leaves
	Ocotea longifolia	Laurel	COL563466	Lerida (Tolima)	Fresh leaves

Myristicaceae	*Virola carinata*	-	COL563244	Granada (Meta)	Fresh leaves
Myrtaceae	*Eucalyptus* sp.**	Eucalipto	COL596913	Usme (Bogotá)	Fresh leaves
	Syzygium aromaticum	Clavo	Nd	Market place (Bogotá)	Fresh leaves
Piperaceae	*Piper nubigenum*	Cordoncillo	COL595897	San Mateo (Boyacá)	Fresh aerial parts
	Piper aduncum	Cordoncillo	COL593727	La sierra (Cundinamarca)	Fresh leaves
	Piper holtonii	Cordoncillo	COL593726	El Colegio (Cundinamarca)	Fresh aerial parts
	Piper bogotense	Cordoncillo	COL511095	Sopó (Cundinamarca)	Fresh leaves
	Piper eriopodon	Cordoncillo	COL595898	Santa bárbara (Santander)	Fresh leaves
	Piper marginatum	Cordoncillo	COL591820	El Colegio (Cundinamarca)	Fresh leaves
	Piper imperiale	Cordoncillo	COL519826	Duitama (Boyacá)	Fresh leaves
	Piper pesaresanum	Cordoncillo	COL553307	Santa Bárbara (Santander)	Fresh leaves
	Piper el-metanum	Cordoncillo	COL553310	Acacias (meta)	Fresh leaves
	Piper cf. *asperiusculum*	Cordoncillo	COL579924	San Mateo (Boyacá)	Fresh inflorescences
	Piper pertomentellum	Cordoncillo	COL579920	San Mateo (Boyacá)	Fresh inflorescences
	Piper aequale	Cordoncillo	COL510622	Nocaima (Cundinamarca)	Fresh leaves
	Peperomia sp.**	-	COL526963	Acacias (Meta)	Fresh leaves
Poaceae	*Cymbopogon nardus**	Citronela	Nd	Market place Manizales (Caldas)	Fresh leaves
	Cymbopogon citratus	Limonaria	COL595393	Fusagasugá (Cundinamarca)	Fresh leaves
Rutaceae	*Coleonema album*	Diosme	COL526771	Market place (Bogotá)	Fresh aerial parts
	*Citrus × sinensis**	Naranja	Nd	Market place (Bogotá)	Fresh shell
Verbenaceae	*Lippia origanoides**	Orégano de monte	Nd	Cañon del Chicamocha (Santander)	Fresh leaves
	Lippia alba	Pronto alivio	COL595394	San Mateo (Boyacá)	Fresh aerial parts
Zingiberaceae	*Curcuma longa**	Cúrcuma	Nd	Market place (Bogotá)	Fry roots

* Common species were taxonomically determined in a herbarium, but were not deposited in the collection.
** The analyzed specimen only allowed taxonomic characterization to be performed until the genus.

Table S2.

Chemical composition of the EOs of 24 spices with potential insecticidal activity.

N°	Components	RI a (DB-5MS)	RI b (HP-INNOWax)	*Ambrosia cumanensis*	*Citrus × sinensis*	*Coleonema album*	*Cupressus semperoirens*	*Cymbopogon citratus*	*Cymbopogon nardus*	*Eucalyptus sp.*	*Hypericum mexicanum*	*Hypericum myricariifolium*	*Illicium verum*	*Lavandula stoechas*	*Lippia alba*	*Lippia origanoides*	*Minthostachys septentrionalis*	*Ocimum basilicum*	*Ocotea sp.*	*Piper aduncum*	*Piper cf. asperiusculum*	*Piper el-metanum*	*Piper nubigenum*	*Piper pertomentellum*	*Rosmarinus officinalis*	*Satureja viminea*	*Xylopia discreta*
1	Octane	797	-	-	-	-	-	-	-	-	0.36	-	-	-	-	-	-	-	-	-	-	-	-	-	-	-	-
2	2-Hexenal	851	-	-	-	-	-	-	-	-	-	-	-	-	-	-	-	-	-	-	-	-	-	-	-	0,04	-
3	2-Methyloctane	866	-	-	-	-	-	-	-	-	-	0.54	-	-	-	-	-	-	-	-	-	-	-	-	-	-	-
4	Nonane	904	-	-	-	-	-	-	-	-	53.08	1.59	-	-	-	-	-	-	-	-	-	-	-	-	-	-	-
5	Tricyclene	927	1009	-	-	-	0.3	-	-	-	0	-	-	0.06	-	-	-	-	0.04	-	-	-	-	-	0.25	-	-
6	α-Tuyene	928	1026	-	-	0.42	1.95	-	-	-	0.08	0.05	-	-	-	0.09	-	-	-	-	0.05	0.88	0.09	-	0.35	-	0,38
7	α-Pinene	939	1023	0.66	0.29	7.12	16.89	-	-	6.6	25.28	45.52	0.26	2.8	0.08	0.14	0.69	0.03	24.01	6.14	2	8.48	0.9	0.81	23.2	0,18	24,71
8	α-Fenchene	955	1055	-	-	-	0.45	-	-	-	-	0.05	-	0.1	-	-	-	-	0.48	0.08	-	-	-	-	-	-	-
9	Camphene	956	1064	0.43	-	0.03	0.54	-	-	0.03	-	0.28	-	1.22	0.13	0.04	-	-	1.79	0.23	0.09	0.07	-	-	8.71	-	0,69
10	2,4-Tuyadiene	958	1124	-	-	-	-	-	-	-	-	-	-	-	-	-	-	-	-	-	-	-	-	-	0.09	-	-
11	3-Methylnonane	971	976	-	-	-	-	-	-	-	1.24	0.49	-	-	-	-	-	-	-	-	-	-	-	-	-	-	-
12	Sabinene	976	1119	0.45	0.29	3.62	9.39	-	-	-	0.32	-	-	0.19	-	-	0.33	0.05	-	-	-	0.46	0.23	0.18	0.16	0,12	5,08
13	β-Pinene	983	1105	5.33	-	11.42	0.46	-	-	0.11	3.6	2.86	0.02	0.54	-	1.73	0.8	0.09	3.19	0.34	1.22	0.27	2.01	1.55	4.87	0,26	36,04
14	Sulcatone	984	1347	-	-	-	-	0.42	-	-	-	-	-	-	-	-	-	-	-	-	-	-	-	-	-	-	-
15	Myrcene	990	1172	1	1.37	3.09	3.75	2.04	0.08	0.26	1.11	1.67	0.08	0.25	0.56	-	-	0.8	0.6	1.82	0.67	1.4	0.42	0.22	1.13	-	1,07
16	3-Octanol	996	1397	-	-	-	-	-	-	-	-	-	-	-	-	-	0.09	-	-	-	-	-	-	-	-	-	-
17	Decane	1000	1000	-	-	-	-	-	-	-	0.13	-	-	-	-	-	-	-	-	-	-	-	-	-	-	-	-
18	Octanal	1004	1298	-	2.78	-	-	-	-	-	-	-	-	-	-	-	-	-	-	-	-	-	-	-	-	-	-
19	Pseudolimonene	1007	1174	-	-	0.87	-	-	-	-	-	-	-	-	-	-	-	-	-	-	-	-	-	-	-	-	-
20	α-Phellandrene	1010	1167	-	-	2.02	0.51	-	-	-	-	-	0.08	0.07	-	0.07	-	-	-	2.48	0.86	43.47	0.28	-	2.89	-	1,71
21	Δ-3-Carene	1012	1149	-	-	0.11	11.93	-	-	-	-	-	0.22	3.99	-	0.09	-	-	-	0.2	0.03	0.24	0.07	-	0.05	-	0,03
22	Isocineole	1017	1180	-	-	-	-	-	-	-	-	-	-	-	-	-	-	-	0.23	-	-	-	-	-	-	-	-
23	α-Terpinene	1020	1184	-	-	0.39	4.85	-	-	-	-	0.07	0.05	0.09	-	0.92	-	-	0.16	0.36	0.11	0.21	5.1	-	0.69	-	-
24	*o*-Cimene	1022	1277	-	-	-	-	-	-	-	-	-	-	0.2	-	-	-	-	-	-	-	-	-	-	-	-	-
25	*p*-Cimene	1028	1279	-	-	1.2	1.9	-	-	0.76	0.14	0.55	0.1	0.5	-	12.26	-	-	1.9	4.73	0.06	4.96	0.55	-	0.55	-	3,4
26	Limonene	1033	1205	1.48	91.22	-	6.58	-	5.94	6.5	0.28	1.9	2.33	1.73	53.1	0.56	1.84	0.07	5.93	6.59	2.82	19.36	0.61	0.24	3.72	1,05	3,04
27	cis-β-*o*Cimene	1035	1242	-	-	18.05	-	-	-	-	-	1.12	-	1.15	-	-	-	-	-	-	-	-	-	28.49	-	-	-
28	β-Phellandrene	1036	1215	0.35	0.03	37.62	7.07	-	0.04	-	0.05	-	0.2	-	-	-	0.04	0.03	-	4.87	1.13	7.78	0.14	-	-	-	1,56
29	1,8-Cineole	1038	1217	-	-	-	-	-	-	66.65	-	-	0.27	17.12	-	-	-	0.26	1.74	-	6.3	-	-	-	23.22	0,14	10,06
30	Phenylacetaldehyde	1045	-	-	-	-	-	-	-	-	-	-	-	-	-	-	-	-	-	-	0	-	-	-	0.04	-	-
31	trasn β-*o*Cimene	1046	1259	0.45	-	2.2	0.05	-	-	-	-	0.22	-	-	0.33	0.06	-	0.55	-	0.11	0.94	0.06	-	21.02	-	-	-
32	Bergamal	1052	1363	-	-	-	-	-	0.08	-	-	-	-	-	-	-	-	-	-	-	-	-	-	-	-	-	-
33	γ-Terpinene	1060	1253	-	0.05	0.47	5.72	-	-	0.06	-	0.23	0.09	0.09	-	2.93	-	-	0.21	0.39	0.2	0.24	9.81	-	1.59	-	-
34	2-Methyldecane	1062	1051	-	-	-	-	-	-	-	0.07	-	-	-	-	-	-	-	-	-	-	-	-	-	-	-	-
35	*p*-Ment-3,8-diene	1072	1273	-	-	-	-	-	-	-	-	-	-	-	-	-	0.04	-	-	-	-	-	-	-	-	2,03	-
36	Linalool oxide	1072	1448	-	-	-	-	-	-	-	-	-	-	0.2	-	-	-	-	-	-	-	-	-	-	-	-	-

37	α-Terpinolene	1087	1291	-	-	0.15	6.58	-	0.08	0.03	-	0.21	-	0.33	-	0.08	0.07	-	3.67	0.09	0.07	2.87	2.34	-	0.82	0,22	0,06
38	2-*p*-Tolyl-1-propene	1091	1447	-	-	-	0.12	-	-	-	-	-	0.02	-	-	0.06	-	-	-	-	-	-	-	-	-	-	-
39	Fenchone	1094	1409	-	-	-	-	-	-	-	-	-	-	27.77	-	-	-	-	0.22	-	-	-	-	-	-	-	-
40	Linalool	1095	1555	-	1.17	2.22	-	1.35	-	0.04	-	-	-	3.55	0.07	-	-	2.17	-	22.43	-	-	-	0.26	0.09	0,64	-
41	Undecane	1097	1094	-	-	-	-	-	-	-	2.72	0.65	-	-	-	-	-	-	-	-	-	-	-	-	-	-	-
42	Nonanal	1101	1402	-	0.48	-	-	-	-	-	-	-	-	-	-	-	-	-	-	-	-	-	-	-	-	-	-
43	3-Octyl acetate	1113	1344	-	-	-	-	-	-	-	-	-	-	-	-	-	1.72	-	-	-	-	-	-	-	-	-	-
44	Fenchol	1122	1593	-	-	-	-	-	-	-	-	-	-	-	-	-	-	-	1.85	-	-	-	-	-	-	-	-
45	Chrysanthenone	1123	1522	-	-	-	-	-	-	-	-	-	-	-	-	-	-	-	-	-	-	-	-	-	0.09	-	-
46	Allo-neo-ocimene	1125	1381	-	-	0.99	-	-	-	-	-	-	-	-	-	-	-	-	-	-	-	-	-	1.2	-	-	-
47	α-Canpholenal	1128	1503	-	-	-	-	-	-	-	-	-	-	0.21	-	-	-	-	-	-	-	-	-	-	-	-	-
48	Limonene oxide	1134	1454	-	-	-	-	-	-	-	-	-	-	-	0.05	-	-	-	-	-	-	-	-	-	-	-	-
49	*Z*-Myroxide	1136	1496	-	-	0.49	-	-	-	-	-	-	-	-	-	-	-	-	-	-	-	-	-	-	-	-	-
50	1-Terpineol	1137	1584	-	-	-	-	-	-	-	-	-	-	-	-	-	-	-	0.4	-	-	-	-	-	-	-	-
51	Citronellal	1150	1488	-	0.1	-	-	-	39.41	-	-	-	-	-	-	-	-	-	-	-	-	-	-	-	-	-	-
52	Camphor	1155	1532	0.41	-	-	0.21	-	-	-	-	-	-	27.99	-	-	-	-	1.36	0.1	0.08	-	-	-	13.19	-	-
53	*p*-Menth-3-en-8-ol	1158	1623	-	-	-	-	-	-	-	-	-	-	-	-	-	-	-	-	-	-	-	-	-	-	45,39	-
54	Menthone	1161	1474	-	-	-	-	-	-	-	-	-	-	-	-	-	8.17	-	-	-	-	-	-	-	-	-	-
55	Menthofuran	1168	1495	-	-	-	-	-	-	-	-	-	-	-	-	-	0.05	-	-	-	-	-	-	-	-	-	-
56	Pinocarvone	1168	1583	-	-	-	-	-	-	-	-	-	-	-	-	-	-	-	-	-	-	-	-	-	0.1	-	0,07
57	Isomenthone	1171	1502	-	-	-	-	-	-	-	-	-	-	-	-	-	2.01	-	-	-	-	-	-	-	-	0,13	-
58	Umbellulone	1173	1659	-	-	-	0.72	-	-	-	-	-	-	-	-	0.37	-	-	-	-	-	-	-	-	-	-	-
59	Isoborneol	1177	1685	-	-	-	-	-	-	-	-	-	-	-	-	-	-	-	0.12	-	-	-	-	-	-	-	-
60	Dihydrocarvone	1180	-	-	-	-	-	-	-	-	-	-	-	-	-	-	0.4	-	-	-	-	-	-	-	-	-	-
61	Isopulegone	1181	1587	-	-	-	-	-	-	-	-	-	-	-	-	-	-	-	-	-	-	-	-	-	-	0,43	-
62	Isopinocamphone	1184	1560	-	-	0.08	-	-	-	-	-	-	-	-	-	-	-	-	-	-	-	-	-	-	0.08	0,05	-
63	Terpinen-4-ol	1186	1613	-	-	0.22	0.79	-	-	0.31	-	-	0.15	0.48	-	0.84	-	-	1.09	0.3	-	-	-	-	0.39	-	0,06
64	*p*-Cymene-8-ol	1191	1858	-	-	-	-	-	-	-	-	-	-	0.31	-	-	-	-	0.27	-	-	-	-	-	-	-	-
65	Criptone	1195	1691	-	-	0.24	-	-	-	-	-	-	-	-	-	-	-	-	-	-	-	-	-	-	-	-	0,07
66	α-Terpineol	1201	1711	-	-	-	-	-	-	1.91	-	-	-	0.68	-	-	-	-	44.29	-	-	-	-	-	0.92	0,16	-
67	Estragole	1202	1696	-	-	-	-	-	-	-	-	-	1.16	-	-	-	-	81.78	-	-	-	-	-	-	-	-	-
68	Decanal	1207	1508	-	0.63	-	-	-	0.21	-	-	0.54	-	-	-	-	-	-	-	-	-	-	0.17	-	-	-	-
69	Verbenone	1216	1728	-	-	-	-	-	-	-	-	-	-	-	-	-	-	-	-	-	-	-	-	-	1.21	-	-
70	Fenchyl acetate	1225	1477	-	-	-	-	-	-	-	-	-	-	-	-	-	-	0.06	0.99	-	-	-	-	-	-	-	-
71	Thymyl methyl ether	1233	1617	-	-	-	0.06	-	-	-	-	-	-	-	-	3.89	-	-	-	-	-	-	-	-	-	-	-
72	Neral	1243	1699	-	0.28	-	-	30.75	0.47	-	-	-	-	-	-	-	-	-	-	-	-	-	-	-	-	-	-
73	Pulegone	1252	1670	-	-	-	-	-	-	-	-	-	-	-	-	-	41.85	-	-	-	-	-	-	-	-	38,61	-
74	Carvone	1256	1753	-	-	-	-	-	0.06	-	-	-	-	-	27.94	-	-	-	-	-	-	-	-	-	-	-	-
75	*cis*-Anethole	1260	1774	-	-	-	-	-	-	-	-	-	0.32	-	-	-	-	-	-	-	-	-	-	-	-	-	-
76	Piperitone	1265	1747	-	-	-	-	-	-	-	-	-	-	-	0.89	-	0.16	-	-	45.46	4.57	-	-	-	-	-	-
77	*p*-Anisaldehyde	1266	2053	-	-	-	-	-	-	-	-	-	0.34	-	-	-	-	-	-	-	-	-	-	-	-	-	-
78	Geranial	1272	1748	-	0.42	-	-	44.62	2.08	-	-	-	-	-	-	-	-	-	-	-	-	-	-	-	-	-	-
79	Isopulegol acetate	1277	-	-	-	-	-	-	-	-	-	-	-	-	-	-	-	-	-	-	-	-	-	-	-	0,04	-
80	Menthyl acetate	1279	1541	-	-	-	-	-	-	-	-	-	-	-	-	-	1.84	-	-	-	-	-	-	-	-	-	-
81	Lavandulyl acetate	1285	1616	-	-	-	-	-	-	-	-	-	-	0.57	-	-	-	-	-	-	-	-	-	-	-	-	-
82	Bornyl acetate	1294	1592	0.11	-	-	0.3	-	-	-	-	-	-	-	-	-	-	-	1.62	-	-	-	0.06	-	3.1	-	0,16
83	Anethole	1295	1846	-	-	-	-	-	-	-	-	-	93.4	-	-	-	0.76	0.07	-	-	-	-	-	-	-	-	-
84	2-Undecanone	1296	-	-	-	-	-	-	-	-	-	-	-	-	-	-	-	-	-	-	-	-	-	0.07	-	-	-
85	Safrole	1299	1898	-	-	-	-	-	-	-	-	-	-	-	-	-	-	-	-	-	0.03	-	0.28	-	-	-	-
86	Thymol	1299	2199	-	-	-	-	-	-	-	-	-	-	-	-	52.17	-	-	-	-	-	-	-	-	-	-	-
87	Terpinen-4-ol acetate	1304	1628	-	-	-	0.99	-	-	-	-	-	-	-	-	-	-	-	-	-	-	-	-	-	-	-	-
88	Carvacrole	1305	2232	-	-	-	-	-	-	-	-	-	-	-	-	7.85	-	-	-	-	-	-	-	-	-	-	-
89	Nerolic acid	1320	2309	-	-	-	-	0.5	-	-	-	-	-	-	-	-	-	-	-	-	-	-	-	-	-	-	-

90	Myrtenyl acetate	1331	1705	-	-	-	-	-	-	-	-	-	-	-	-	-	-	-	-	-	-	-	-	-	-	0,13	-
91	1,5,5-Trimethyl-6-methylene-Cyclohexene	1342	1488	-	-	-	-	-	-	-	-	-	-	-	-	-	0.69	-	-	-	-	-	-	-	-	-	-
92	δ-Elemene	1345	1477	-	-	-	-	-	-	-	-	-	-	-	-	-	-	-	-	-	-	-	4.02	1.18	-	-	-
93	Piperitenone	1348	1944	-	-	-	-	-	-	-	-	-	-	-	1.13	-	0.27	-	-	-	-	-	-	-	-	0,36	-
94	Citronellyl acetate	1350	1675	-	-	-	-	-	10.04	-	-	-	-	-	-	-	-	-	-	-	-	-	0.1	-	-	-	-
95	Thymol acetate	1350	1869	-	-	-	-	-	-	-	-	-	-	-	-	3.69	-	-	-	-	-	-	-	-	-	-	-
96	α-Cubebene	1353	1463	-	-	-	-	-	-	-	-	-	-	-	-	-	-	-	-	-	0.1	-	-	0.39	-	-	0,08
97	Terpinyl acetate	1354	1714	-	-	-	2.81	-	-	12.2	-	-	-	-	-	-	-	-	1.83	-	-	-	-	-	-	-	0,09
98	Geranic acid	1355	2354	-	-	-	-	3.17	-	-	-	-	-	-	-	-	-	-	-	-	-	-	-	-	-	-	-
99	Eugenol	1358	2189	-	-	-	-	-	1.14	-	-	-	-	-	-	0.22	-	0.33	-	-	-	-	-	-	-	-	-
100	Carvacrol acetate	1368	1892	-	-	-	-	-	-	-	-	-	-	-	-	0.11	-	-	-	-	-	-	-	-	-	-	-
101	Piperitenone oxide	1369	1984	-	-	-	-	-	-	-	-	-	-	-	-	-	2.86	-	-	-	-	-	-	-	-	-	-
102	Geranyl acetate	1377	1768	-	-	-	-	0.52	15.93	0.08	-	-	-	0.05	-	-	-	-	-	-	-	-	-	-	-	-	-
103	Methyl anisate	1379	2119	-	-	-	-	-	-	-	-	-	0.04	-	-	-	-	-	-	-	-	-	-	-	-	-	-
104	α-Ylengene	1381	-	-	-	-	-	-	-	-	-	-	-	-	-	-	-	-	-	-	0.1	-	-	-	-	-	-
105	Ciclosativene	1381	1489	-	-	-	-	-	-	-	0.37	-	-	-	-	-	-	-	-	-	-	-	-	-	-	-	0,08
106	α-Copaene	1385	1500	0.67	-	-	-	-	-	-	0.41	-	-	-	0.08	0.06	0.1	0.07	-	0.11	1.01	0.28	0.15	0.53	-	0,34	0,34
107	Methyl cinnamate	1391	2104	-	-	-	-	-	-	-	-	-	-	-	-	-	-	5.1	-	-	-	-	-	-	-	-	-
108	β-Bourbonene	1395	1529	-	-	-	-	-	0.15	-	-	-	-	-	1.16	-	0.03	-	-	-	-	-	-	-	-	0,08	-
109	β-Elemene	1396	1599	-	-	-	-	-	-	-	-	-	-	-	-	-	-	0.98	-	-	-	-	-	1.31	-	-	-
110	β-Cubebene	1396	1548	-	-	-	-	-	-	-	0.22	-	-	-	0.95	-	-	-	-	-	0.1	0.1	0.71	-	-	-	-
111	Methyl eugenol	1399	2029	-	-	0.05	-	-	-	-	-	-	-	-	-	-	-	-	-	-	-	-	0.07	-	0.13	-	-
112	Cyperene	1415	1538	-	-	-	-	-	-	-	-	-	-	-	-	-	-	-	-	-	-	0.14	-	-	-	-	0,04
113	α-Gurjunene	1416	1540	0.27	-	-	-	-	-	0.33	-	-	-	-	-	-	-	-	-	-	-	-	-	-	-	0,04	-
114	α-Santalene	1424	1581	-	-	-	-	-	-	-	0.4	0.38	-	-	-	-	-	-	-	-	-	-	-	-	-	-	-
115	β-Copaene	1426	1500	-	-	-	-	-	-	-	-	-	-	-	0.22	-	-	-	-	-	-	-	-	-	-	-	-
116	β-Caryophyllene	1430	1610	1.99	-	1.02	0.26	-	-	-	2.01	13.59	-	-	0.12	4.76	16.51	-	-	-	0.5	0.17	7.09	4.78	6.47	2,17	0,14
117	γ-Elemene	1433	1652	-	-	-	-	-	-	-	-	-	-	-	-	-	-	-	-	-	-	-	-	0.2	-	-	-
118	α-Bergamotene	1436	1594	-	-	-	-	-	-	-	-	0.86	0.09	-	-	0.14	-	1.01	-	-	-	-	-	-	-	-	0,39
119	α-Guaiene	1440	-	-	-	-	-	-	-	-	-	-	-	-	-	-	-	0.25	-	-	-	-	-	-	-	-	-
120	β-Gurjunene	1440	1602	-	-	-	-	-	-	0.08	-	-	-	-	-	-	-	-	-	-	-	-	-	-	-	-	-
121	Aromadendrene	1447	1619	-	-	-	-	-	-	1.06	-	-	-	-	-	-	-	-	-	-	-	-	0.05	-	-	-	-
122	β-Farnesene	1450	1679	0.7	-	-	-	-	-	-	-	0.26	-	-	0.42	0.11	-	0.23	-	-	-	-	-	-	-	-	-
123	α-Caryophyllene	1465	1687	0.7	-	0.12	0.53	-	-	-	0.46	4.69	-	-	-	2.69	2.84	0.19	-	-	0.04	-	0.79	0.66	0.8	0,11	-
124	Alloaromadendrene	1469	1661	-	-	-	-	-	-	0.36	-	-	-	-	0.36	0.04	0.18	-	-	-	0.05	0.31	0.68	0.14	-	2,38	0,08
125	γ-Selinene	1481	1692	-	-	-	-	-	-	-	-	0.64	-	-	-	-	-	-	-	-	-	0.21	-	-	-	-	-
126	γ-Curcumene	1484	1711	34.11	-	-	-	-	-	-	-	-	-	-	-	-	-	-	-	-	-	-	-	-	-	-	-
127	α-Curcumene	1485	1786	2.45	-	-	-	-	-	-	-	1.46	-	-	-	0.28	-	-	-	-	-	-	-	-	-	-	-
128	Germacreno D	1491	1726	17.25	-	0.39	-	-	2.7	-	-	-	-	0.67	11.2	-	0.53	1.67	-	0.08	0.41	0.75	1.85	26.88	-	0,39	1,05
129	Valencene	1491	1735	-	0.57	-	-	-	-	-	-	-	-	-	-	-	-	-	-	-	-	-	-	-	-	-	-
130	trans-Methyl isoeguenol	1495	2200	-	-	0.15	-	-	-	-	-	-	0.05	-	-	-	-	-	-	-	-	-	-	-	-	-	-
131	δ-Selinene	1495	1709	-	-	-	-	-	-	-	-	-	-	-	-	-	-	-	-	-	-	0.6	-	-	-	-	-
132	1,10-Calamenene oxide	1495	1903	-	-	-	-	-	-	-	-	-	-	1.03	-	-	-	-	-	-	-	-	-	-	-	-	-
133	Eremofilene	1498	-	-	-	-	-	-	-	-	0.06	-	-	-	-	-	-	-	-	-	-	-	-	-	-	-	-
134	α-Zingiberene	1499	1734	-	-	-	-	-	-	-	-	1.34	-	-	-	-	-	-	-	-	-	-	-	-	-	-	-
135	β-Selinene	1501	1749	-	-	-	-	-	-	-	0.32	3.09	-	-	-	-	-	-	-	-	-	0.37	-	-	-	-	-
136	Viridiflorene	1502	1727	-	-	-	-	-	-	0.37	-	-	-	-	-	-	-	-	-	-	-	-	-	-	-	-	-
137	epi-Bicyclosesquiphellandrene	1504	1731	-	-	-	-	-	-	-	0.1	-	-	-	-	-	-	-	-	-	-	-	-	-	-	-	-

138	cis α-Bisabolene	1505	1741	-	-	-	-	-	-	-	-	2.4	-	-	-	-	-	-	-	-	-	-	-	0.21	-	-	1,29
139	α-Farnesene	1506	1759	-	-	-	-	-	-	-	-	-	-	-	-	-	-	0.55	-	-	-	1.43	-	-	-	-	-
140	Bicyclogermacrene	1507	1750	16.79	-	-	-	-	-	-	-	-	-	-	0.37	-	8.67	-	-	-	0.14	-	5.78	2.14	-	0,66	-
141	α-Muurolene	1507	1739	-	-	-	-	-	0.31	-	-	-	-	-	-	-	-	-	-	-	-	-	-	-	-	-	-
142	α-Selinene	1508	1750	-	-	-	-	-	-	-	0.51	2.5	-	-	-	-	-	-	-	-	-	-	-	-	-	-	-
143	β-Bisabolene	1514	1740	1.01	-	-	-	-	-	-	-	0.85	-	-	-	0.1	-	-	-	-	-	0.13	-	-	0.1	-	0,21
144	γ-Bisabolene	1519	1769	-	-	-	-	-	-	-	-	0.24	-	-	-	-	-	-	-	-	-	-	-	-	-	-	-
145	δ-Cadinene	1527	1771	-	-	0.08	0.09	-	1.2	-	0.46	0.07	-	0.59	0.01	0.24	0.1	0.06	-	0.2	0.33	0.18	0.12	0.59	-	0,53	-
146	Myristicin	1532	2293	1.16	-	-	-	-	-	-	-	-	-	-	-	-	-	-	-	-	38.12	-	36.68	-	-	-	-
147	Calamenene	1532	1849	-	-	-	-	-	-	-	0.14	-	-	0.1	-	0.07	-	-	-	0.08	-	-	-	-	-	-	0,06
148	β-Sesquiphellandrene	1532	1781	0.4	-	-	-	-	-	-	-	0.37	-	-	-	-	-	-	-	-	-	-	-	-	0.05	-	-
149	7-Epi-α-selinene	1534	1779	-	-	-	-	-	-	-	-	0.94	-	-	-	-	-	-	-	-	-	0.13	-	-	-	-	-
150	Elemicin	1548	2246	-	-	-	-	-	-	-	-	-	-	-	-	-	-	-	-	-	0.16	-	0.25	-	-	-	-
151	trans α-Bisabolene	1553	1784	-	-	-	-	-	-	-	-	-	-	-	-	-	-	-	-	-	-	-	-	-	-	-	1,79
152	α-Calacorene	1553	1938	-	-	-	-	-	-	-	-	-	-	-	-	-	-	-	-	0.19	-	-	-	-	-	-	-
153	Selina-3,7(11)-diene	1555	1793	-	-	-	-	-	-	-	-	-	-	0.1	-	-	-	-	-	-	-	0.07	-	-	-	-	-
154	Elemol	1562	2097	-	-	-	-	-	5.15	-	-	-	-	-	-	-	-	-	-	-	-	-	-	-	-	-	-
155	Germacrene B	1573	1847	-	-	-	-	-	-	-	-	-	-	-	-	-	-	-	-	-	-	-	-	0.37	-	-	-
156	Caryophyllene Oxide	1600	2006	-	-	-	-	-	-	-	-	-	-	-	-	1.12	0.14	-	-	-	-	0.27	-	-	0.4	0,96	-
157	Spathulenol	1601	2144	-	-	-	-	-	-	-	-	-	-	-	-	-	-	-	-	-	-	-	-	-	-	-	3,43
158	Globulol	1601	2096	-	-	-	-	-	-	0.92	-	-	-	-	-	-	-	-	-	-	-	-	-	-	-	-	-
159	Dillapiole	1622	2386	-	-	-	-	-	-	-	-	-	-	-	-	-	-	-	-	0.07	35.38	-	-	-	0.05	1,06	-
160	Isoelemecin	1637	2420	-	-	-	-	-	-	-	-	-	-	-	-	-	-	-	-	-	-	-	0.07	-	-	-	-
161	Apiole	1662	2506	-	-	-	-	-	-	-	-	-	-	-	-	-	-	-	0.26	0.08	-	-	11.83	-	-	-	-
162	Cembrene A	1834	2207	-	-	-	-	-	-	-	1.38	1.13	-	-	-	-	-	-	-	-	-	-	-	-	-	-	-
163	13-Isopimaradiene	1868	2286	-	-	-	0.15	-	-	-	-	-	-	-	-	-	-	-	-	-	-	-	-	-	-	-	-
164	Isophyllocladene	1874	2282	-	-	-	0.11	-	-	-	-	-	-	-	-	-	-	-	-	-	-	-	-	-	-	-	-
165	Abietatriene	1936	2521	-	-	-	0.59	-	-	-	-	-	-	-	-	-	-	-	-	-	-	-	-	-	-	-	-
166	Abietadiene	1969	2492	-	-	-	8.35	-	-	-	-	-	-	-	-	-	-	-	-	-	-	-	-	-	-	-	-
	Oxygenated monoterpenes (%)			**10,51**	**0,52**	0.52	1.97	3.25	5.82	80.91	69.21	81.19	-	-	1.9	78.93	30.08	65.25	57.61	2.82	56.01	68.29	10.95	-	0.16	0.26	42.39
	Non-oxygenated monoterpenes (%)			**77,77**	**10,15**	10.15	93.25	89.77	78.92	2.04	6.14	14.35	30.86	54.73	3.43	13.31	54.2	18.97	3.81	1.62	41.98	28.43	10.25	90.75	22.55	53.71	49.07
	Total monoterpenes (%)			**88,28**	**10,67**	10.67	95.22	93.02	84.74	82.95	75.35	95.54	30.86	54.73	5.33	92.24	84.28	81.22	61.42	4.44	97.99	96.72	21.2	90.75	22.71	53.97	91.46
	Oxygenated sesquiterpenes (%)			**3,43**	**-**	-	-	-	-	-	3.86	0.92	-	-	-	1.03	-	1.12	0.14	-	-	-	-	0.27	-	-	0.4
	Non-oxygenated sesquiterpenes (%)			**5,55**	**76,34**	76.34	0.57	1.61	0.88	-	5.65	2.2	5.46	33.68	0.09	1.46	14.89	8.49	28.96	5.01	-	0.66	2.78	4.87	21.24	39.38	7.42
	Total sesquiterpenes (%)			**8,98**	**76,34**	76.34	0.57	1.61	0.88	-	9.51	3.12	5.46	33.68	0.09	2.49	14.89	9.61	29.1	5.01	-	0.66	2.78	5.14	21.24	39.38	7.82
	Diterpenes (%)			**-**	**-**	-	-	-	9.2	-	-	-	1.38	1.13	-	-	-	-	-	-	-	-	-	-	-	-	-
	Phenylpropanes (%)			**-**	**1,16**	1.16	-	0.2	0.18	-	-	-	-	-	93.85	-	-	3.95	0.76	86.95	0.26	0.15	73.69	-	49.18	-	0.18
	Others (%)			**-**	**-**	-	3.89	-	-	0.42	0.21	-	61.89	3.81	-	-	-	-	2.5	-	-	-	-	-	0.17	0.07	0.04
	**Total identified (%)**			**97,26**	**88,17**	88.17	99.68	94.83	95	83.37	85.07	98.66	99.59	93.35	99.27	94.73	99.17	97.78	93.78	96.4	98.25	97.53	97.67	95.89	93.3	93.42	99.5
	**Density (25 °C) (g/mL)**			**0,885**	**0,86**	0.86	0.845	0.837	0.871	0.944	0.852	0.903	0.775	0.852	0.968	0.872	0.853	0.895	0.964	0.96	0.879	0.895	1.035	0.862	0.988	0.886	0.913
	**Refractive index (27 °C)**			**1,477**	**1,494**	1.494	1.468	1.479	1.481	1.492	1.467	1.46	1.422	1.475	1.553	1.449	1.483	1.507	1.47	1.516	1.476	1.476	1.517	1.478	1.511	1.497	1.467

RI: Retention index (**a**) DB-5MS not polar column, (**b**) HP-INNOWax polar column

Table S3.

Repellent effect of the 24 active EOs on adults of *S. zeamais* * .

Essential Oil specie	Repellency percentage ± SD							Essential Oil specie	Repellency percentage ± SD						
	Concentration (µL/L air)	2 hours		6 hours		24 hours			Concentration (µL/L air)	2 hours		6 hours		24 hours	
	6.2	66.7 ± 6.7	IV	76.7 ± 12.0	IV	53.3 ± 6.7	III		6,2	60.0 ± 5.8	III	73.3 ± 3.3	IV	-3.3 ± 3.3	0
	10.0	66.7 ± 6.7	IV	60.0 ± 5.8	V	80.0 ± 0.0	IV		10,0	60.0 ± 10.0	III	53.3 ± 6.7	III	33.3 ± 6.7	II
A. cumanensis	14.1	76.7 ± 12.0	IV	80.0 ± 10.0	IV	76.7 ± 8.8	IV	*Lippia* sp.	14,1	50.0 ± 5.8	III	56.7 ± 6.7	III	80.0 ± 5.8	IV
	18.0	43.3 ± 3.3	III	70.0 ± 5.8	IV	76.7 ± 6.7	IV		18,0	73.3 ± 3.3	IV	63.3 ± 8.8	IV	83.3 ± 6.7	V
	22.7	73.3 ± 8.8	IV	80.0 ± 10.0	IV	76.7 ± 6.7	IV		22,7	70.0 ± 5.8	IV	86.7 ± 3.3	V	83.3 ± 6.7	V
	6.2	90.0 ± 5.8	V	83.3 ± 8.8	V	50.0 ± 5.8	III		6,2	80.0 ± 15.3	IV	76.7 ± 3.3	IV	56.7 ± 6.7	III
	10.0	86.7 ± 3.3	V	80.0 ± 10.0	IV	50.0 ± 10.0	III		10,0	63.3 ± 8.8	IV	80.0 ± 10.0	IV	76.7 ± 6.7	IV
C. album	14.1	76.7 ± 12.0	IV	73.3 ± 3.3	IV	80.0 ± 5.8	IV	*M. septentrionalis*	14,1	70.0 ± 5.8	IV	93.3 ± 6.7	V	80.0 ± 5.8	IV
	18.0	80.0 ± 5.8	IV	93.3 ± 3.3	V	90.0 ± 5.8	V		18,0	76.7 ± 8.8	IV	83.3 ± 6.7	V	83.3 ± 6.7	V
	22.7	66.7 ± 8.8	II	80.0 ± 5.8	IV	76.7 ± 8.8	IV		22,7	70.0 ± 5.8	IV	76.7 ± 6.7	IV	70.0 ± 5.8	IV
	6.2	73.3 ± 3.3	IV	80.0 ± 5.8	IV	86.7 ± 3.3	V		6,2	73.3 ± 6.7	IV	83.3 ± 8.8	V	40.0 ± 5.8	II
	10.0	70.0 ± 0.0	IV	83.3 ± 3.3	V	93.3 ± 6.7	V		10,0	53.3 ± 8.8	III	83.3 ± 12.0	V	46.7 ± 8.8	III
C. citratus	14.1	73.3 ± 6.7	IV	73.3 ± 6.7	IV	90.0 ± 5.8	V	*O. basilicum*	14,1	46.7 ± 3.3	III	73.3 ± 14.5	IV	40.0 ± 10.0	II
	18.0	70.0 ± 10.0	IV	73.3 ± 12.0	IV	73.3 ±6.7	IV		18,0	50.0 ± 5.8	III	86.7 ± 6.7	V	66.7 ± 6.7	IV
	22.7	63.3 ± 12.0	IV	63.3 ± 6.7	IV	80.0 ± 5.8	IV		22,7	50.0 ± 5.8	III	70.0 ± 5.8	IV	73.3 ± 6.7	IV
	6.2	100.0 ± 0.0	V	96.7 ± 3.3	V	90.0 ± 5.8	V		6,2	76.7 ± 3.3	IV	93.3 ± 3.3	V	96.7 ± 3.3	V
	10.0	93.3 ± 6.7	V	70.0 ± 5.8	IV	86.7 ± 3.3	V		10,0	66.7 ± 3.3	IV	86.7 ± 3.3	V	96.7 ± 3.3	V
C. nardus	14.1	70.0 ± 10.0	IV	66.7 ± 8.8	IV	80.0 ± 10.0	IV	*Ocotea* sp.	14,1	96.7 ± 3.3	V	100.0 ± 0.0	V	96.7 ± 3.3	V
	18.0	93.3 ± 6.7	V	100.0 ± 0.0	V	93.3 ± 3.3	V		18,0	100.0 ± 0.0	V	100.0 ± 0.0	V	96.7 ± 3.3	V
	22.7	80.0 ± 5.8	IV	100.0 ± 0.0	V	90.0 ± 0.0	V		22,7	100.0 ± 0.0	V	100.0 ± 0.0	V	93.3 ± 3.3	V
	6.2	73.3 ± 6.7	IV	3.3 ± 3.3	IV	30.0 ± 5.8	II		6,2	70.0 ± 10.0	IV	90.0 ± 0.0	V	93.3 ± 3.3	V
C. sempervirens	10.0	73.3 ± 3.3	IV	76.7 ± 3.3	IV	46.7 ± 46.7	III	*P. aduncum*	10,0	96.7 ± 3.3	V	96.7 ± 3.3	V	76.7 ± 6.7	IV
	14.1	76.7 ± 8.8	IV	76.7 ± 3.3	IV	73.3 ± 6.7	IV		14,1	86.7 ± 3.3	V	83.3 ± 8.8	V	80.0 ± 5.8	IV
	18.0	73.3 ± 6.7	IV	76.7 ± 3.3	IV	73.3 ± 8.8	IV		18,0	86.7 ± 8.8	V	73.3 ± 6.7	IV	83.3 ± 6.7	V

	22.7	73.3 ± 6.7	IV	73.3 ± 6.7	IV	93.3 ± 3.3	IV		22,7	83.3 ± 3.3	V	90.0 ± 5.8	V	93.3 ± 3.3	V
	6.2	93.3 ± 3.3	V	73.3 ± 6.7	IV	86.7 ± 8.8	V		6,2	86.7 ± 3.3	V	86.7 ± 6.7	V	73.3 ± 17.6	IV
	10.0	83.3 ± 8.8	V	90.0 ± 5.8	V	86.7 ± 8.8	V		10,0	76.7 ± 6.7	IV	53.3 ± 17.6	III	80.0 ± 10.0	IV
Citrus × sinensis	14.1	53.3 ± 8.8	III	66.7 ± 6.7	V	36.7 ± 6.7	II	*P. asperiusculum*	14,1	53.3 ± 6.7	III	70.0 ± 5.8	IV	66.7 ± 6.7	IV
	18.0	83.3 ± 8.8	V	90.0 ± 5.8	V	93.3 ± 6.7	V		18,0	23.3 ± 3.3	II	56.7 ± 0.0	III	16.7 ± 6.7	I
	22.7	83.3 ± 3.3	V	90.0 ± 10.0	V	93.3 ± 6.7	V		22,7	46.7 ± 6.7	III	36.7 ± 12.0	III	33.3 ± 3.3	III
	6.2	53.3 ± 3.3	III	26.7 ± 3.3	II	53.3 ± 6.7	III		6,2	50.0 ± 5.8	II	63.3 ± 3.3	IV	53.3 ± 12.0	III
	10.0	20.0 ± 5.8	II	26.7 ± 3.3	II	33.3 ± 8.8	II		10,0	76.7 ± 3.3	IV	70.0 ± 5.8	IV	96.7 ± 3.3	V
Eucalyptus sp.	14.1	30.0 ± 5.8	II	46.7 ± 3.3	III	53.3 ± 6.7	III	*P. el-metanum*	14,1	73.3 ± 6.7	IV	76.7 ± 6.7	IV	100.0 ± 0.0	V
	18.0	53.3 ± 8.8	III	73.3 ± 3.3	IV	93.3 ± 3.3	V		18,0	80.0 ± 5.8	IV	96.7 ± 3.3	V	93.3 ± 3.3	V
	22.7	63.3 ± 8.8	IV	80.0 ± 5.8	IV	83.3 ± 8.8	V		22,7	66.7 ± 6.7	IV	83.3 ± 3.3	V	83.3 ± 8.8	V
	6.2	90.0 ± 5.8	V	80.0 ± 10.0	IV	73.3 ± 6.7	IV		6,2	66.7 ± 8.8	IV	33.3 ± 3.3	II	40.0 ± 5.8	II
	10.0	80.0 ± 11.5	IV	86.7 ± 6.7	V	73.3 ± 6.7	IV		10,0	43.3 ± 8.8	III	66.7 ± 8.8	IV	93.3 ± 6.7	V
H. mexicanum	14.1	83.3 ± 6.7	V	90.0 ± 10.0	V	90.0 ± 0.0	V	*P. nubigenum*	14,1	50.0 ± 5.8	III	60.0 ± 11.5	III	73.3 ± 6.7	IV
	18.0	90.0 ± 5.8	V	80.0 ± 10.0	IV	93.3 ± 3.3	V		18,0	63.3 ± 3.3	IV	80.0 ± 11.5	IV	83.3 ± 3.3	V
	22.7	90.0 ± 10.0	V	90.0 ± 10.0	V	86.7 ± 3.3	V		22,7	43.3 ± 6.7	III	86.7 ± 6.7	V	96.7 ± 3.3	V
	6.2	93.3 ± 3.3	V	93.3 ± 6.7	V	90.0 ± 5.8	V		6,2	70.0 ± 10.0	IV	70.0 ± 5.8	IV	86.7 ± 3.3	V
	10.0	93.3 ± 3.3	V	86.7 ± 8.8	V	80.0 ± 0.0	IV		10,0	80.0 ± 5.8	IV	76.7 ± 3.3	IV	96.7 ± 3.3	V
H. myricariifolium	14.1	96.7 ± 3.3	V	96.7 ± 3.3	V	83.3 ± 8.8	V	*P. pertomentellum*	14,1	76.7 ± 3.3	IV	73.3 ± 3.3	IV	96.7 ± 3.3	V
	18.0	90.0 ± 5.8	V	93.3 ± 3.3	V	96.7 ± 3.3	V		18,0	80.0 ± 0.0	IV	80.0 ± 5.8	IV	96.7 ± 3.3	V
	22.7	100.0 ± 0.0	V	100.0 ± 0.0	V	90.0 ± 0.0	V		22,7	86.7 ± 6.7	V	93.3 ± 6.7	V	96.7 ± 3.3	V
	6.2	30.0 ± 10.0	II	16.7 ± 3.3	I	56.7 ± 8.8	III		6,2	80.0 ± 0.0	IV	63.3 ± 8.8	IV	-16.7 ± 3.3	0
	10.0	43.3 ± 17.6	III	26.7 ± 8.8	II	20.0 ± 5.8	I		10,0	80.0 ± 5.8	IV	86.7 ± 3.3	V	40.0 ± 5.8	II
I. verum	14.1	33.3 ± 12.0	II	26.7 ± 6.7	II	20.0 ± 10.0	I	*R. officinalis*	14,1	100.0 ± 0.0	V	100.0 ± 0.0	V	73.3 ± 6.7	IV
	18.0	23.3 ± 12.0	II	46.7 ± 8.8	III	20.0 ± 10.0	I		18,0	93.3 ± 6.7	V	80.0 ± 5.8	V	93.3 ± 6.7	V
	22.7	83.3 ± 8.8	IV	63.3 ± 6.7	III	46.7 ± 3.3	III		22,7	93.3 ± 3.3	V	90.0 ± 0.0	V	93.3 ± 6.7	V
	6.2	-3.3 ± 3.3	0	3.3 ± 6.7	I	73.3 ± 6.7	IV		6,2	73.3 ± 12.0	IV	63.3 ± 6.7	IV	53.3 ± 3.3	III
	10.0	23.3 ± 3.3	II	16.7 ± 3.3	I	70.0 ± 5.8	IV		10,0	60.0 ± 10.0	III	73.3 ± 3.3	IV	70.0 ± 5.8	IV
L. alba	14.1	33.3 ± 3.3	II	36.7 ± 3.3	II	33.3 ± 6.7	II	*S. viminea*	14,1	73.3 ± 6.7	IV	80.0 ± 0.0	IV	76.7 ± 3.3	IV
	18.0	33.3 ± 5.8	II	40.0 ± 0.0	II	33.3 ± 6.7	II		18,0	63.3 ± 6.7	IV	73.3 ± 8.8	IV	70.0 ± 11.5	IV
	22.7	50.0 ± 5.8	III	46.7 ± 3.3	III	40.0 ± 5.8	II		22,7	76.7 ± 6.7	IV	73.3 ± 6.7	IV	70.0 ± 5.8	IV

	6.2	83.3 ± 3.3	V	93.3 ± 3.3	V	80.0 ± 10.0	IV		6,2	66.7 ± 8.8	IV	56.7 ± 8.8	III	80.0 ± 10.0	IV
	10.0	93.3 ± 6.7	V	100.0 ± 0.0	V	96.7 ± 3.3	V		10,0	56.7 ± 8.8	III	76.7 ± 6.7	IV	83.3 ± 8.8	V
L. stoechas	14.1	86.7 ± 8.8	V	86.7 ± 8.8	V	83.3 ± 3.3	V	*X. discreta*	14,1	66.7 ± 6.7	IV	63.3 ± 6.7	IV	83.3 ± 8.8	V
	18.0	93.3 ± 3.3	V	96.7 ± 3.3	V	80.0 ± 5.8	IV		18,0	80.0 ± 5.8	IV	80.0 ± 5.8	IV	93.3 ± 3.3	V
	22.7	90.0 ± 5.8	V	93.3 ± 6.7	V	90.0 ± 10.0	V		22,7	76.7 ± 6.7	IV	80.0 ± 5.8	IV	93.3 ± 3.3	V
								Negative control	-	0.0 ± 5.8	0	1.0 ± 3.3	0	-0.8 ± 3.3	0

*% repellency = class: <0.1 % = 0; 0.1 - 20 %= I; 20.1 - 40.0 % = II; 40.1 - 60.0 = III; 60.1 - 80.0% = IV; 80.1 – 100 %=V.
